# Water-assisted and protein-initiated fast and controlled ring-opening polymerization of proline *N*-carboxyanhydride

**DOI:** 10.1093/nsr/nwac033

**Published:** 2022-02-24

**Authors:** Yali Hu, Zi-You Tian, Wei Xiong, Dedao Wang, Ruichi Zhao, Yan Xie, Yu-Qin Song, Jun Zhu, Hua Lu

**Affiliations:** Beijing National Laboratory for Molecular Sciences, Center for Soft Matter Science and Engineering, Key Laboratory of Polymer Chemistry and Physics of the Ministry of Education, College of Chemistry and Molecular Engineering, Peking University, Beijing100871, China; Peking-Tsinghua Center for Life Sciences, Academy for Advanced Interdisciplinary Studies, Peking University, Beijing100871, China; Beijing National Laboratory for Molecular Sciences, Center for Soft Matter Science and Engineering, Key Laboratory of Polymer Chemistry and Physics of the Ministry of Education, College of Chemistry and Molecular Engineering, Peking University, Beijing100871, China; Beijing National Laboratory for Molecular Sciences, Center for Soft Matter Science and Engineering, Key Laboratory of Polymer Chemistry and Physics of the Ministry of Education, College of Chemistry and Molecular Engineering, Peking University, Beijing100871, China; Key Laboratory of Carcinogenesis and Translational Research (Ministry of Education), Department of Lymphoma, Peking University Cancer Hospital and Institute, Beijing100142, China; Beijing National Laboratory for Molecular Sciences, Center for Soft Matter Science and Engineering, Key Laboratory of Polymer Chemistry and Physics of the Ministry of Education, College of Chemistry and Molecular Engineering, Peking University, Beijing100871, China; Key Laboratory of Carcinogenesis and Translational Research (Ministry of Education), Department of Lymphoma, Peking University Cancer Hospital and Institute, Beijing100142, China; Key Laboratory of Carcinogenesis and Translational Research (Ministry of Education), Department of Lymphoma, Peking University Cancer Hospital and Institute, Beijing100142, China; Key Laboratory of Carcinogenesis and Translational Research (Ministry of Education), Department of Lymphoma, Peking University Cancer Hospital and Institute, Beijing100142, China; Beijing National Laboratory for Molecular Sciences, Center for Soft Matter Science and Engineering, Key Laboratory of Polymer Chemistry and Physics of the Ministry of Education, College of Chemistry and Molecular Engineering, Peking University, Beijing100871, China

**Keywords:** proline, PPII helix, ring-opening polymerization, *N*-carboxyanhydride, protein-polymer conjugates

## Abstract

The production of polypeptides via the ring-opening polymerization (ROP) of *N*-carboxyanhydride (NCA) is usually conducted under stringent anhydrous conditions. The ROP of proline NCA (ProNCA) for the synthesis of poly-_L_-proline (PLP) is particularly challenging due to the premature product precipitation as polyproline type I helices, leading to slow reactions for up to one week, poor control of the molar mass and laborious workup. Here, we report the unexpected water-assisted controlled ROP of ProNCA, which affords well-defined PLP as polyproline II helices in 2–5 minutes and almost-quantitative yields. Experimental and theoretical studies together suggest the as-yet-unreported role of water in facilitating proton shift, which significantly lowers the energy barrier of the chain propagation. The scope of initiators can be expanded from hydrophobic amines to encompass hydrophilic amines and thiol-bearing nucleophiles, including complex biomacromolecules such as proteins. Protein-mediated ROP of ProNCA conveniently affords various protein-PLP conjugates via a grafting-from approach. PLP modification not only preserves the biological activities of the native proteins, but also enhances their resistance to extreme conditions. Moreover, PLP modification extends the elimination half-life of asparaginase (ASNase) 18-fold and mitigates the immunogenicity of wt ASNase >250-fold (ASNase is a first-line anticancer drug for lymphoma treatment). This work provides a simple solution to a long-standing problem in PLP synthesis, and offers valuable guidance for the development of water-resistant ROP of other proline-like NCAs. The facile access to PLP can greatly boost the application potential of PLP-based functional materials for engineering industry enzymes and therapeutic proteins.

## Teaser

Turning water from foe to friend during the polymerization of proline N-carboxyanhydride and making the syntheses of polyproline, an important polypeptide with vast application potential, and its protein conjugate super-fast and easy.

## INTRODUCTION

Proline (Pro) is the only proteinogenic amino acid bearing a secondary amine, which results from a circular side chain that loops back and reconnects with the backbone nitrogen. This pyrrolidine ring creates a sterically hindered nitrogen and constrains the conformation of both the Pro and its preceding amino acid residue. Not surprisingly, the Pro-Pro junction is even more restricted; as an extreme case, poly-_L_-proline (PLP) is a well-known rigid ‘molecular ruler’ that exists either as all-*cis* right-handed type I (PPI) helices in common organic solvents or as all-*trans* left-handed type II (PPII) helices in aqueous solution [1,2]. In naturally occurring proteins, proline-rich regions (PRRs) in PPII helices play important roles in regulating protein–protein and protein–nucleic acid interactions, signaling, mechanical elasticity, transcriptional activation, immune response, etc. [3–6]. In materials science, there is also a rapidly growing interest in proline- or hydroxyproline-derived polymers [7–9]. PLP derivatives have also demonstrated utility as mimics of antifreeze protein [10], building blocks for hierarchical self-assembly [11], templates for controlling nanoparticle growth [12], gelators [13], molecular rulers [14], antimicrobials [15] and cell-penetrating agents [16].

**Figure 1.**
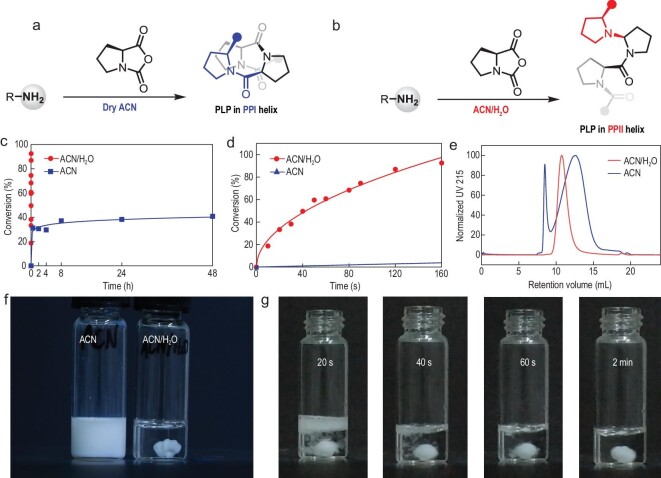
Comparison of the ROP of ProNCA in (a) pure ACN and (b) mixed ACN/H_2_O. (c) Conversions of ProNCA over time and (d) the zoomed-in period of the first 160 s. (e) SEC of the PLP produced in ACN and mixed ACN/H_2_O. (f) Photographs of the reactions in ACN (left) and mixed ACN/H_2_O (right). (g) Snapshots of the ROP of ProNCA in mixed ACN/H_2_O showing visible bubbles. [ProNCA]_0_/[I] = 100/1, [ProNCA]_0_ =_ _100 mg/mL, 10^o^C for the ROP in ACN/H_2_O and r.t. for the one in dry ACN.

Despite the aforementioned progress, research to unlock the full application potential of PPII-helical PLP has been handicapped by the difficulties in synthesizing well-defined, high-molecular-weight (*M*_n_) PLP, presumably due to the high steric hindrance and conformational constraint of Pro. This is evident even in native biosynthesis, where translation of Pro-rich sequences often induces ribosome stalling [17,18]. In chemical synthesis, the construction of a Pro-Pro junction via solid-phase peptide synthesis (SPPS) or native chemical ligation (NCL) is known to be inefficient and thus impractical for extending an oligoproline beyond 20 repeating units [19,20]. A number of early literature reports showed the ring-opening polymerization (ROP) of proline *N*-carboxyanhydride (ProNCA) to be a viable alternative, but the requirement of a strong base such as sodium methoxide inevitably resulted in racemization [21]. An additional caveat is that the *M*_n_ of PLP in these early studies was estimated solely from viscosity. Progress in the ROP of ProNCA has been achieved in several recent studies using primary or secondary amines in anhydrous pyridine, dioxane or acetonitrile (ACN) under high vacuum or a continuous nitrogen flow (Fig. 1a) [13,22,23]. Previously, the best results were reported by Gkikas *et al*., who attained a *M*_n_ up to 13 × 10^3^ g/mol and dispersity (*Đ* = *M*_w_/*M*_n_) of 1.23 based on size exclusion chromatographic (SEC) analysis in water/ACN (80/20) [22]. However, polymerization with amine-based initiators usually requires more than a week to reach 60%–80% monomer conversion in organic solvents. During the reviewing process of this work, Kramer achieved relatively faster PLP synthesis (1–2 h typically) via the use of highly efficient organometallic catalysts [24]. However, the *M*_n_ and *Đ* of the obtained PLPs were not stringently characterized using SEC due to solubility issues. Moreover, a major flaw of all current methods is that PLP would precipitate prematurely in organic solvent in the form of PPI helices. As a result, time-consuming dialysis would be needed after the reaction to obtain water-soluble, PPII-helical PLP. Understandably, the inefficient synthetic method has proved to be a bottleneck for the biomedical and material application of PLP.

Controlled methodologies for the ROP of *N*-carboxyanhydrides (NCAs) have bloomed in the past decade [25–35]. While the majority of these systems require stringent anhydrous conditions, in recent years the focus has been shifting toward achieving ROP in open vessels with aqueous solution [36–39]. To name a few, Cheng [37], Heise [38] and Lecommandoux and Bonduelle [39] have demonstrated the feasibility of controlled ROP of NCAs at the water–oil interface, in oil-in-water emulsion, or even in pure aqueous solutions. These pioneering works opened up a new avenue for developing water-tolerant ROP of NCAs. However, until now, aqueous-phase ROP of NCAs that generates PLP or other water-soluble polypeptides remains elusive.

Herein, we report the production of well-defined PLP with predictable *M*_n_s up to 18.7 × 10^3^ g/mol and *Đ* in the range of 1.1–1.2, via the unexpected amine-initiated, water-assisted ROP of ProNCA in mixed ACN/H_2_O (Fig. 1b). In sharp contrast to previous methods, which typically require weeks to obtain PPII-helical PLP, we accomplished complete monomer conversion to product without induction in <5 min, sometimes even within one minute. Density functional theory (DFT) calculations of the model chain propagation reaction revealed that water played an as-yet-unreported role in assisting both the nucleophilic attack and hydrogen migration, which reduced the overall energy barrier of the polymerization by 7.1 kcal/mol. We also demonstrated that our method could be applied to the synthesis of protein-PLP conjugates via the site-specific grafting-to or randomly labeled grafting-from approach. Importantly, the PLP modification dramatically enhanced the stability of DHFR (dihydrofolate reductase) under extreme conditions while maintaining its enzymatic activity. Moreover, PLP modification prolonged the circulation time and mitigated immunogenicity of a therapeutic protein in a way that outperformed the well-established PEGylation. Together, these results highlight the enormous potential of PLP as a fully ‘natural’ and degradable polypeptide for industrial and biomedical applications.

## RESULTS

Highly pure ProNCA was prepared using a moisture-resistant method that our lab recently developed [40], which significantly simplified the conventional procedure that involves laborious workup. The application of this method also improved the yield to 72%, up from 30% reported previously. We began our study by first performing primary amine-initiated ROP of ProNCA in dry ACN in a glovebox. Kinetic characterization suggested, rather unexpectedly, that the reaction began with an initial stage of fast polymerization with monomer conversion rapidly reaching ∼30% in 30 min, followed by a second stage in which another 10% of the monomer was slowly consumed over 2 days (Fig. 1c and d, blue curves). SEC analysis of the PLPs obtained from different reaction time points consistently showed bimodal peaks with no significant increase in *M*_n_ after 30 min of polymerization (Fig. 1e, blue curve, and Fig. S1). Because the precipitation of PLP occurred shortly after the addition of initiator (Fig. 1f), we hypothesized that this sequestered the growing PLP chain from the organic phase (i.e. ACN), leading to slow, uncontrolled polymerization that was the hallmark of the second stage. We further hypothesized that the above problem could be solved by adding water to the reaction to form a homogenous mixture. We thus tested eight common organic solvents, each mixed with water (*v*/*v* = 1/1) to serve as the solvent for the ROP of ProNCA. To our great surprise, polymerization in ACN/H_2_O was extraordinarily fast (Fig. 1c and d, red curves), seemed to be well-controlled (Fig. 1e, red curves) and gave a clear solution without precipitation (Fig. 1f). Remarkably, the reaction consumed almost all monomer in <2 min at 10^o^C, as evidenced by the generation of visible CO_2_ bubbles immediately after the initiator was added (Fig. 1g and supplementary video). A slightly slower ROP was observed in tetrahydrofuran/H_2_O, whereas all other solvent combinations led to bimodal/broader SEC traces (Fig. S2).

Subsequent reaction optimization in ACN/H_2_O yielded the following findings. First, unimodal SEC traces could be obtained at all tested temperatures in the range of 0 to 65^o^C, with an inverse correlation between *M*_n_ and temperature (Fig. S3). Second, pH variation between 5.0 and 9.0 seemed to have negligible effect on the *M*_n_ (Fig. S4). Third, an initial monomer concentration ([ProNCA]_0_) above 50 mg/mL was necessary to ensure both a fast reaction and good *M*_n_ control (Fig. S5). Fourth, an H_2_O content of 40%–60% was optimal (Fig. S6); insufficient H_2_O would not completely dissolve PLP, whereas too much H_2_O appeared to be detrimental to *M*_n_ control, likely due to the increased reaction competition from monomer hydrolysis. Fifth, ionic strength (i.e. concentration of NaCl) did not play a significant role in regulating the distribution of *M*_n_ (Fig. S7).

**Figure 2.**
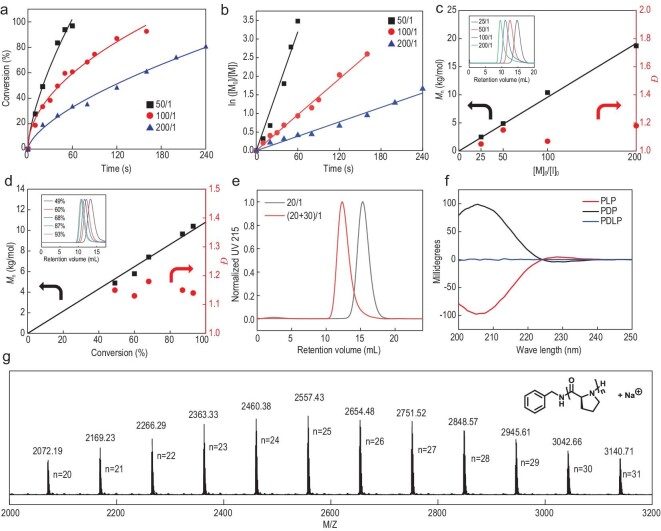
Controlled ROP of ProNCA in mixed ACN/H_2_O mediated by benzyl amine. (a) Conversion of ProNCA over time and (b) plots of first-order kinetics of the ROP at different [ProNCA]_0_/[I]_0_ ratios in ACN-*d*_3_/D_2_O (1/1); [ProNCA]_0 _= 100 mg/mL. (c, d) Plots of *M*_n_ and *Đ* of PLP, (c) as a function of [ProNCA]_0_/[I]_0_ ratio and (d) conversion of ProNCA ([ProNCA]_0_/[I]_0 _= 100/1). (e) SEC traces showing the chain extension of PLP from a 20-mer to a 50-mer. (f) MALDI-TOF mass spectrum of PLP 25-mer. (g) CD spectra of PLP, PDP and PDLP showing typical left-handed and right-handed PPII helices, and no secondary conformation, respectively.

We next characterized the reaction kinetics, *M*_n_, *Đ*, end group and chain extension to examine whether the ROP was fully living/controllable (Fig. 2). The polymerization was found to follow first-order kinetics (Fig. 2a and b and Figs S8–10) versus monomer concentration at various monomer-to-initiator molar ratios ([ProNCA]_0_/[I]). The chain propagation rate constant (*k*_p_) was estimated to be 1.95–4.00 M^–1^ s^–1^. Of note, primary amine-initiated ROP of NCAs produced *k*_p_ values typically in the range of 10^–3^–10^–2^ M^–1^ s^–1^ [27,41,42]. Varying the feeding [ProNCA]_0_/[I] ratio led to a linear increase in the *M*_n_ of the PLP products, which were within 5% deviation from the theoretical values (Fig. 2c and Table 1, entries 2–5). PLPs of various *M*_n_ all displayed unimodal SEC peaks, with *Đ* in the range of 1.1–1.2 (Fig. 2c inset and Table 1, entries 2–5). The highest obtained *M*_n_ was 18.7 × 10^3^ g/mol, which was ∼5% lower than its expected *M*_n_ at a feeding [ProNCA]_0_/[I] ratio of 200/1 (Table 1, entry 5). Nevertheless, the SEC trace of PLP_200_ showed a small tail at the lower *M*_n_ region, suggesting the *M*_n_ control reached its upper limit. The linear correlation between *M*_n_ and the degree of monomer conversion was characteristic of chain-growth polymerization (Fig. 2d). The livingness of the ROP was further confirmed by the facile chain extension from an *in-situ*-generated PLP macroinitiator (Fig. 2e). Matrix-assisted laser desorption/ionization-time of flight (MALDI-TOF) mass spectrometric analysis of the PLP product from benzyl amine-mediated ROP at a [ProNCA]_0_/[I] ratio of 25/1 revealed a well-defined end group fidelity, where all peaks could be assigned to the molecular formula of BnNH–Pro_n_–H* *+ Na^+^ and none belonged to the chain initiation from hydrolyzed proline (Fig. 2f)*.* Compared to the conventional method, which requires time-consuming dialysis to induce an incomplete PPI-to-PPII transition of the PLP product (Table 1, entry 1), the ROP in mixed ACN/H_2_O allowed the direct acquisition of water-soluble PLP at 83%–92% purification yield (Table 1, entries 2–11). Circular dichroism (CD) spectroscopy confirmed that the PLP prepared in mixed ACN/H_2_O existed as left-handed PPII helices (Fig. 2g), whereas the ROP of _D_-ProNCA and _DL_-ProNCA furnished right-handed PPII-helical poly-_D_-proline (PDP) and disordered poly-_DL_-proline (PDLP) products, respectively (Fig. 2g).

**Table 1.** ROP of ProNCAs in mixed ACN/H_2_O.

Entry	Initiator	Solvent	[ProNCA]_0_/[I]	Time	*M*_n_^cal^ (kg/mol)^a^	*M*_n_^obt^ (kg/mol)^b^	*Đ* ^b^	Yield (%)^c^	[*Θ*]_229_ (deg cm^2^ dmol^–1^)
1	Benzylamine	ACN	100	7 days	9.8	/^d^	/^d^	48	1381
2	Benzylamine	ACN/H_2_O	25	<1 min	2.5	2.5	1.05	85	2081
3	Benzylamine	ACN/H_2_O	50	<2 min	5.0	5.0	1.15	89	2126
4	Benzylamine	ACN/H_2_O	100	<3 min	9.8	10.3	1.07	88	2226
5	Benzylamine	ACN/H_2_O	200	<5 min	19.5	18.7	1.18	92	2393
6	Benzylamine	ACN/H_2_O	50	<2 min	5.0	5.1	1.10	83	−2354^e^
7	Benzylamine	ACN/H_2_O	50	<2 min	5.0	4.6	1.12	87	32^f^
8	Diethyl amine	ACN/H_2_O	50	<2 min	5.0	7.6	1.05	90	2762
9	Phenyl amine	ACN/H_2_O	50	<2 min	5.0	5.5	1.12	86	2227
10	Thiophenol	ACN/H_2_O	50	<2 min	5.0	4.9	1.15	86	2246
11	Glucosamine	ACN/H_2_O	50	<2 min	5.0	5.8	1.07	83	2796

^a^Calculated from feeding [ProNCA]_0_/[I]_0_. ^b^Obtained from aqueous SEC equipped with multi-angle light scattering and reflective index detectors in 1 × PBS (pH = 7.4) mobile phase; d*n*/d*c* (658 nm) values were measured as 0.178 for PLP and 0.175 for PDLP. ^c^Separation yield after PD-10 column purification. ^d^SEC gave bimodal peaks as shown in Fig. 1e. ^e^_D-_ProNCA was used as monomer. ^f^_DL-_ProNCA was used as monomer.

The enhanced solubility of PLP in water was certainly a contributing factor to the faster ROP of ProNCA in ACN/H_2_O. However, a significant rate increase was also observed when the content of water was only 20% during our condition optimization (Fig. S6), at which the PLP was still insoluble. Moreover, the ROP was markedly accelerated even when only 2% H_2_O was added to ACN (Fig. S11). Collectively, these results indicated that the contribution of water must involve additional mechanisms apart from solubility improvement and an increase in solvent polarity/dielectric constant. We first ruled out the possible contribution of ProNCA hydrolysis based on two facts: (i) the MALDI-TOF spectrum displayed only the expected end group (Fig. 2f), which suggested very little initiation by hydrolyzed proline, and (ii) ProNCA remained intact for >4 min under the ROP conditions without an amine initiator (Fig. S12), which was longer than the time required for reaching complete ROP. We next disapproved the existence of cooperative polymerization, a self-accelerating mechanism that was elegantly demonstrated by Cheng and co-workers, in the ROP of NCAs, to produce α-helical polypeptides [42]. No induction period that corresponded to the nucleation stage, a well-known kinetic feature of cooperative polymerization, was observed in any of our kinetic studies (Fig. 2a and b). Moreover, there was no significant difference in the polymerization rate or *M*_n_ control between the ROP of _L-, D-_ and _DL_-ProNCA, regardless of whether the reaction was initiated by benzyl amine (Table 1, entries 3, 6 and 7) or the macroinitiator PLP (Fig. S13).

**Figure 3.**
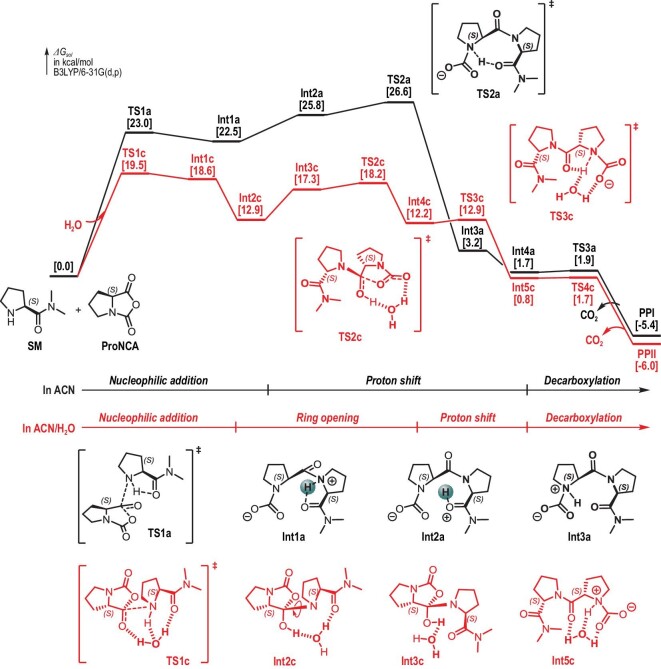
DFT calculations of model chain propagation reactions reveal plausible pathways for the ROP of ProNCA in dry ACN (black) and mixed ACN/H_2_O (red).

Interestingly, careful kinetic study revealed that the ROP was faster in 2% H_2_O/ACN than in 2% D_2_O/ACN (Fig. S11). This isotope effect implied that water molecules might be involved in the rate-determining step (RDS), likely through hydrogen bonding or facilitating proton shift. We tested this hypothesis by conducting a detailed DFT study of a model chain-propagation reaction (Fig. 3) using (*S*)-*N*, *N*-dimethylpyrrolidine-2-carboxamide (SM) to represent the reactive chain end. It was found that the reaction in pure ACN proceeded as a three-stage process that consisted of nucleophilic addition, intramolecular proton shift and decarboxylation. The transition state **TS2a** showed the highest activation Gibbs free energy of 26.6 kcal/mol, leading to the surprising finding that the proton shift, rather than the nucleophilic attack, was the RDS for the ROP of ProNCA in pure ACN (Fig. 3 and Figs S14–15). In the presence of H_2_O, however, the same model reaction above was found to follow an alternative mechanistic route that could be roughly divided into four stages, including nucleophilic addition, ring-opening, intramolecular proton shift and decarboxylation. A key deviation from the ROP in anhydrous ACN was that H_2_O, both as a hydrogen-bonding donor and acceptor, helped arrange SM and ProNCA in proximity to each other, which facilitated the nucleophilic addition (Fig. 3 and Figs S16–17, **TS1c)**. The activation Gibbs free energy of **TS1c** was 19.5 kcal/mol, which was the highest energy barrier for the whole process. Importantly, hydrogen bonding to water molecules stabilized the amide bond of the zwitterionic intermediate **Int4c** in a *trans* conformation, and facilitated the subsequent proton shift, leading to the final, spontaneous decarboxylation of **Int5c**. Overall, the presence of H_2_O shifted the RDS toward nucleophilic addition, and dramatically lowered the overall energy barrier of the chain propagation by 7.1 kcal/mol, which could increase the rate of ROP by up to five orders of magnitude (Fig. 3).

**Figure 4.**
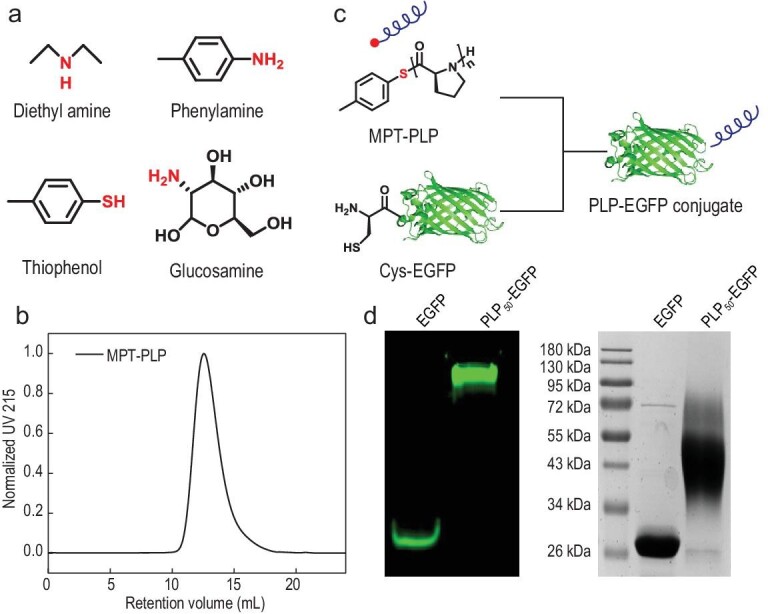
(a) Scope of small molecular initiators for the ROP of ProNCA in mixed ACN/H_2_O. (b) The SEC trace of MPT-PLP_50_. (c) Scheme of PLP-EGFP synthesis via the site-specific NCL of MPT-PLP and Cys-EGFP. (d) Native (left) and SDS-PAGE (right) analysis of the purified PLP-EGFP conjugate.

To explore the scope of initiators, diethyl amine, *p*-methyl phenyl amine, *p*-methyl phenyl mercaptane and complex water-soluble biomolecules such as glucosamine (Fig. 4a) were tested and all mediated fast ROP of ProNCA under the optimized conditions. Among these initiators, *p*-methyl phenyl mercaptane, *p*-methyl phenyl amine and glucosamine afforded polymers with the predicted *M*_n_, whereas the *M*_n_ of the diethyl amine-based polymer appeared to be slightly higher than expected (Table 1, entries 8–11). For example, SEC analysis of the PLP mediated by *p*-methyl phenyl mercaptane (MPT-PLP_50_) had an *M*_n_ of 4.9 kg/mol and *Đ* of 1.15 at the feeding [ProNCA]_0_/[I] ratio of 50/1 (Fig. 4b), which was almost identical to the expected *M*_n_. MALDI-TOF spectrometry and ^1^H NMR spectroscopic analyses of these PLPs all revealed well-defined end groups (Figs S18–21). Notably, native chemical ligation (NCL) of MPT-PLP_50_ with Cys-EGFP (enhanced green fluorescent protein bearing an *N*-cysteine) yielded the site-specific PLP-EGFP conjugate in 73% yield under mild conditions (Fig. 4c and d) [8,43]. This result was remarkable as proline alkyl thioester was well-known for its low reactivity for NCL.

**Figure 5.**
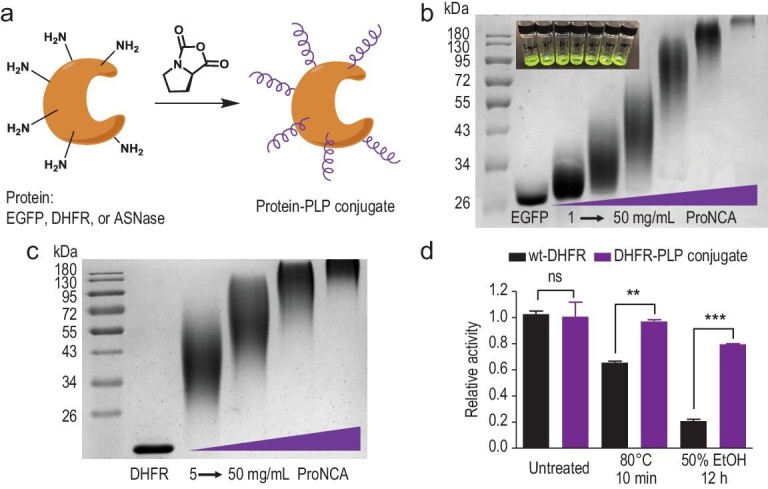
Protein-initiated ROP of ProNCA and stability of the Protein-PLP conjugates. (a) Cartoon illustration of the protein-initiated ROP of ProNCA. (b) SDS-PAGE characterization; inset: snapshots of the EGFP-PLP conjugates. (c) SDS-PAGE characterization of the DHFR-PLP conjugates. (d) DHFR enzymatic assay under normal or extreme conditions for wt-DHFR (black bars) and the DHFR-PLP conjugate (purple bars). *P* value was determined by ANOVA (ns = no significant difference; ^*^P < 0.05; ^**^P < 0.01; ^***^P < 0.001).

Encouraged by the robustness of the ROP at various pH and salt concentrations (Figs S4 and S7), we further examined whether amine-bearing proteins could be utilized as macroinitiators to generate protein-PLP conjugates *in situ* (Fig. 5a) [44–51]. The protein-initiated ROP of various NCAs, including ProNCA, was previously explored in the 1950s and 1960s [52,53]. Here, we decided to revisit this topic thanks to the ultra-fast kinetics in mixed ACN/H_2_O. Gratifyingly, 10-min ROP of ProNCA at 10^o^C in an ACN/phosphate buffer saline (PBS) (*v*/*v* = 1/1, pH 7.4) solution of EGFP led to the efficient formation of EGFP-PLP conjugates based on sodium dodecyl sulfate polyacrylamide gel electrophoresis (SDS-PAGE) analysis. When the feeding concentration of ProNCA was increased from 1.0 to 50 mg/mL, the products, appearing as smears, gradually shifted to higher molecular weights, with the upper limit exceeding 180 kDa (Fig. 5b). The average molecular weight of the EGFP-PLP conjugates was close to the theoretically predicted value, and no free EGFP was observed in the crude reaction mixture. These results were consistent with quantitative initiation efficiency and controlled PLP growth under these conditions. Of note, the fluorescence of EGFP was well maintained throughout the ROP and purification (Fig. 5b, inset). The ROP of PLP using dihydrofolate reductase (DHFR) as initiator yielded a product profile with a similar molecular weight distribution (Fig. 5c). Moreover, the resulting three DHFR-PLP conjugates exhibited almost identical enzymatic activity as untreated wt-DHFR (Fig. S23). Remarkably, one DHFR-PLP conjugate was found to retain ∼80% or 96% of the enzymatic activity of the native protein after 12 h of ethanol treatment or 10 min of heat shock at 80^o^C. In contrast, wt-DHFR retained only 20% and 65% of the catalytic activity after the same ethanol and heat treatment, respectively (Fig. 5d).

**Figure 6.**
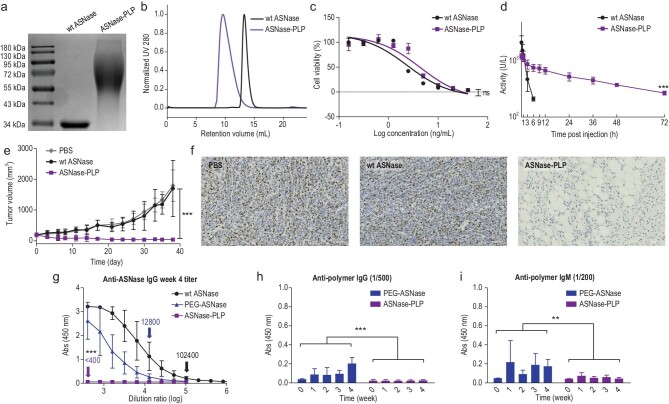
Pharmacological properties and immunogenicity of the ASNase-PLP conjugate. (a) SDS-PAGE and (b) aqueous SEC characterization of ASNase-PLP prepared by ASNase-mediated ROP of ProNCA. (c) *In vitro* cytotoxicity of ASNase-PLP on a human NK/T lymphoma cell line NKYS; *n* = 3. (d) Pharmacokinetic profile of intravenously infused ASNase-PLP in SD rats; *n* = 4; the enzymatic activity, obtained using Nessler's reagent (Merck, Germany), was employed to measure the plasma concentration of ASNase. (e) NKYS tumor growth curves and (f) immunohistochemistry (anti-human ki67) of tumor sections after receiving biweekly intraperitoneal treatments of PBS, wt ASNase (15 U/mouse) or ASNase-PLP (15 U/mouse). Scale bars, 50 μm. Six-week-old B-NDG mice were subcutaneously inoculated with NKYS cells (6.0 × 10^6^) and randomized (*n* = 8 for each group) when the tumors reached ∼300 mm^3^ (day 0). (g) ELISA analysis of the titers of anti-ASNase IgG in the week-4 antisera drawn from wt ASNase-, ASNase-PEG- or ASNase-PLP-infused SD rats. (h, i) ELISA analysis of the changes of anti-polymer (h) IgG and (i) IgM levels in the antisera drawn from wt ASNase-, ASNase-PEG- or ASNase-PLP-infused SD rats. For the immunization, SD rats (*n* = 4 for each group) were subcutaneously infused with wt ASNase, ASNase-PEG or ASNase-PLP (200 U/kg) once every week for totally four weeks. Antisera were drawn every week before each injection. For the ELISA assay, the plates were coated with (g) wt ASNase, a PLP-interferon conjugate (to detect anti-PLP antibodies) and (h, i) a PEG-interferon conjugate (to detect anti-PEG antibodies). Data are represented as mean ± SD. *P* values are determined by ANOVA (ns = no significant difference; ^*^P < 0.05; ^**^P < 0.01; ^***^P < 0.001).

Inspired by these results, we next tested whether the growth of PLP on a therapeutic protein could improve its pharmacological properties. Asparaginase (ASNase) is a first-line enzyme-based anticancer drug for acute lymphatic leukemia and several aggressive subtypes of lymphoma, but is known for its notoriously poor pharmacokinetic profiles and strong immunogenicity, which necessitates polymer conjugation [54–56]. For this, we prepared an ASNase-PLP conjugate via the same grafting-from approach described above. Both SDS-PAGE and SEC characterizations confirmed that the ASNase-PLP conjugate was successfully synthesized and had a relatively uniform size (Fig. 6a and b). *In vitro*, the purified ASNase-PLP conjugate showed a half-inhibition concentration (IC_50_) of 4.3 ng/mL on the human NK/T lymphoma cell line NKYS (Fig. 6c), showing only a slight decrease in potency compared to its wild-type counterpart (IC_50_:2.4 ng/mL, no statistical significance). *In vivo*, ASNase-PLP injected via tail vein exhibited a significantly prolonged blood elimination half-life (*t*_1/2β_) of 26 h in SD rats, which was 18 times that of wt ASNase (Fig. 6d). More interestingly, in mice implanted with ∼300 mm^3^ NKYS tumors, biweekly treatment of ASNase-PLP almost completely inhibited the tumor growth, and eventually led to its shrinkage, while wt ASNase produced almost no antitumor effect at the same dose and frequency of administration (Fig. 6e). Ki-67 staining of the tumor tissues confirmed that only ASNase-PLP treatment led to significant tumor killing (Fig. 6f). All mice exhibited remarkable tolerance of the drug conjugate at the applied dose based on the tracking of body weight (Fig. S24) and histological assessment of major organs (Fig. S25). These results vividly showed that extending the blood half-life of ASNase could significantly improve its antitumor efficacy *in vivo*. ASNase is a bacterial enzyme with extremely high immunogenicity, and the related side effects cannot be completely eliminated in clinical practice even through PEG modification. More worrisome is the hapten nature of PEG itself, which induces the production of anti-PEG antibodies after coupling with ASNase, which in turn leads to hypersensitivity and an accelerated blood clearance (ABC) effect. To interrogate whether PLP conjugation was a better alternative, we injected the rats with ASNase-PLP or ASNase-PEG subcutaneously once a week and collected antisera each time before the injection. Enzyme linked immunosorbent assay (ELISA) analysis revealed that the titer of anti-ASNase IgG in the ASNase-PLP antisera harvested at week four was <400, compared to 12 800 and 102 400 for the antisera of ASNase-PEG and wt ASNase, respectively (Fig. 6g). Moreover, the levels of anti-polymer IgM and IgG in the ASNase-PLP antiserum were also significantly lower than in ASNase-PEG antiserum (Fig. 6h and i).

## DISCUSSION AND CONCLUSIONS

PPII helices are among the most prevalent structural and functional motifs in proteins. Synthetic PPII-helical PLP can emulate the unique structural and functional roles of PPII helices, ultimately leading to the creation of novel biochemical materials. However, conventional methods for PLP synthesis are slow and inefficient, require stringent moisture-free conditions, allow very little control of *M*_n_ and *Đ*, and often result in premature product precipitation as PPI helices. In the current study, we demonstrated that controlled and dramatically accelerated ROP of ProNCA could be easily achieved by simply adding water as a co-solvent (Fig. 1). Notably, to obtain accurate *M*_n_ and *Đ* information of the PLP, we employed an aqueous SEC system equipped with both multi-angle light scattering (MALS) and refractive index detectors, which allowed us to acquire the d*n*/d*c* value of the PLP and calculate the absolute *M*_n_. One notable limitation of many previous methods is that the scope of initiators was restricted to hydrophobic amines. This limitation was easily overcome in this study for the obvious reason that water is a good solvent for many small molecules and biomacromolecules. Indeed, we successfully expanded the scope of initiators to hydrophilic amine- and thiol-bearing nucleophiles, including complex biomacromolecules such as proteins (Figs 4–6).

Moisture-tolerant ROP of NCAs has gained considerable attention in recent years with many notable advances. The unique feature of our work is that water played multiple beneficial roles in our reaction system rather than being a detrimental factor. Key contributing factors to the excellent reaction control that we observed included enhanced solubility of PLP in water and the unexpected water-assisted acceleration of the ROP, which allowed it to kinetically outcompete the monomer hydrolysis. DFT results revealed the crucial mechanistic role of water molecules in facilitating a hydrogen shift that lowered the energy barrier of the chain propagation by a substantial ∼7.1 kcal/mol (Fig. 3). However, it should be noted that at [ProNCA]_0_/[I] ratios higher than 200/1, the hydrolysis of ProNCA became significant.

Synthetic polypeptides have been used for protein conjugation, i.e. protein PEPylation, for improved pharmacological properties [43,51,54,57–61]. But the function of PLP for protein modification is yet to be fully exploited. Here, we showed that PLP was effective in protecting the conjugated protein from denaturation under harsh conditions such as high temperature and exposure to organic solvents. Interestingly, the PLP-grafted EGFP fully preserved the EGFP fluorescence and DHFR catalytic activity. There are three plausible reasons for this result. First, the fast kinetics of ROP allowed product generation within 10 min, and thus minimized the detrimental effects posed by exposure to the organic solvent. Second, it appeared that the growth of PLP on the surface of DHFR enhanced the resistance to organic solvent dramatically (Fig. 5d). Third, the substrates of the DHFR-catalyzed reaction were small molecules, whose diffusion and binding to the catalytic pocket was generally insensitive to the steric hindrance of PLP. Although this grafting-from approach might not be suitable for proteins whose biological functions are dependent on lysine(s) in the active pocket, it could still find broad application in a variety of proteins whose functions are lysine independent. For instance, our results showed that PLP modification substantially improved the pharmacokinetic properties of ASNase while largely preserving its catalytic activity, which eventually led to enhanced *in vivo* antitumor efficacy. Most interestingly, our results unambiguously proved that PLP could not only reduce the immunogenicity of the protein covalently attached to it, but its antigenicity *per se* was also lower than PEG. These results indicate that PLP, which is neutral in charge, biodegradable and completely natural, holds great potential as an outstanding alternative to the non-degradable PEG, whose clinical limitations have been increasingly recognized in recent years [54,60,62–65].

Overall, this work provides a simple means of solving a longstanding problem in PLP synthesis and offers valuable guidance for the development of water-resistant ROP of other NCAs. The availability of PLP could facilitate our understanding of the biophysical and biological roles of PRRs in natural proteins and greatly boost the application potential of PLP-based functional materials for engineering of industrial enzymes and therapeutic proteins.

## Supplementary Material

nwac033_Supplemental_File

## References

[1] Best RB , Merchant KA, Gopich IV et al. Effect of flexibility and *cis* residues in single-molecule FRET studies of polyproline. Proc Natl Acad Sci USA 2007; 104: 18964–9. 10.1073/pnas.0709567104 18029448 PMC2141891

[2] Schuler B , Lipman EA, Steinbach PJ et al. Polyproline and the “spectroscopic ruler” revisited with single-molecule fluorescence. Proc Natl Acad Sci USA 2005; 102: 2754–9. 10.1073/pnas.0408164102 15699337 PMC549440

[3] Kay BK , Williamson MP, Sudol P. The importance of being proline: the interaction of proline-rich motifs in signaling proteins with their cognate domains. FASEB J 2000; 14: 231–41. 10.1096/fasebj.14.2.231 10657980

[4] Mahoney NM , Janmey PA, Almo SC. Structure of the profilin-poly-L-proline complex involved in morphogenesis and cytoskeletal regulation. Nat Struct Biol 1997; 4: 953–60. 10.1038/nsb1197-953 9360613

[5] Yu HT , Chen JK, Feng SB et al. Structural basis for the binding of proline-rich peptides to SH3 domains. Cell 1994; 76: 933–45. 10.1016/0092-8674(94)90367-0 7510218

[6] Ruggiero MT , Sibik J, Orlando R et al. Measuring the elasticity of poly-L-proline helices with terahertz spectroscopy. Angew Chem Int Ed 2016; 55: 6877–81. 10.1002/anie.201602268 PMC4999051 27121300

[7] Tian Z-Y , Wang S, Lu H. Hydroxyproline-derived biomimetic and biodegradable polymers. Curr Opin Solid State Mater Sci 2021; 25: 100902. 10.1016/j.cossms.2021.100902

[8] Yuan J , Shi D, Zhang Y et al. 4-Hydroxy-L-proline as a general platform for stereoregular aliphatic polyesters: controlled ring-opening polymerization, facile functionalization, and site-specific bioconjugation. CCS Chem 2020; 2: 236–44. 10.31635/ccschem.020.201900119

[9] Yuan J , Xiong W, Zhou X et al. 4-Hydroxyproline-derived sustainable polythioesters: controlled ring-opening polymerization, complete recyclability, and facile functionalization. J Am Chem Soc 2019; 141: 4928–35. 10.1021/jacs.9b00031 30892027

[10] Graham B , Bailey TL, Healey JRJ et al. Polyproline as a minimal antifreeze protein mimic that enhances the cryopreservation of cell monolayers. Angew Chem Int Ed 2017; 56: 15941–4. 10.1002/anie.201706703 PMC5722203 29044869

[11] Gkikas M , Haataja JS, Seitsonen J et al. Extended self-assembled long periodicity and zig-zag domains from helix-helix diblock copolymer poly(gamma-benzyl-L-glutamate)-block-poly(O-benzyk-hydroxyproline). Biomacromolecules 2014; 15: 3923–30. 10.1021/bm5009734 25260019

[12] Upert G , Bouillere F, Wennemers H. Oligoprolines as scaffolds for the formation of silver nanoparticles in defined sizes: correlating molecular and nanoscopic dimensions. Angew Chem Int Ed 2012; 51: 4231–4. 10.1002/anie.201107183 22213611

[13] Gkikas M , Avery RK, Olsen BD. Thermoresponsive and mechanical properties of poly(L-proline) gels. Biomacromolecules 2016; 17: 399–406. 10.1021/acs.biomac.5b01168 26736072 PMC4747850

[14] Dobitz S , Aronoff MR, Wennemers H. Oligoprolines as molecular entities for controlling distance in biological and material sciences. Acc Chem Res 2017; 50: 2420–8. 10.1021/acs.accounts.7b00340 28885830

[15] Kuriakose J , Hernandez-Gordillo V, Nepal M et al. Targeting intracellular pathogenic bacteria with unnatural proline-rich peptides: coupling antibacterial activity with macrophage penetration. Angew Chem Int Ed 2013; 52: 9664–7. 10.1002/anie.201302693 23960012

[16] Fillon YA , Anderson JP, Chmielewski J. Cell penetrating agents based on a polyproline helix scaffold. J Am Chem Soc 2005; 127: 11798–803. 10.1021/ja052377g 16104758

[17] Tanner DR , Cariello DA, Woolstenhulme CJ et al. Genetic identification of nascent peptides that induce ribosome stalling. J Biol Chem 2009; 284: 34809–18. 10.1074/jbc.M109.039040 19840930 PMC2787343

[18] Pavlov MY , Watts RE, Tan Z et al. Slow peptide bond formation by proline and other N-alkylamino acids in translation. Proc Natl Acad Sci USA 2009; 106: 50–4. 10.1073/pnas.0809211106 19104062 PMC2629218

[19] Sayers J , Karpati PMT, Mitchell NJ et al. Construction of challenging proline-proline junctions via diselenide-selenoester ligation chemistry. J Am Chem Soc 2018; 140: 13327–34. 10.1021/jacs.8b07877 30239198

[20] Townsend SD , Tan ZP, Dong SW et al. Advances in proline ligation. J Am Chem Soc 2012; 134: 3912–6. 10.1021/ja212182q 22332757 PMC3306728

[21] Fasman GD , Blout ER. High molecular weight poly-L-proline: synthesis and physical-chemical studies. Biopolymers 1963; 1: 3–14. 10.1002/bip.360010103

[22] Gkikas M , Iatrou H, Thomaidis NS et al. Well-defined homopolypeptides, copolypeptides, and hybrids of poly(L-proline). Biomacromolecules 2011; 12: 2396–406. 10.1021/bm200495n 21568310

[23] Muller D , Stulz J, Kricheldorf HR. Secondary structure of peptides 14. FT-IR and 13C NMR CP/MAS investigation of the helix stability of solid poly(L-proline)s. Makromol Chem 1984; 185: 1739–49. 10.1002/macp.1984.021850820

[24] Detwiler RE , Schlirf AE, Kramer JR. Rethinking transition metal catalyzed N-carboxyanhydride polymerization: polymerization of Pro and AcOPro N-carboxyanhydrides. J Am Chem Soc 2021; 143: 11482–9. 10.1021/jacs.1c03338 34283588

[25] Zhao W , Lv Y, Li J et al. Fast and selective organocatalytic ring-opening polymerization by fluorinated alcohol without a cocatalyst. Nat Commun 2019; 10: 3590. 10.1038/s41467-019-11524-y 31399569 PMC6689068

[26] Deming TJ . Facile synthesis of block copolypeptides of defined architecture. Nature 1997; 390: 386–9. 10.1038/37084 9389476

[27] Yuan J , Sun Y, Wang J et al. Phenyl trimethylsilyl sulfide-mediated controlled ring-opening polymerization of alpha-amino acid N-carboxyanhydrides. Biomacromolecules 2016; 17: 891–6. 10.1021/acs.biomac.5b01588 26796118

[28] Lu H , Cheng JJ. Hexamethyldisilazane-mediated controlled polymerization of alpha-amino acid N-carboxyanhydrides. J Am Chem Soc 2007; 129: 14114–5. 10.1021/ja074961q 17963385

[29] Vacogne CD , Schlaad H. Primary ammonium/tertiary amine-mediated controlled ring opening polymerisation of amino acid N-carboxyanhydrides. Chem Commun 2015; 51: 15645–8. 10.1039/C5CC06905J 26359317

[30] Conejos-Sanchez I , Duro-Castano A, Birke A et al. A controlled and versatile NCA polymerization method for the synthesis of polypeptides. Polym Chem 2013; 4: 3182–6. 10.1039/c3py00347g

[31] Rasines Mazo A , Allison-Logan S, Karimi F et al. Ring opening polymerization of alpha-amino acids: advances in synthesis, architecture and applications of polypeptides and their hybrids. Chem Soc Rev 2020; 49: 4737–834. 10.1039/C9CS00738E 32573586

[32] Liu Y , Li D, Ding J et al. Controlled synthesis of polypeptides. Chin Chem Lett 2020; 31: 3001–14. 10.1016/j.cclet.2020.04.029

[33] Tao XF , Li MH, Ling J. Alpha-amino acid N-thiocarboxyanhydrides: a novel synthetic approach toward poly(alpha-amino acid)s. Eur Polym J 2018; 109: 26–42. 10.1016/j.eurpolymj.2018.08.039

[34] Deming TJ . Synthesis of side-chain modified polypeptides. Chem Rev 2016; 116: 786–808. 10.1021/acs.chemrev.5b00292 26147333

[35] Salas-Ambrosio P , Tronnet A, Since M et al. Cyclic poly(α-peptoid)s by lithium bis(trimethylsilyl)amide (LiHMDS)-mediated ring-expansion polymerization: simple access to bioactive backbones. J Am Chem Soc 2021; 143: 3697–702. 10.1021/jacs.0c13231 33651603

[36] Wu Y , Zhang D, Ma P et al. Lithium hexamethyldisilazide initiated superfast ring opening polymerization of alpha-amino acid N-carboxyanhydrides. Nat Commun 2018; 9: 5297. 10.1038/s41467-018-07711-y 30546065 PMC6294000

[37] Song Z , Fu H, Wang J et al. Synthesis of polypeptides via bioinspired polymerization of in situ purified N-carboxyanhydrides. Proc Natl Acad Sci USA 2019; 116: 10658–63. 10.1073/pnas.1901442116 31088971 PMC6561217

[38] Jacobs J , Pavlović D, Prydderch H et al. Polypeptide nanoparticles obtained from emulsion polymerization of amino acid N-carboxyanhydrides. J Am Chem Soc 2019; 141: 12522–6. 10.1021/jacs.9b06750 31348858

[39] Grazon C , Salas-Ambrosio P, Ibarboure E et al. Aqueous ring-opening polymerization-induced self-assembly (ROPISA) of N-carboxyanhydrides. Angew Chem Int Ed 2020; 59: 622–6. 10.1002/anie.201912028 31650664

[40] Tian Z-Y , Zhang Z, Wang S et al. A moisture-tolerant route to unprotected α/β-amino acid N-carboxyanhydrides and facile synthesis of hyperbranched polypeptides. Nat Commun 2021; 12: 5810. 10.1038/s41467-021-25689-y 34608139 PMC8490447

[41] Zou J , Fan JW, He X et al. A facile glovebox-free strategy to significantly accelerate the syntheses of well-defined polypeptides by N-carboxyanhydride (NCA) ring-opening polymerizations. Macromolecules 2013; 46:4223–6. 10.1021/ma4007939 23794753 PMC3686519

[42] Baumgartner R , Fu HL, Song ZY et al. Cooperative polymerization of alpha-helices induced by macromolecular architecture. Nat Chem 2017; 9: 614–22. 10.1038/nchem.2712 28644469

[43] Hou Y , Yuan J, Zhou Y et al. A concise approach to site-specific topological protein-poly(amino acid) conjugates enabled by in-situ generated functionalities. J Am Chem Soc 2016; 138: 10995–1000. 10.1021/jacs.6b05413 27494383

[44] De P , Li M, Gondi SR et al. Temperature-regulated activity of responsive polymer-protein conjugates prepared by grafting-from via RAFT polymerization. J Am Chem Soc 2008; 130: 11288–9. 10.1021/ja804495v 18665597

[45] Bontempo D , Maynard HD. Streptavidin as a macroinitiator for polymerization: in situ protein-polymer conjugate formation. J Am Chem Soc 2005; 127: 6508–9. 10.1021/ja042230+ 15869252

[46] Boyer C , Bulmus V, Liu JQ et al. Well-defined protein-polymer conjugates via in situ RAFT polymerization. J Am Chem Soc 2007; 129: 7145–54. 10.1021/ja070956a 17500523

[47] Russell AJ , Baker SL, Colina CM et al. Next generation protein-polymer conjugates. AIChE J 2018; 64: 3230–45. 10.1002/aic.16338

[48] Qi YZ , Simakova A, Ganson NJ et al. A brush-polymer/exendin-4 conjugate reduces blood glucose levels for up to five days and eliminates poly(ethylene glycol) antigenicity. Nat Biomed Eng 2017; 1: 0002. 10.1038/s41551-016-0002 PMC5627778 28989813

[49] Isarov SA , Pokorski JK. Protein ROMP: aqueous graft-from ring-opening metathesis polymerization. ACS Macro Lett 2015; 4: 969–73. 10.1021/acsmacrolett.5b00497 35596466

[50] Lu J , Wang H, Tian Z et al. Cryopolymerization of 1,2-dithiolanes for the facile and reversible grafting-from synthesis of protein–polydisulfide conjugates. J Am Chem Soc 2020; 142: 1217–21. 10.1021/jacs.9b12937 31927989

[51] Wang H , Hu Y, Wang Y et al. Doxorubicin@ PEPylated interferon-polydisulfide: a multi-responsive nanoparticle for enhanced chemo–protein combination therapy. Giant 2021; 5: 100040. 10.1016/j.giant.2020.100040

[52] Tsuyuki H , Van Kley H, Stahmann MA. The preparation and physical properties of polypeptidyl proteins^1,2^. J Am Chem Soc 1956; 78: 764–7. 10.1021/ja01585a018

[53] Sela M , Arnon R. Studies on the chemical basis of the antigenicity of proteins. 3. Role of rigidity in the antigenicity of polypeptidyl gelatins. Biochem J 1960; 77: 394–9. 10.1042/bj0770394 13749856 PMC1204997

[54] Hu Y , Wang D, Wang H et al. An urchin-like helical polypeptide-asparaginase conjugate with mitigated immunogenicity. Biomaterials 2021; 268: 120606. 10.1016/j.biomaterials.2020.120606 33360506

[55] Yuan Z , Li B, Niu L et al. Zwitterionic peptide cloak mimics protein surfaces for protein protection. Angew Chem Int Ed 2020; 59: 22378–81. 10.1002/anie.202004995 32866343

[56] Liu M , Johansen P, Zabel F et al. Semi-permeable coatings fabricated from comb-polymers efficiently protect proteins in vivo. Nat Commun 2014; 5: 5526. 10.1038/ncomms6526 25407758

[57] Dong C , Wu G, Chen C et al. Site-specific conjugation of a selenopolypeptide to alpha-1-antitrypsin enhances oxidation resistance and pharmacological properties. Angew Chem Int Ed 2022; 61: e202115241. 10.1002/anie.202115241 34897938

[58] Wang H , Hou Y, Hu Y et al. Enzyme-activatable interferon–poly(α-amino acid) conjugates for tumor microenvironment potentiation. Biomacromolecules 2019; 20: 3000–8. 10.1021/acs.biomac.9b00560 31310511

[59] Hou Y , Zhou Y, Wang H et al. Therapeutic protein PEPylation: the helix of nonfouling synthetic polypeptides minimizes antidrug antibody generation. ACS Cent Sci 2019; 5: 229–36. 10.1021/acscentsci.8b00548 30834311 PMC6396190

[60] Hou Y , Lu H. Protein PEPylation: a new paradigm of protein–polymer conjugation. Bioconjugate Chem 2019; 30: 1604–16. 10.1021/acs.bioconjchem.9b00236 31045353

[61] Hou Y , Zhou Y, Wang H et al. Macrocyclization of interferon-poly(alpha-amino acid) conjugates significantly improves the tumor retention, penetration, and antitumor efficacy. J Am Chem Soc 2018; 140: 1170–8. 10.1021/jacs.7b13017 29262256

[62] Pelegri-O’Day EM , Lin EW, Maynard HD. Therapeutic protein-polymer conjugates: advancing beyond PEGylation. J Am Chem Soc 2014; 136: 14323–32. 10.1021/ja504390x 25216406

[63] Chen C , Wah Ng DY, Weil T. Polymer bioconjugates: modern design concepts toward precision hybrid materials. Prog Polym Sci 2020; 105: 101241. 10.1016/j.progpolymsci.2020.101241

[64] Zhang P , Sun F, Liu SJ et al. Anti-PEG antibodies in the clinic: current issues and beyond PEGylation. J Controlled Release 2016; 244: 184–93. 10.1016/j.jconrel.2016.06.040 PMC5747248 27369864

[65] Wurm F , Klos J, Rader HJ et al. Synthesis and noncovalent protein conjugation of linear-hyperbranched PEG-poly(glycerol) α,ω_n_-telechelics. J Am Chem Soc 2009; 131: 7954–5. 10.1021/ja9019148 19462953

