# Supplementary material for: Water-assisted and protein-initiated fast and controlled ring-opening polymerization of proline N-carboxyanhydride
Source: Natl Sci Rev. 2022 Feb 24;9(8):nwac033. doi: 10.1093/nsr/nwac033 (PMC9438472; doi:10.1093/nsr/nwac033)
Supplement: nwac033_Supplemental_File [file nwac033_supplemental_file.zip › Supporting_Information.docx]

Supporting Information

for

**Water-Assisted and Protein-Initiated Fast and Controlled Ring-Opening Polymerization of Proline *N*-Carboxyanhydride**

Yali Hu,^1,2^ Zi-You Tian,^1^ Wei Xiong, ^1^ Dedao Wang,^3^ Ruichi Zhao,^1^ Yan Xie,^3^ Yu-Qin Song,^3^ Jun Zhu,^3^ and Hua Lu^1,*^

^1^Beijing National Laboratory for Molecular Sciences, Center for Soft Matter Science and Engineering, Key Laboratory of Polymer Chemistry and Physics of Ministry of Education, College of Chemistry and Molecular Engineering, Peking University, Beijing 100871, People’s Republic of China.

^2^Peking-Tsinghua Center for Life Sciences, Academy for Advanced Interdisciplinary Studies, Peking University, Beijing 100871, People’s Republic of China.

^3^Key Laboratory of Carcinogenesis and Translational Research (Ministry of Education), Department of Lymphoma, Peking University Cancer Hospital & Institute, 52 Fucheng Road, Haidian District, Beijing 100142, People’s Republic of China.

Corresponding Author: [chemhualu@pku.edu.cn](mailto:chemhualu@pku.edu.cn) (H.L.)

**Contents**

**Experimental Section** S3

**Materials** S3

**Instruments** S3

**Cell Lines and Animals** S4

**Measurement of d*n*/d*c* and molecular weight of PLP** S4

**Synthesis of ProNCA** S5

**Typical procedure of the ROP of ProNCA in mixed ACN/H_2_O** S5

**Kinetic studies of the ROP of ProNCA in mixed ACN-*d*_3_/D_2_O** S5

**Kinetic studies of the ROP of ProNCA in ACN** S6

**DFT calculations** S6

**Synthesis of site-specific PLP-EGFP conjugate via NCL** S7

**Protein-initiated ROP of ProNCA (Synthesis of protein-PLP conjugate via grafting-from)** S7

**Cytotoxicity Assay** S8

**Pharmacokinetics Assay** S8

**In vivo antitumor efficacy and safety** S9

**Immunization and ELISA** S9

**Supplementary data** S12

**Data of DFT calculations** S38

**References** S68

**Experimental Section**

**Materials**

_L_-Boc-Proline and _D_-Boc-Proline were purchased from GL Biochem (Shanghai, China). Triphosgene was purchased from J&K (Beijing, China). ProNCA was obtained in accordance with a new NCA protocol recently developed by our laboratory.^1^ Benzylamine and diethylamine were purchased from Aladdin (Shanghai, China). *p*-Toluenethiol was purchased from J&K (Beijing, China). *p*-Toluidine was purchased from TCI (Tokyo, Japan). Glucosamine was purchased from Heowns (Tianjin, China). Dry acetonitrile (ACN) was obtained from a solvent purification system. Regular acetonitrile, pyridine, dimethyl formamide, tetrahydrofuran, dimethylsulfoxide, and ethyl acetate were purchased from Concord Technology (Tianjin, China). ACN-*d*_3_ was purchased from CIL (Cambridge, UK). Deuterium oxide (D_2_O) was purchased from Energy Chemical (Shanghai, China). Deuterium chloride, 20 wt. % solution in D_2_O, was purchased from J&K (Beijing, China). Dihydrofolate reductase (DHFR) from *E.coli.* was expressed and purified according to former method.^2^ Dihydrofolic acid (FAH_2_) was purchased from Sigma-Aldrich (St. Louis, USA). NADPH was purchased from Bioss (Beijing, China). Asparaginase (ASNase) from *E.coli.* was manufactured by Changzhou Qianhong Bio-pharma Co., Ltd (Jiangsu, China). CellTiter-Blue^®^ was purchased from Promega (Madison, USA). Methoxy PEG Maleimide (mPEG-MAL, *M*_n_ 5 kDa) was purchased from JenKem Technology Co., Ltd (Beijing, China). Endotoxin affinity beads, which was used to remove endotoxin from all protein variants for *in vivo* studies, were purchased from Senhui microsphere tech Co., Ltd (Suzhou, China). Nessler’s reagent was purchased from Merck (Darmstadt, Germany). Goat anti-rat IgM mu chain (HRP) and goat anti-rat IgG Fc (HRP) were purchased from Abcam (Cambridge, UK).

**Instruments**

NMR spectra were analyzed on ARX400 (Bruker, Germany). IR spectra were analyzed on Alpha II (Bruker, Germany) using a 0.1 mm path length super sealed KBr cell. Mass spectra of poly-_L_-proline (PLP) were analyzed on MALDI-TOF/TOF mass spectrometer 5800 (AB Sciex, USA). Circular dichroism spectroscopy was analyzed on a J-815 CD spectrometer (JASCO, Japan). Size exclusion chromatography (SEC) measurements were performed on two ÄKTA pure (GE, USA) equipped with different detectors. Separation was realized using a size exclusion Superdex75 10/300 GL column. Purification of protein-PLP conjugates was performed on ÄKTA pure (GE, USA) with an anion exchange mono Q column or a size exclusion Superdex200 increase 10/300 GL column. Protein concentration was determined by NanoDrop 2000 (Thermo Fisher, USA). SDS-PAGE gels were imaged on a Gel Doc XR^+^ (Bio-Rad, USA). Native PAGE gels were imaged on Typhoon FLA 9500 (GE, USA). Enzyme-linked immunosorbent assay (ELISA), cell viability assay, and the enzyme activity assay were recorded on a multimode plate reader (PerkinElmer, USA). The histologic sections were imaged on Axio Scan Z1 (Carl Zeiss, Germany).

**Cell Lines and Animals**

Human NK/T lymphoma NKYS cells were cultured in RPMI 1640 (Corning, USA) with 10% FBS (Gibco, USA), 100 U/mL penicillin (Corning, USA), 100 μg/mL streptomycin (Corning, USA), and 10 ng/mL IL2 (SL Pharm, China) at 37 °C with 5% CO_2_. SD rats were purchased from HFK Bioscience Co., Ltd (Beijing, China). B-NDG mice, i.e. NOD-*SCID* IL-2 receptor gamma null mice, were obtained from Biocytogen Co., Ltd (Suzhou, China). All the animal experiments were carried out with the permission of the experimental animal ethics committee in Beijing (project number: EAEC 2019-01).

**Measurement of d*n*/d*c* and molecular weight of PLP**

For regular analysis of PLP in the condition screening studies, the SEC equipped with a UV detector (λ set at 215 nm) was used to obtain the retention time information. **To obtain the absolute molecular weight (*M*_n_) and dispersity (*Đ*) of PLP**, the ÄKTA with a differential refractive index detector Optilab T-rEX (658.0 nm) and a light scattering detector miniDAWN TREOS (Wyatt, USA) was used.

Determination of d*n*/d*c*: a series of PLP solutions with gradient concentrations of 4.0, 2.0, 1.0, 0.5, 0.25, 0 mg/mL were prepared in advance and passed through a differential refractive index detector from low to high concentrations. The returned differential refractive index signal value corresponding to each concentration was used to calculate the d*n*/d*c* value of PLP.

The d*n*/d*c* of PLP in 1×PBS at 25 ℃ is determined as 0.1783 ± 0.3627% mL/g. The d*n*/d*c* of Poly-_D_-proline was assumed to be the same as PLP. The d*n*/d*c* of poly-_DL_-proline in 1×PBS at 25 ℃ was measured as 0.1745 ± 0.2672% mL/g.

**Synthesis of ProNCA**

*N*-Boc Proline (5.0 g, 23.2 mmol, 1.0 equiv) was added to 50 mL of acetonitrile and cooled in an ice-water bath. The solution was then sequentially added propylene oxide (13.5 g, 232 mmol, 10 equiv) and triphosgene (3.45 g, 11.6 mmol, 0.5 equiv). The mixture was stirred at 0 ℃ for 4 h before the addition of cooled water (15 mL), saturated brine (15 mL), and ethyl acetate (20 mL). After extraction, the aqueous phase was discarded. The organic phase was concentrated by rotary evaporation below 35 ℃ and the crude product was purified with silica column chromatography (V_petroleum ether_:V_ethyl acetate_ = 4:1). The product was concentrated under vacuum below 35 ℃ and further dried using an oil pump to obtain the product as a white solid (2.4 g, yield 73%). The ProNCA was transferred to a glove box and stored in a refrigerator at -30 °C.

Tips: A recrystallization of commercial *N*-Boc Proline was recommended to improve the yield. The phosgenation reaction can be done either in an inert or ambient atmosphere. Silica gel for column chromatography was oven-dried at 96 ℃ for 4 h before use.

**Typical procedure of the ROP of ProNCA in mixed ACN/H_2_O**

In a cold room under 10 ℃, ProNCA (100 mg, 0.71 mmol, 100 equiv) was dissolved in ACN (500 μL) and was added H_2_O (500 μL). The clear solution was added benzylamine as initiator (14 μL × 0.5 M, 7.1 μmol, 1.0 equiv) under stirring, with immediate gas bubbling observed. No precipitation was observed during the course (see supporting video). The clear solution was typically quenched after 5 min reaction, diluted with 4 mL ddH_2_O, purified using a PD-10 desalting column (GE, USA), and freeze-dried to obtain a white fluffy solid (61 mg, yield 88%). PLPs of different *M*_n_ were prepared in a similar method, with a slight change on the initial monomer concentrations.

**Kinetic studies of the ROP of ProNCA in mixed ACN-*d*_3_/D_2_O**

The high water content of the system prevents regular FT-IR spectroscopy. It should also be point out that in-situ FT-IR and regular ^1^H NMR spectroscopy both failed simply because the time intervals required for each data point acquisition was at least 30 s and therefore not suitable for the ROPs that finished in seconds or less than 5 min. We have previously shown that HCl could hydrolyze NCA without initiate polymerization.^1^ Therefore, a parallel DCl-quenching protocol was developed for this particular polymerization.

Taking the reaction at a [ProNCA]_0_/[I] of 100/1 as an example: A flat-bottom 96-well plate was placed on a magnetic stirrer. 9 wells were each added a tiny stir bar, ProNCA (14 mg, 99.2 μmol, 100 equiv) in 70 μL acetonitrile-*d*_3_ (ACN-*d*_3_)_,_ and 70 μL D_2_O containing 0.4% DMSO (as an internal standard for ^1^H NMR analysis). To the wells was quickly added benzyl amine (10 μL × 0.1 M, 1.0 equiv) using a multi-channel pipette under stirring to initiate the ROP simultaneously (time 0 s). The reactions were quenched by adding DCl/D_2_O (20 wt.% 20 μL) at designated time points (10, 20, 30, 40, 50, 60, 80, 120, and 160 s, respectively). The addition of DCl rapidly hydrolyzed all unreacted ProNCA into free _L_-proline. Finally, each reaction was diluted with D_2_O to a total volume of 500 μL for ^1^H NMR tests. The conversions of ProNCA were calculated based on the following formula:

Conv. = *I*_3.8 ppm_/ (*I*_4.5 ppm_ *+ I*_3.8 ppm_)

where *I*_3.8 ppm_ and *I*_4.5 ppm_ were the integration of the peak at 3.8 (PLP) and 4.5 (Pro) ppm, respectively (see Figure S9).

The kinetic studies at the [ProNCA]_0_/[I] of 50/1 (Figure S8) and 200/1 (Figure S10) were conducted in similar manners.

**Kinetic studies of the ROP of ProNCA in ACN**

To seven 5-mL glass vials in a glovebox was each added ProNCA (100 mg, 0.71 mmol, 100 equiv) and 1.0 mL ACN. The solutions were each added benzyl amine (14 μL × 0.5 M, 7.1 μmol, 1.0 equiv) to initiate the ROP (time 0). At each designated time point (0, 0.5, 2, 4, 8, 24, 48, and 168 h), an aliquot of the milky suspension was taken out from one of the seven vials, quickly centrifuged (21,000 g × 5 min), and the supernatant was analyzed by FT-IR using a KBr cell with fixed a path length. Quantification was based on the absorption intensity at 1853 cm^-1^, the characteristic anhydride peak of ProNCA. A standard working curve was drawn using ProNCA solutions at various known concentrations.

**DFT calculations**

All DFT calculations were performed in Gaussian 09 E program.^3^ Becke3LYP functional^4-5^ was used to locate all the stationary points in the acetonitrile solution phase with SMD solvation model.^6^ The Pople 6-31G(d,p)^7-8^ basis set was applied for all elements. The frequency analysis calculations were also preformed to confirm whether the stationary points were transition states or intermediates. Intrinsic reaction coordinate (IRC) calculations were executed to relate corresponding intermediates. The reported activation of Gibbs free energies included the thermal corrections computed at 298 K and are corrected at the standard concentration (1 mol/L).

**Synthesis of site-specific PLP-EGFP conjugate via NCL**

Tev-EGFP was expressed in *E.coli.*, ^9^ Cys-EGFP was obtained by tev digestion from tev-EGFP using a previously reported method.^10^ Cys-EGFP (10 mg, 0.35 μmol, 1.0 equiv) in PBS was added PMT-PLP (*M*_n_ = 10 kDa, 17.5 mg, 1.75 μmol, 5.0 equiv) and incubated at 4 ℃ overnight. Purification of the conjugate was performed on a ÄKTA pure (GE, USA) using an anion exchange Mono Q 5/50 GL column (binding buffer: 50 mM Tris-HCl, pH 8.0; elution buffer: 50 mM Tris-HCl, 2 M NaCl, pH 8.0). The PLP-EGFP conjugate was characterized by both SDS-PAGE and Native PAGE, yield 73%.

**Protein-initiated ROP of ProNCA (Synthesis of protein-PLP conjugate via grafting-from)**

**EGFP**: In a cold room at 10 ℃, ProNCA was dissolved in ACN at 50, 25, 12.5, 5.0, 2.5 mg/mL, or 1.0 mg/mL, respectively. The ProNCA solutions were each mixed with isometric EGFP (5.0 mg/mL) in PBS and incubated for 10 min. The crude reaction solutions were characterized with both SDS-PAGE and native PAGE without purification.

**DHFR**: In a cold room at 10 ℃, ProNCA was dissolved in ACN at 40, 20, 10, or 5.0 mg/mL, respectively. The ProNCA solutions were each mixed with isometric DHFR (4.6 mg/mL) in PBS and incubated for 10 min. The conjugates were purified by passing through a Superdex 200 increase 10/300 GL column for buffer exchange and then characterized with SDS-PAGE.

**DHFR activity Assay**

The assay was carried out by following manufacturer’s instructions. Briefly, the substrates NADPH and FAH_2_ were dissolved in phosphate buffer (50 mM, pH 7.5) containing 5 mM β-mercaptoethanol to make a final solution of 0.25 mM NADPH and 0.25 mM FAH_2_. The substrate solution (140 μL) was mixed with wild-type DHFR or the DHFR-PLP conjugates (10 μL × 1.1 μM DHFR) and incubated at 25 ℃ for 15 min. The reactions were monitored every 30 s by measuring the absorption at 340 nm.

Tolerance of high temperature: wild-type DHFR and the DHFR-PLP conjugates (50 μL × 11 μM DHFR) were incubated at 80 ℃ for 10 min and then diluted into a final DHFR concentration of 1.1 μM for the subsequent activity assay at room temperature.

Tolerance of organic solvent: wild-type DHFR and the DHFR-PLP conjugates (50 μL × 11 μM DHFR) were each mixed with isometric ethanol and incubated at room temperature for 12 h. The ethanol-treated DHFR solutions were then diluted into a final DHFR concentration of 1.1 μM for the subsequent activity assay.

**ASNase**: In a cold room at 10 ℃, ProNCA was dissolved in ACN at 20 mg/mL. The ProNCA solutions were each mixed with isometric ASNase (8 mg/mL) in PBS and incubated for 10 min. The conjugates were purified by passing through a Superdex 200 increase 10/300 GL column for buffer exchange and then characterized with SDS-PAGE. The afforded pure conjugate was stored in PBS buffer (pH 7.4) at 4 ℃ and lipopolysaccharide (LPS) was removed before *in vivo* injection by using endotoxin affinity beads (Senhui Microsphere Tech Co., Ltd, China).

Synthesis of PEG-ASNase, cytotoxicity assay, pharmacokinetics assay, *in vivo* antitumor efficacy and safety, and immunization and ELISA were similar to established protocols. ^11^

**Cytotoxicity Assay**

NKYS cells were seeded at a density of 5,000 cells per well 24 h before treatment and incubated with gradient concentrations of ASNase variants for 48 h in a black 96-well plate (*n* = 3). The relative cell viability of each group was determined by CellTiter-Blue^®^ (Promega, USA) following manufacturer’s protocol. The data fitting and IC_50_ calculation were analyzed by GraphPad Prism 5.0 software.

**Pharmacokinetics Assay**

Female SD rats weighing ∼200 g were randomized into two groups (*n* = 4) and intravenously injected with wt ASNase or ASNase-PLP at a dosage of 40 U/rat (200 U/kg). At predetermined time points, the blood samples were acquired from orbit, standing for 30 min at 4 °C, and centrifuged at 4,500 g for 15 min. The sera were collected and stored at -80 °C before enzyme activity assay. The enzyme activity was measured by detecting the produced ammonium using Nessler’s reagent (Merck, Germany). Briefly, in a transparent 96-well plate, phosphate buffer (90 μL, pH 7.4), sample solution (10 μL), and serum (20 μL) were mixed and reacted at 37 ^o^C for 30 min. The reaction was terminated by trichloroacetic acid solution (TCA, 20 μL, 1.5 M) and centrifuged at 1,000 g for 5 min. The supernatant (20 μL) was added to another transparent 96-well plate containing Nessler’s reagent (20 μL, Merck, Germany) in phosphate buffer (160 μL, pH 7.4). Absorption at 410 nm were then measured by using a multimode plate reader (PerkinElmer, USA). Concentration was calculated based on a standard working curve containing ammonium with known concentrations. One unit of ASNase is defined as the amount of enzyme required to generate 1.0 µmole ammonia per minute at pH 7.4 and 37 °C.

**In vivo antitumor efficacy and safety**

NKYS cells (6.0 × 10^6^) suspended in 0.1 mL PBS were mixed with isometric Matrigel^TM^ matrix (Corning, USA) and subcutaneously inoculated into 6-week-old B-NDG mice. When the tumors grew to ∼300 mm^3^, the mice were randomized into three groups (*n* = 8) and received PBS, wt ASNase, or ASNase-PLP treatment at a dosage of 15 U/mouse via intraperitoneal injection every two weeks. The volume of tumor was acquired through the formula *V* = *L*×*W*^2^/2, where *V, L,* and *W* is the volume, length, and width of the tumor, respectively. The tumor volume and body weight were measured every 3 days.

Histopathology Evaluation: On day 38, the mice were sacrificed to extract tumors and major organs such as heart, liver, spleen, lung, and kidney. The extracted tumors sections were detected by immunohistochemical staining using Anti-Human Ki-67 on Axio Scan Z1 (Carl Zeiss, Germany) and the other organs slices were stained with hematoxylin and eosin (H&E).

**Immunization and ELISA**

Male and female SD rats were randomly grouped (*n* = 4) and received wt ASNase, ASNase-PLP, or PEG-ASNase subcutaneously at a weekly 200 U ASNase/kg dose. Blood were drawn before the immunization for benchmark (day 0), and before each injection (day 7, 14, 28, and 35). The anti-ASNase and antipolymer antibodies in the antisera were then evaluated by ELISA.

The antigens used in direct ELISAs: wt ASNase (for the detection of anti-ASNase IgG), PLP-interferon (IFN) conjugate (for the detection of anti-PLP antibody), and PEG-IFN conjugate (for the detection of anti-PEG antibody). The polypeptide-IFN^12^ and PEG-IFN^13^ conjugates were made by following the published protocols.

ELISA procedure for antipolymer IgM measurement: The Polymer (PLP or PEG)-IFN conjugate in PBS (100 μL × 1.0 μg/mL/well) was added to high-binding transparent 96-well plates (Corning, USA) and incubated at 4 °C overnight for antigen coating. The plate was washed with Wash Buffer (0.5‰ CHAPS in PBS, 200 μL/well × 3), and blocked with Assay Buffer (5% BSA in wash buffer, 100 μL) at room temperature for 2 h before adding the sample solutions. Next, prediluted antisera (100 μL, 200-fold dilution with the Assay Buffer) were added to the washed plate and incubated at room temperature for 1 h. Subsequently, the plate was washed for three times using Wash Buffer, and incubated with the secondary antibody goat anti-rat IgM mu chain HRP (100 μL, 5000-fold dilution with Assay Buffer) at room temperature for 1 h. Finally, after washing for four times, the plate was incubated with TMB solution (100 μL, CWBio) at room temperature for the chromogenic reaction. The reaction was terminated 5 min later with 2 N H_2_SO_4_ (100 μL) before reading the absorption at 450 nm in a plate reader.

ELISA procedure for anti-ASNase IgG and antipolymer IgG: the procedure was similarly carried out as mentioned above but using different coating antigens, dilution fold, and buffer recipe. More detailed information were available in Table S1.

ELISA procedure for anti-ASNase IgG titer: the procedure was similarly carried out as mentioned above but adding gradient dilution folds of antisera in week4.

**Table S1.** ELISA conditions and buffer recipe.

|  | Coating antigen | Wash Buffer | Assay Buffer | antisera dilution fold | Secondary antibody |
| --- | --- | --- | --- | --- | --- |
| Anti-ASNase IgG | 100 ng/well wt ASNase in PBS | 0.5‰ Tween-20 in PBS | 5% BSA and 0.5‰ Tween-20 in PBS | 5000 | Goat anti-rat IgG Fc HRP (ab97090) |
| Anti-polymer IgG | 100 ng/well polymer-IFN in PBS | 0.5‰ CHAPS in PBS | 5% BSA and 0.5‰ CHAPS in PBS | 500 | Goat anti-rat IgG Fc HRP (ab97090) |
| Anti-polymer IgM |  |  |  | 200 | Goat anti-rat IgM mu chain HRP (ab98373) |

**Supplementary data**

**
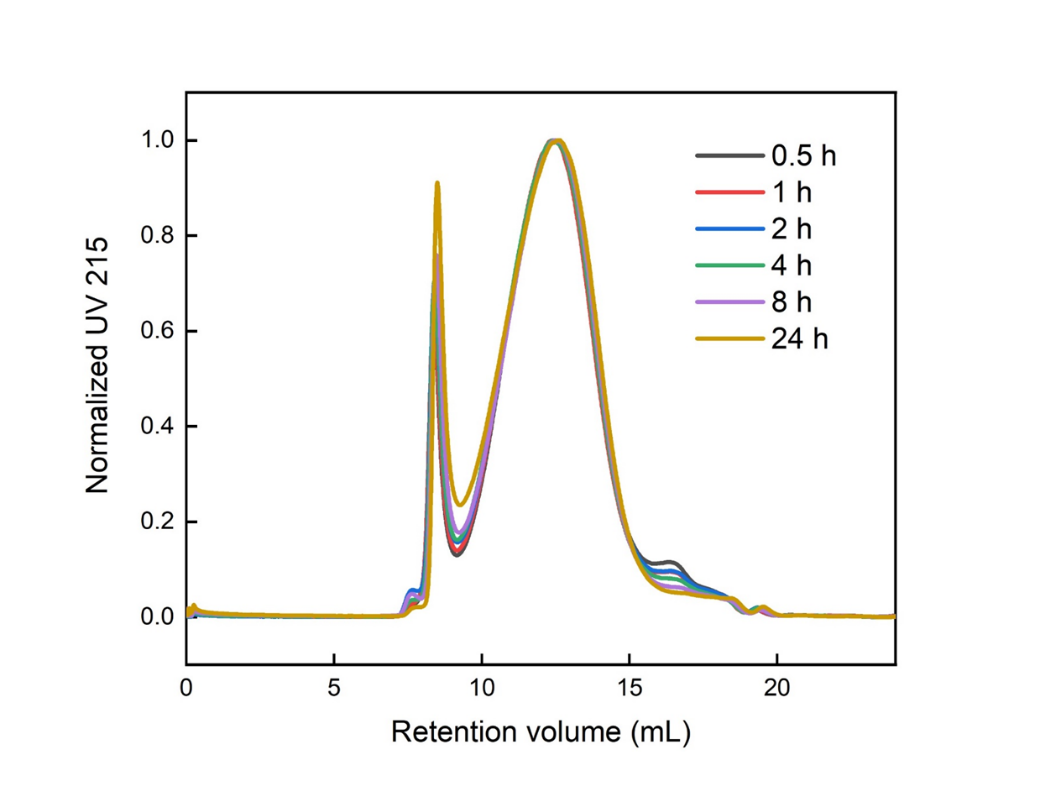
**

**Figure S1.** SEC analysis of PLPs obtained from the ROP of ProNCA in dry ACN at different reaction time points.

**Discussion:** SEC analysis showed bimodal peaks with no significant increase in *M*_n_ after 30 min of polymerization.

**
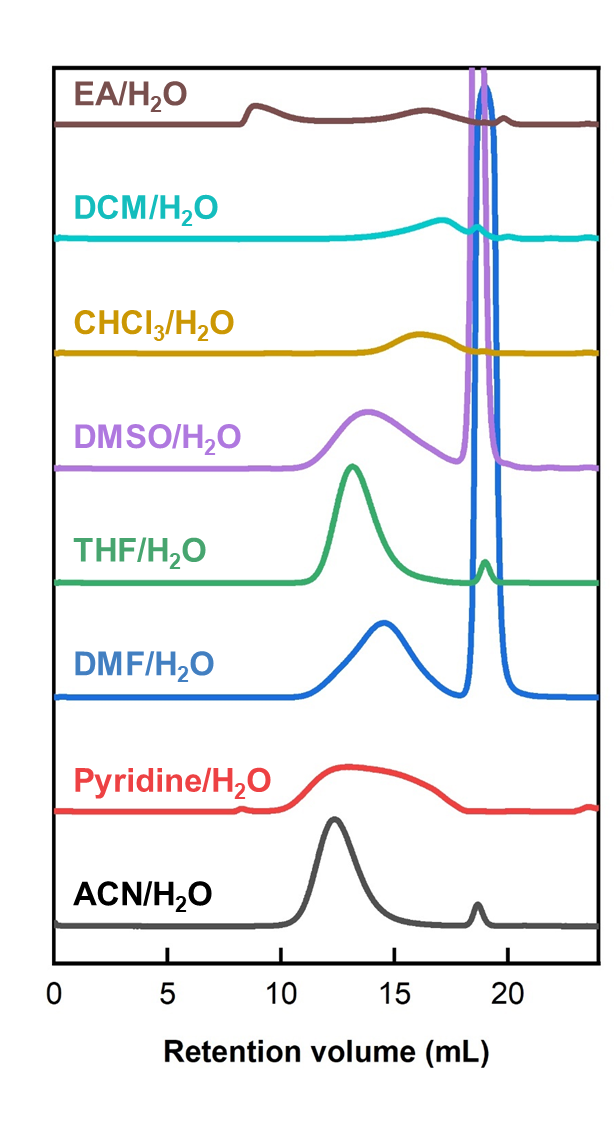
**

**
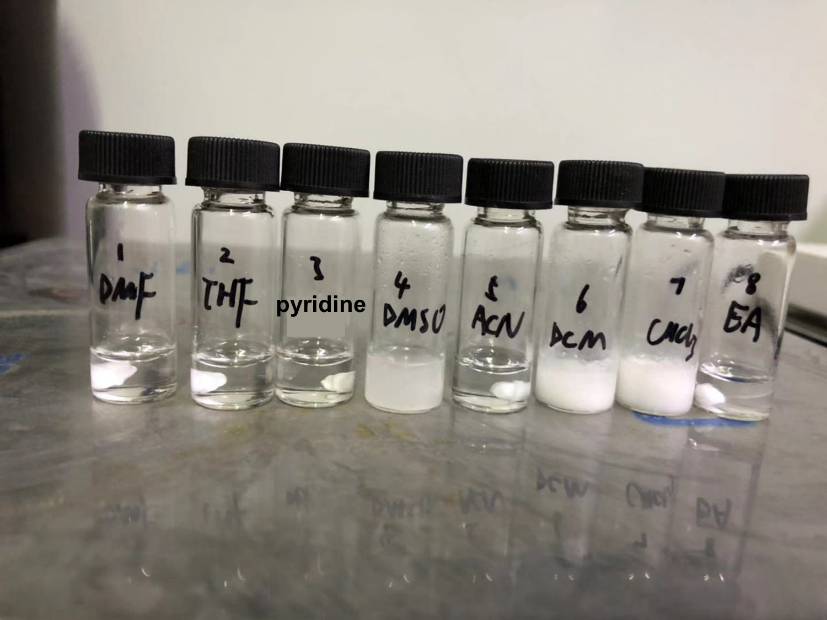
**

**Figure S2.** SEC traces (top) and a snapshot (bottom) of the ROP of ProNCA in different mixed organic solvent-H_2_O combinations.

**Discussion:** All reactions were conducted at the same temperature (10 ℃), the same monomer concentration (50 mg/mL), the same solvent ratio (H_2_O/organic solvent = 1/1 (v/v)), the same monomer/initiator ratio ([ProNCA]_0_/[BnNH_2_] = 50). Note: The peak near 19 min was due to organic solvents with absorption at 215 nm. Both ACN/H_2_O and THF/H_2_O showed high reaction rate, high *M*_n_, and narrow *Ð*. The ROP in DMF also gave a unimodal peak, but with slightly smaller *M*_n_. Reactions in mixed DMSO/ H_2_O, DCM/H_2_O, and CHCl_3_/H_2_O were heterogeneous.


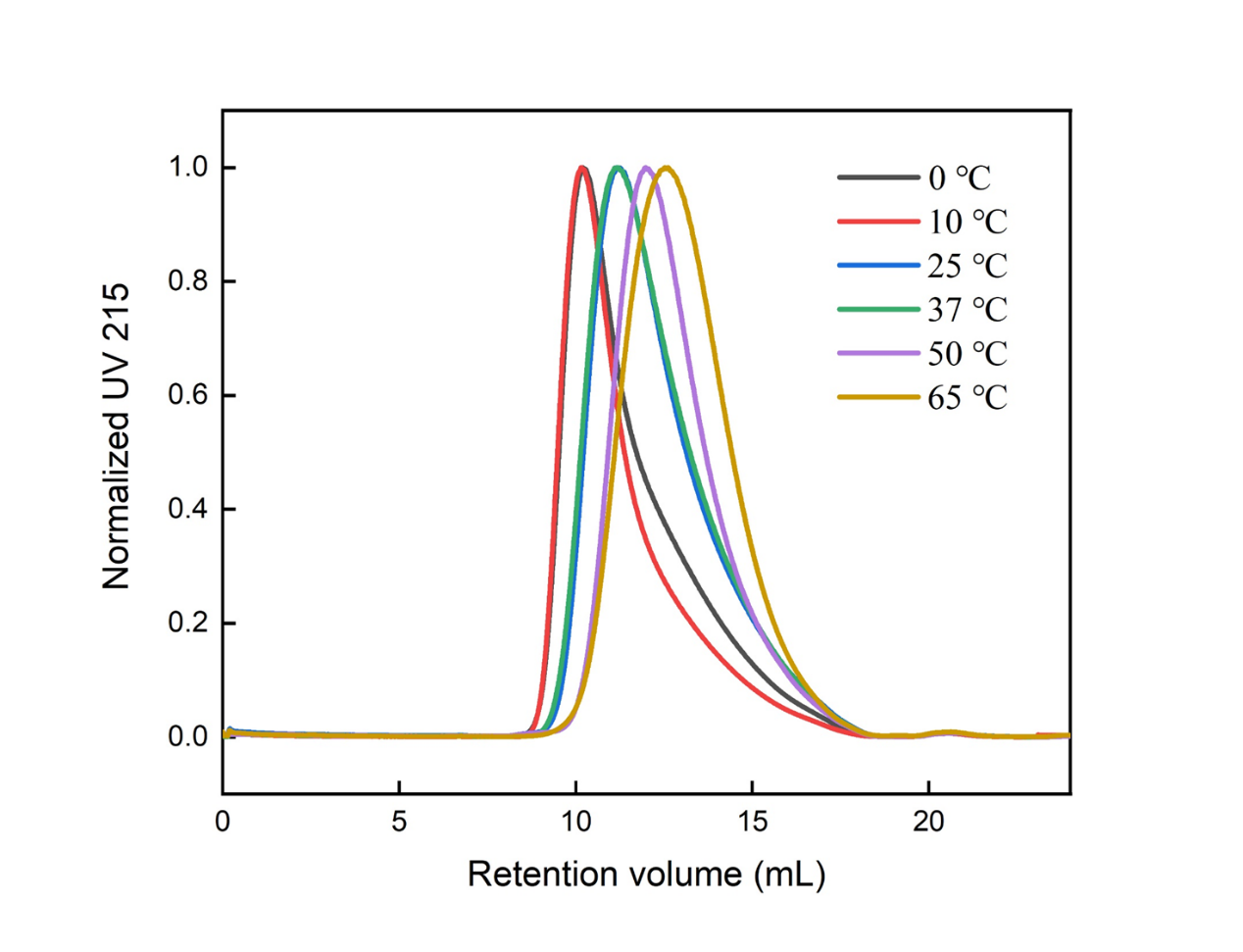


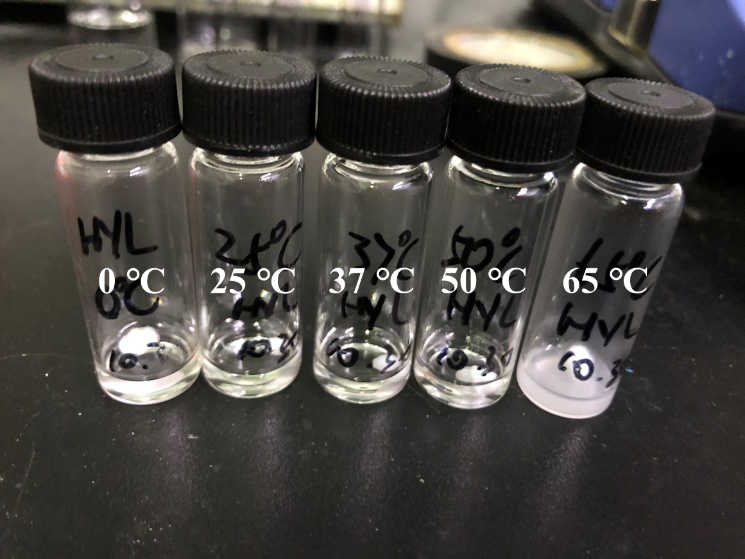


**Figure S3.** SEC traces (top) and a snapshot (bottom) of the ROP of ProNCA in mixed ACN/H_2_O at different temperatures.

**Discussion:** All reactions were conducted at the same monomer concentration (50 mg/mL), the same solvent ratio (H_2_O/ACN = 1/1 (v/v)), and the same monomer/initiator ratio ([ProNCA]_0_/[BnNH_2_] = 200). It appeared that unimodal SEC traces could be obtained at all tested temperatures in the range of 0 to 65 ^o^C, with an inverse correlation between *M*_n_ and temperature. We tentatively believe that this is due to the accelerated ProNCA hydrolysis at increased temperatures. Note that at 65 ^o^C, the reaction mixture became cloudy likely due to the lower critical solution temperature (LCST) of PLP.


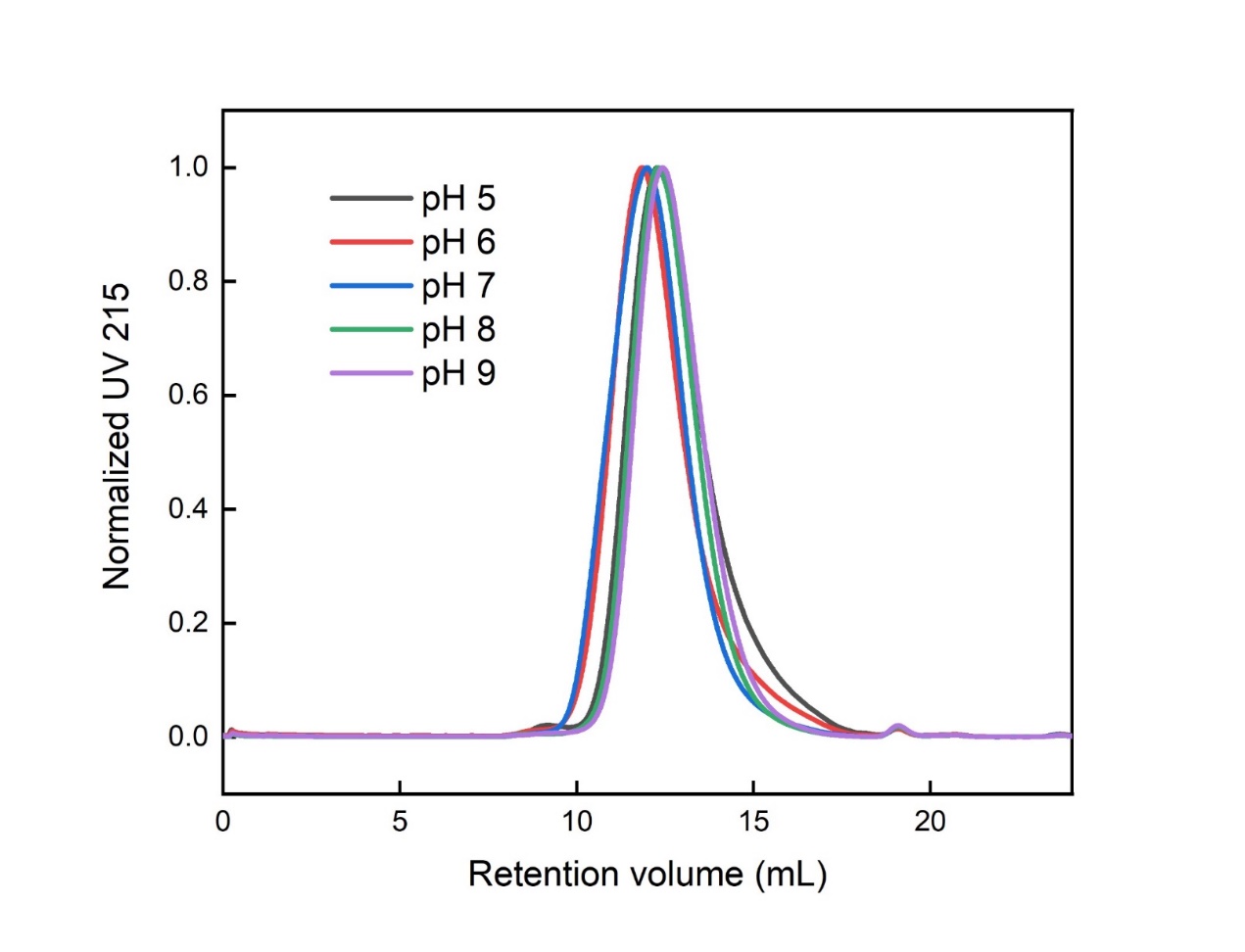


**Figure S4.** SEC traces of the ROP of ProNCA in mixed ACN/H_2_O at different pH.

**Discussion:** All reactions were conducted at the same temperature (10 ℃), the same monomer concentration (50 mg/mL), the same solvent ratio (H_2_O/ACN = 1/1 (v/v)), and the same monomer/initiator ratio ([ProNCA]_0_/[BnNH_2_] = 100). It appeared that pH variation between 5.0 and 9.0 had negligible effect on the *M*_n_, though the ROP tended to be slightly faster in a more alkaline environment.


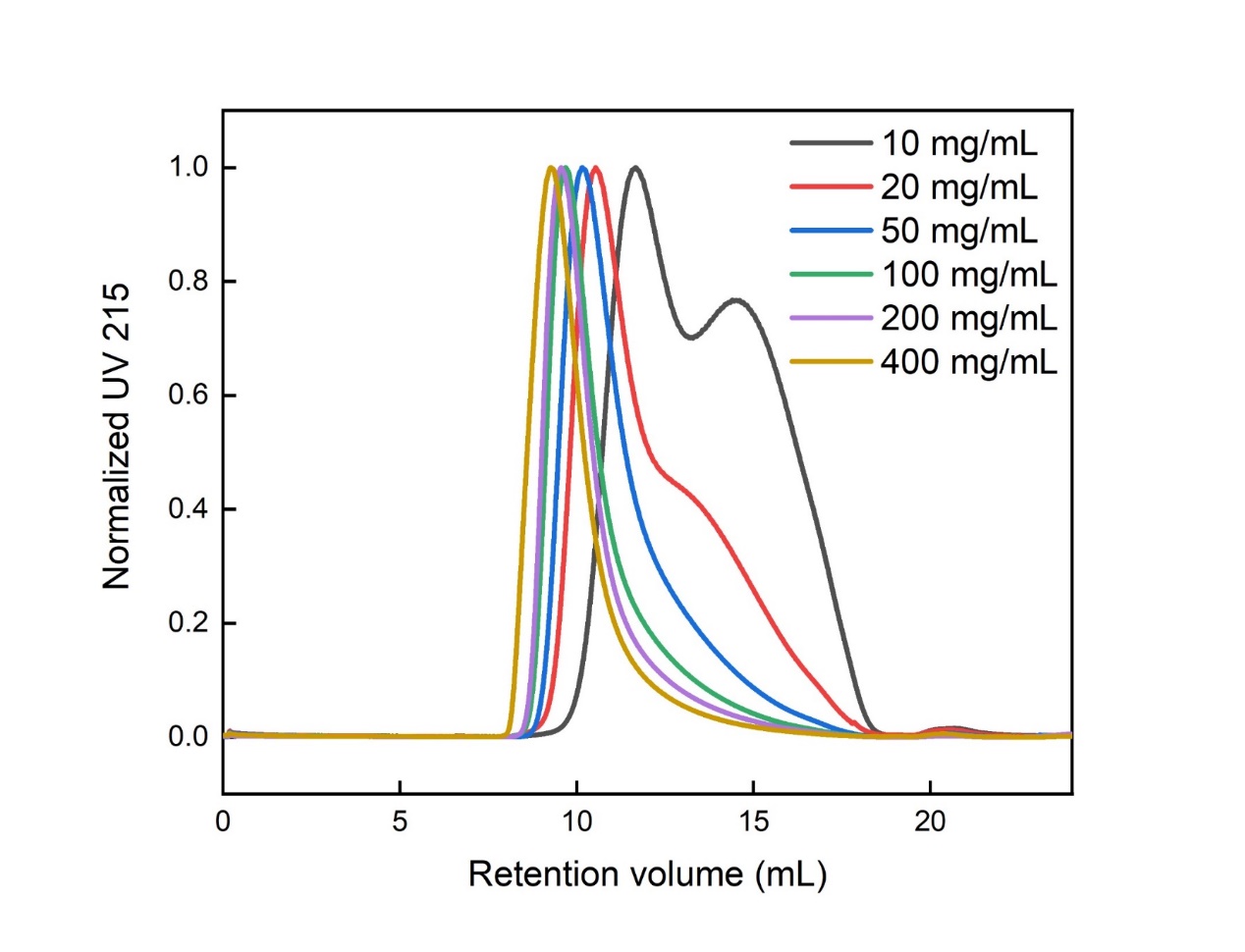


**Figure S5.** SEC traces of the ROP of ProNCA in mixed ACN/H_2_O at different initial monomer concentrations ([ProNCA]_0_).

**Discussion:** All reactions were conducted at the same temperature (10 ℃), the same solvent ratio (H_2_O/ACN = 1/1 (v/v)), and the same monomer/initiator ratio ([ProNCA]_0_/[BnNH_2_] = 200). With the [ProNCA]_0_ below 50 mg/mL, the ROP gave bimodal peaks and lower *M*_n_.


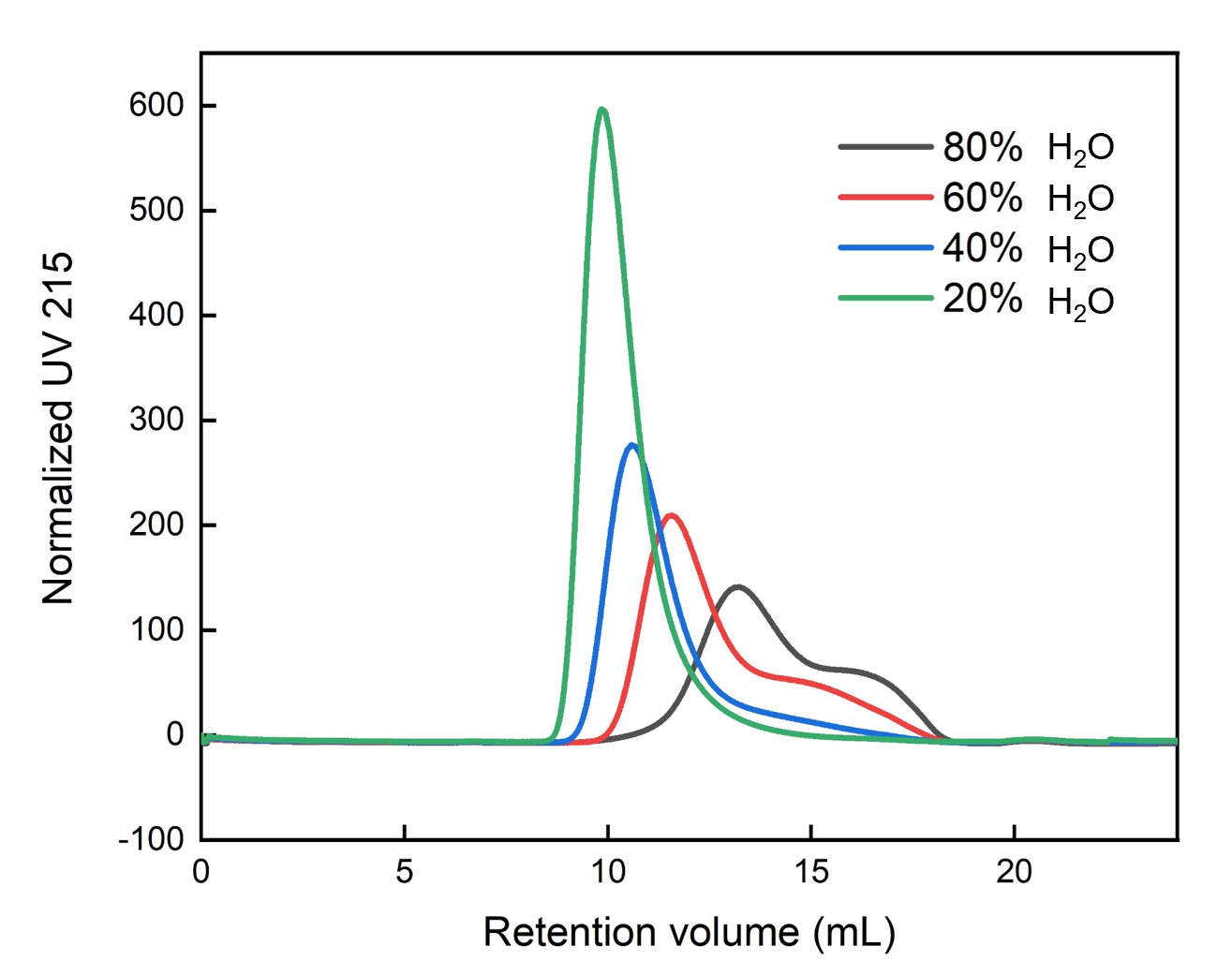


**
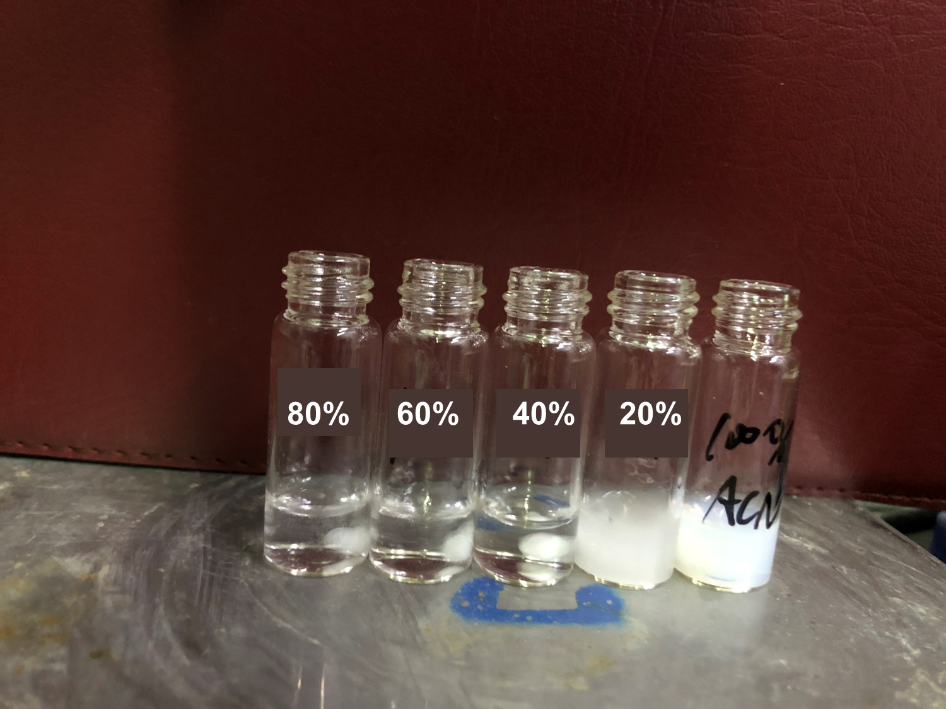
**

**Figure S6.** SEC traces (top) and a snapshot (bottom) of the ROP of ProNCA in mixed ACN/H_2_O at different percentage of water content.

**Discussion:** All reactions were conducted at the same temperature (10 ℃), the same monomer concentration (50 mg/mL), and the same monomer/initiator ratio ([ProNCA]_0_/[BnNH_2_] = 100). It appeared that a 20% water is effective enough to accelerate the ROP and gave PLP with a high *M*_n_ and unimodal SEC peak. It should be pointed out that, however, the reaction was a heterogeneous suspension rather than a clear solution at this ratio. With a water content higher than 60%, small shoulder peaks at the lower *M*_n_ region gradually grew. Thus, it was recommended to conduct the ROP at a 40-60% water content.


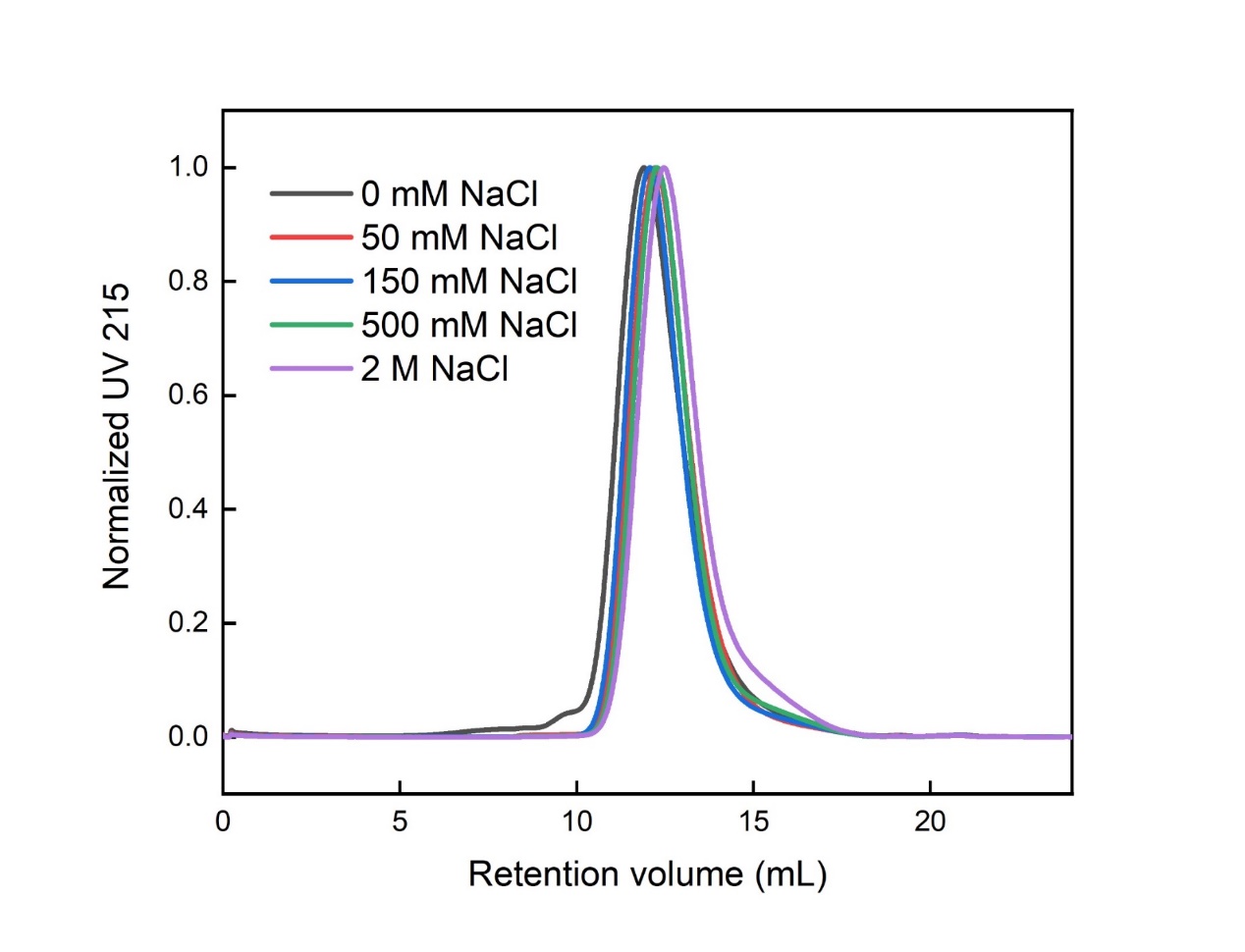


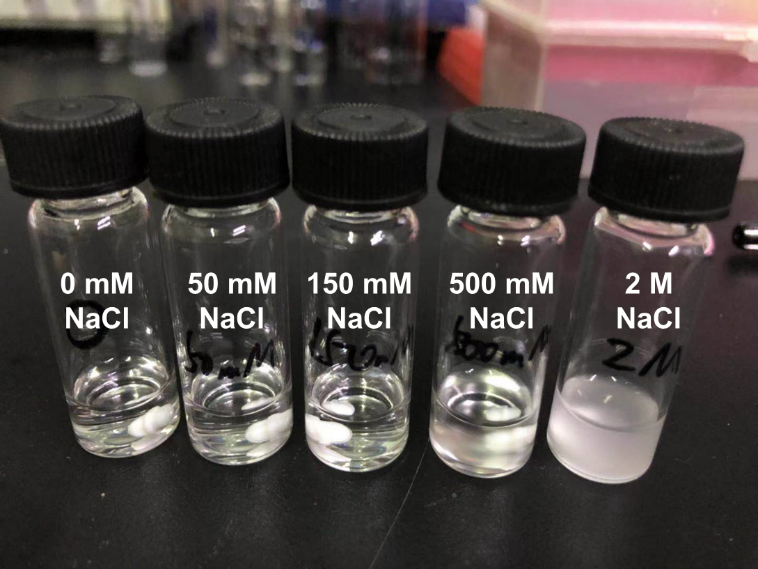


**Figure S7.** SEC traces (top) and a snapshot (bottom) of the ROP of ProNCA in mixed ACN/H_2_O at different concentrations of NaCl.

**Discussion:** All reactions were conducted at the same control temperature (10 ℃), the same monomer concentration (100 mg/mL), the same solvent ratio (H_2_O/ACN = 1/1 (v/v)), and the same monomer/initiator ratio ([ProNCA]_0_/[BnNH_2_] = 100). No significant difference was observed for all conditions tested. However, it should be pointed out that the solution became cloudy when [NaCl] reached 2.0 M.


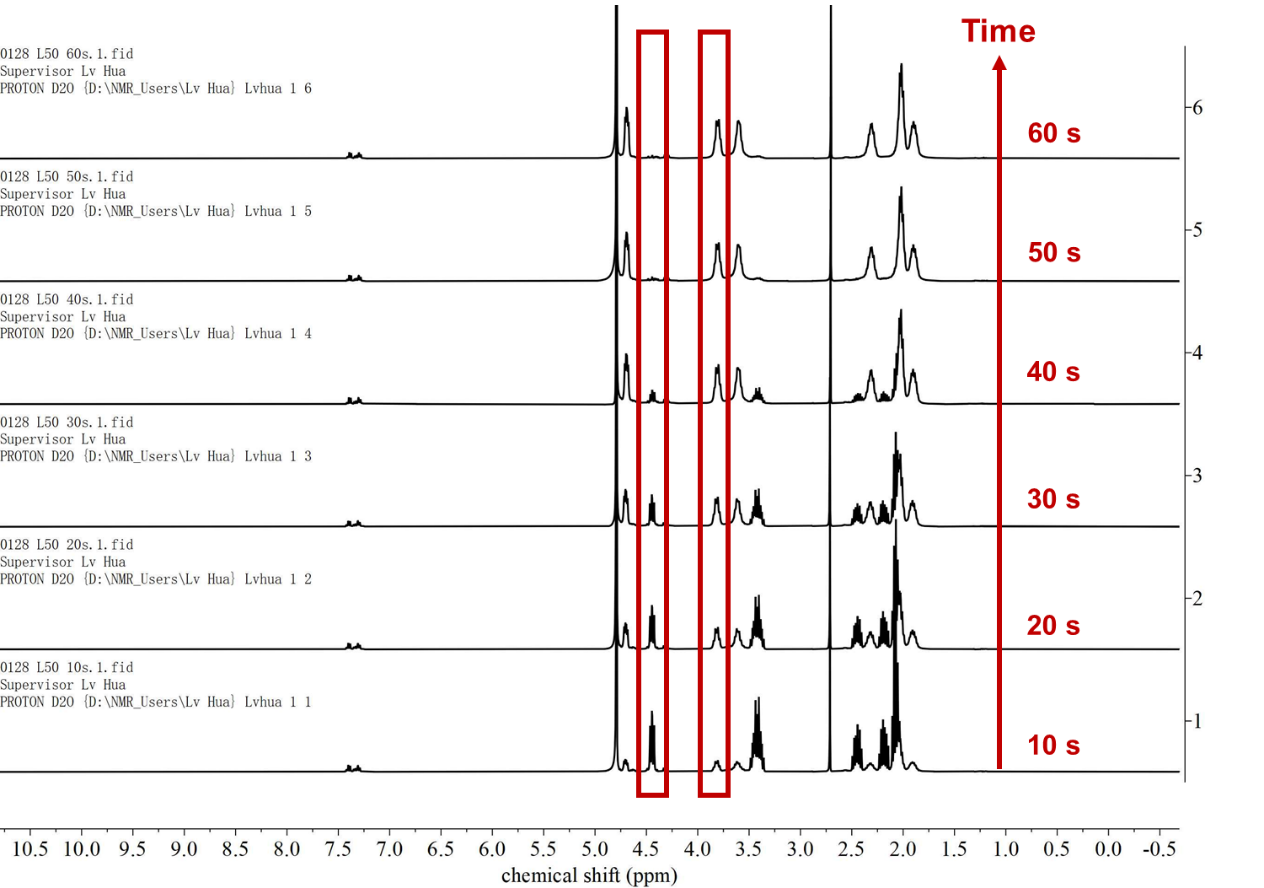


**Figure S8.** Overlay of the original ^1^H NMR spectra for the kinetic studies of the BnNH_2_-mediated ROP of ProNCA in mixed ACN-*d*_3_/D_2_O at a [PrNCA]_0_/[I] ratio of 50/1. The spectra were taken from ROP reactions quenched at specific time points. Note that the peaks in red box (4.5 ppm) were the hydrolyzed Proline rather than ProNCA.

**
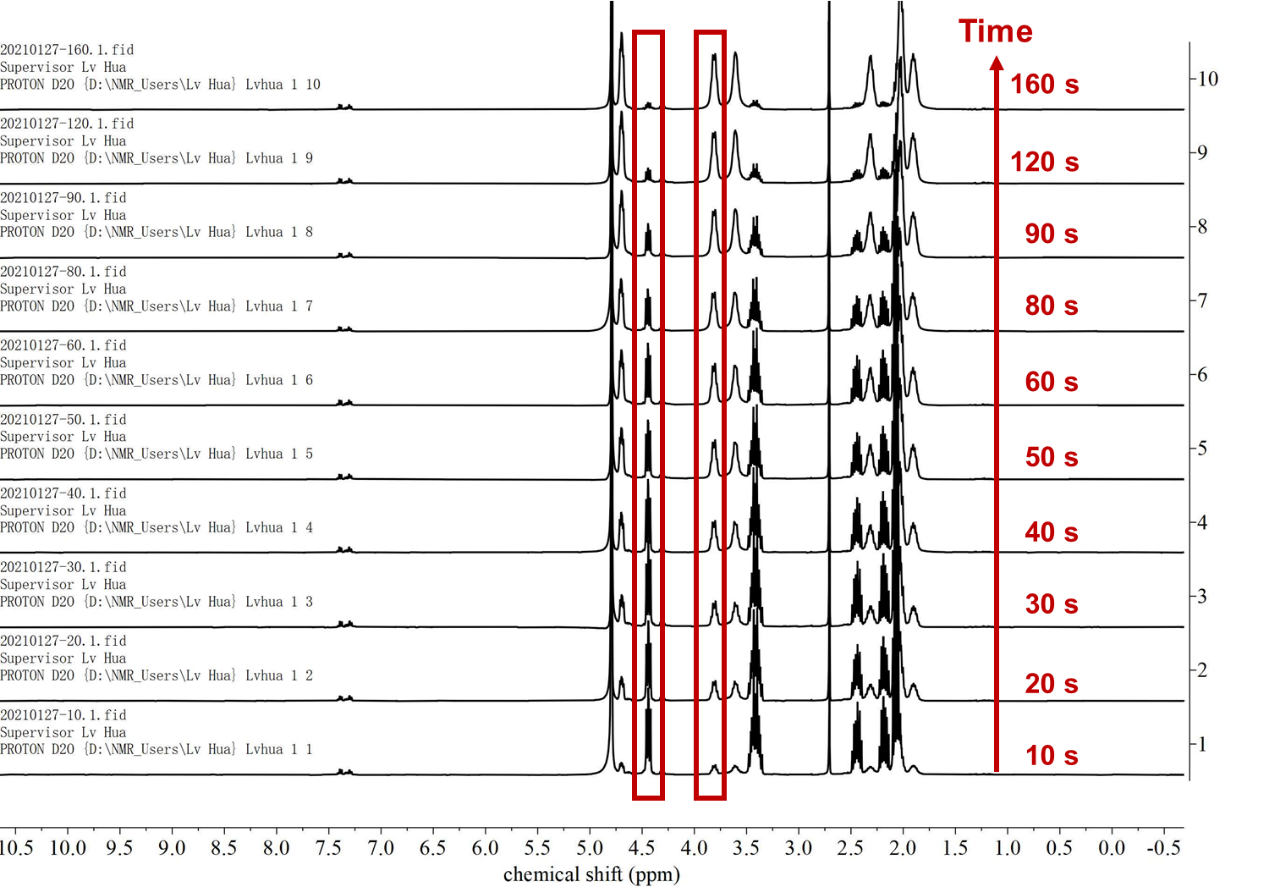
**

**Figure S9.** Overlay of the original ^1^H NMR spectra for the kinetic studies of the BnNH_2_-mediated ROP of ProNCA in mixed ACN-*d*_3_/D_2_O at a [PrNCA]_0_/[I] ratio of 100/1. The spectra were taken from ROP reactions quenched at specific time points. Note that the peaks in red box (4.5 ppm) were the hydrolyzed Proline rather than ProNCA.

**
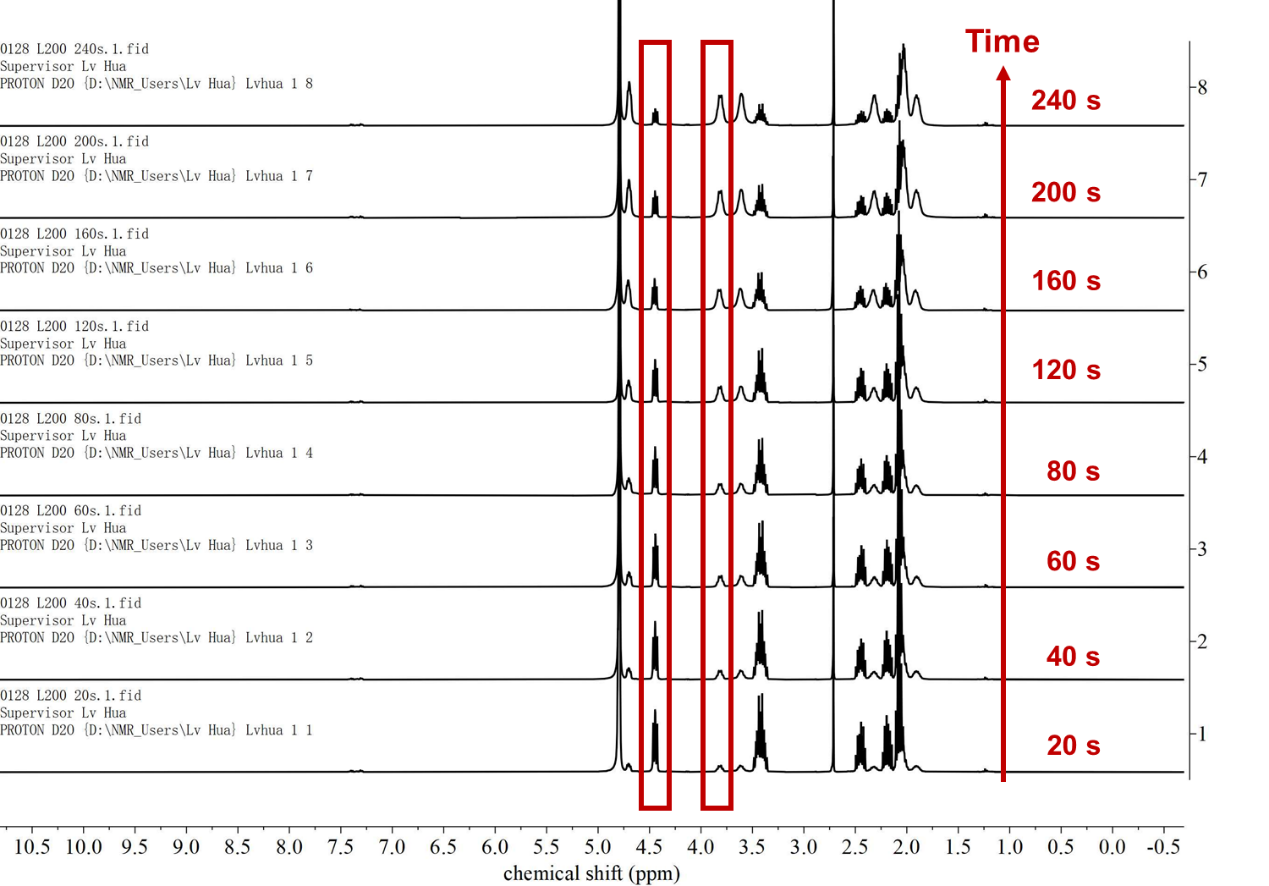
**

**Figure S10.** Overlay of the original ^1^H NMR spectra for the kinetic studies of the BnNH_2_-mediated ROP of ProNCA in mixed ACN-*d*_3_/D_2_O at a [PrNCA]_0_/[I] ratio of 200/1. The spectra were taken from ROP reactions quenched at specific time points. Note that the peaks in red box (4.5 ppm) were the hydrolyzed Proline rather than ProNCA.


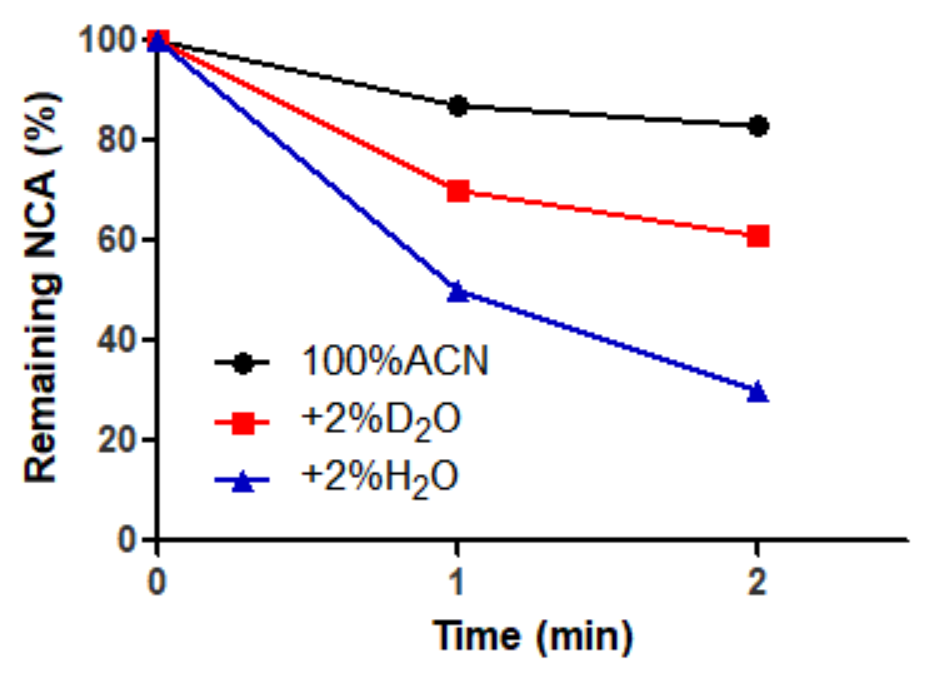


**Figure S11.** Accelerated ROP of ProNCA when 2% (v/v) D_2_O (red) or H_2_O (blue) was added to ACN.

**Discussion:** In this experiment, all reaction mixtures became more or less turbid due to the low solubility of PLP in ACN. To obtain accurate conversion of ProNCA, at each time point (1 or 2 min), an aliquot of the reaction was centrifuged (21,000 g × 2 min) and the supernatant was measured via FT-IR using a KBr cell with fixed volume and path length. The remaining NCA concentration was quantified based on the peak intensity at 1853 cm^-1^. Notably, the supernatant after centrifugation remained clear without precipitation or change of NCA concentration for more than one hour, indicating the centrifugation process temporarily quenched the reaction through physical separation of the reactive PLP (as a precipitate) from the monomer solution.


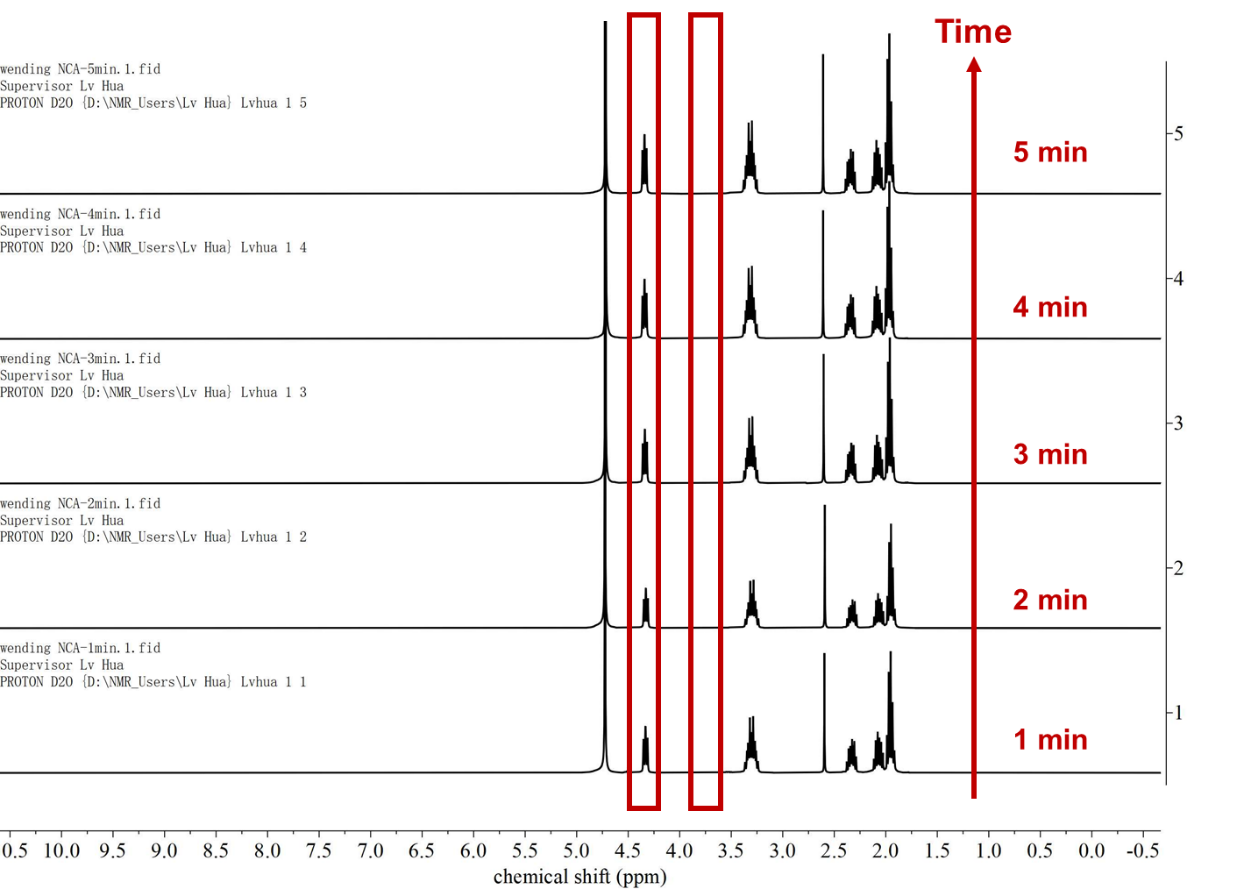


**Figure S12.** ProNCA monomer remained intact for 5 min under the ROP condition without an amine initiator at 4 ^o^C.


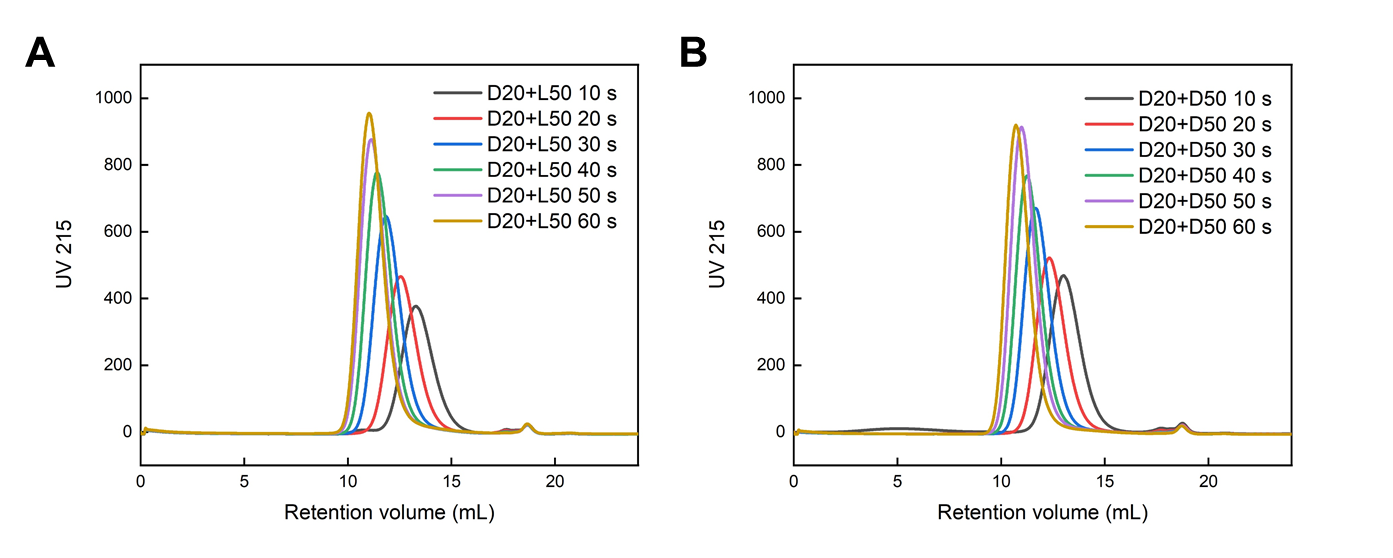


**Figure S13.** Overlay of SEC trances of PDP_20_-initiated ROP of (A) _L_-ProNCA or (B) _D_-ProNCA in mixed ACN/H_2_O at different time.

**Discussion:** The PDP_20_-mediated ROP of (A) _L_-ProNCA or (B) _D_-ProNCA exhibited almost the same reaction rate and *M*_n_ control. This result suggested that the chain growth was not conformation-dependent, which, together with other kinetic evidence, ruled out the cooperative polymerization route.


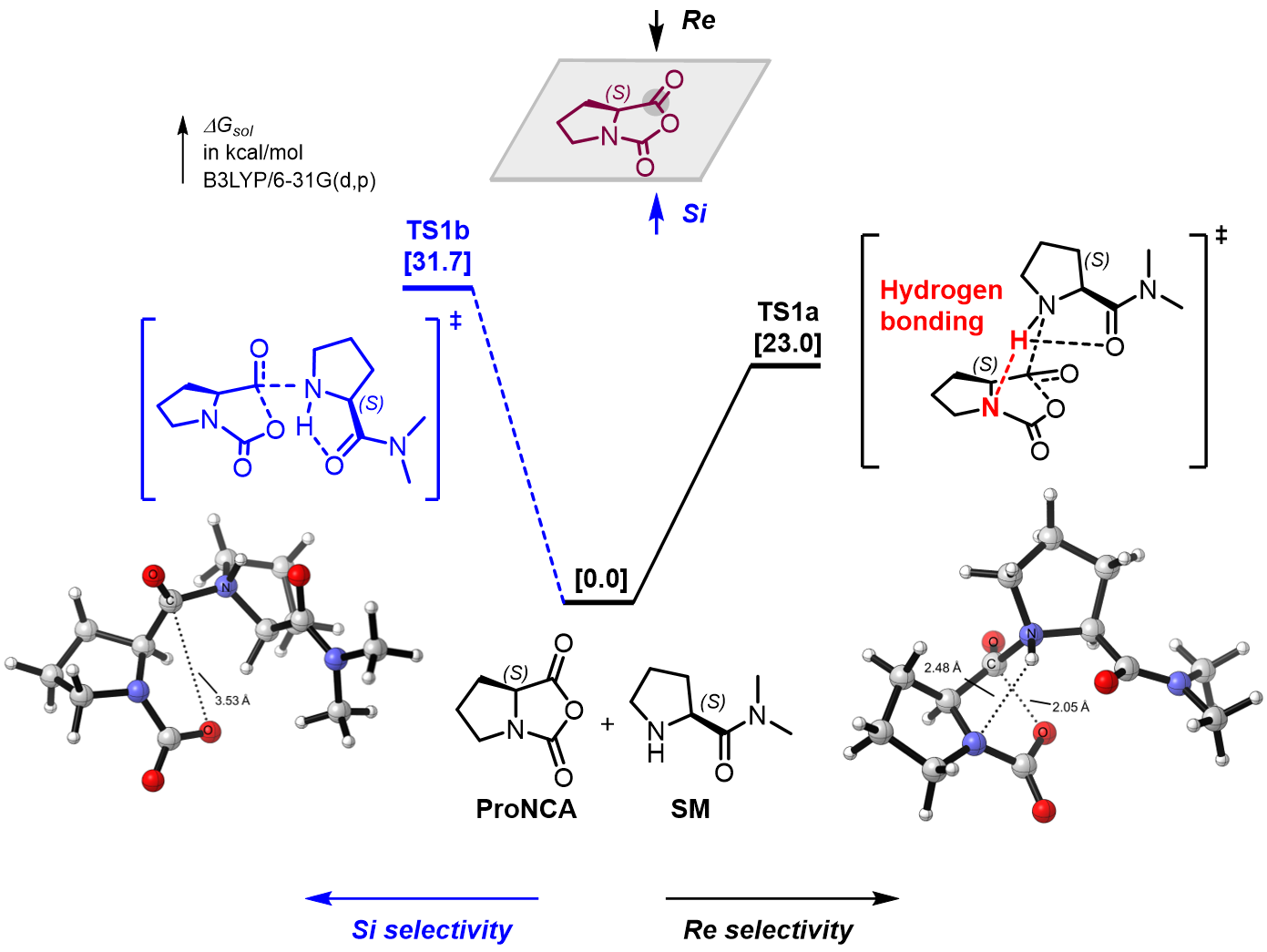


**Figure S14.** Selectivity of nucleophilic addition for the ROP of ProNCA in dry ACN.

**Discussion**: The DFT calculations revealed that **TS1a** was favored over **TS1b** by 8.7 kcal/mol in activation Gibbs free energy. There was an extra N-H hydrogen bonding (2.48 Å in length, the dashed line in red) in **TS1a** stabilizing the transition state. Moreover, the cleaving C-O bond was 3.53 Å in length in **TS1b,** as compared to that of 2.05 Å in **TS1a**, which implied apparent zwitterionic feature of the **TS1b** without extra stabilization effect.


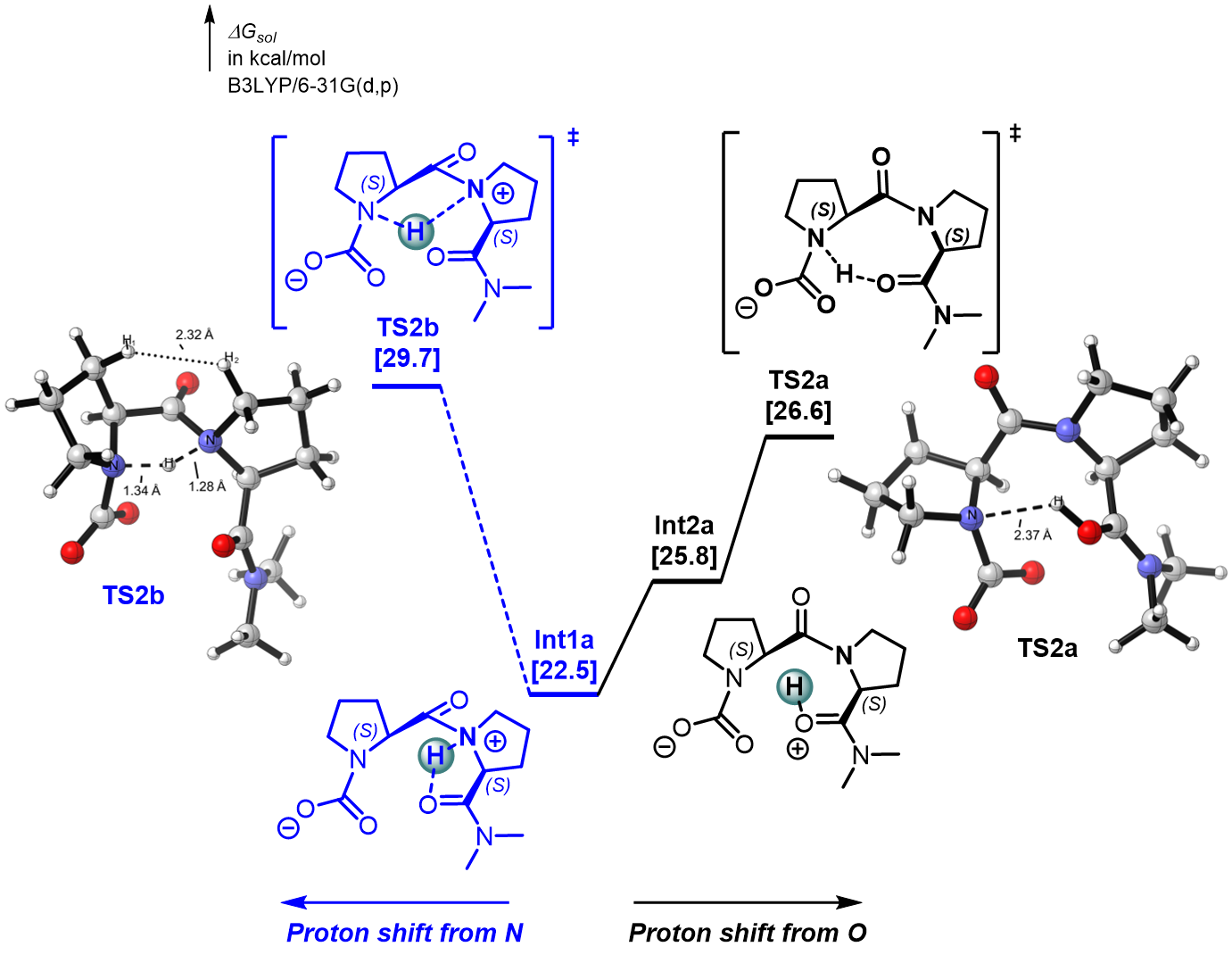


**Figure S15.** Selectivity of proton shift for the ROP of ProNCA in dry ACN.

**Discussion:** In the proton shift step, the **TS2a** was favored over **TS2b** by 3.1 kcal/mol activation Gibbs free energy due to steric hindrance existed in **TS2b** (H-H interaction with 2.32 Å in length).


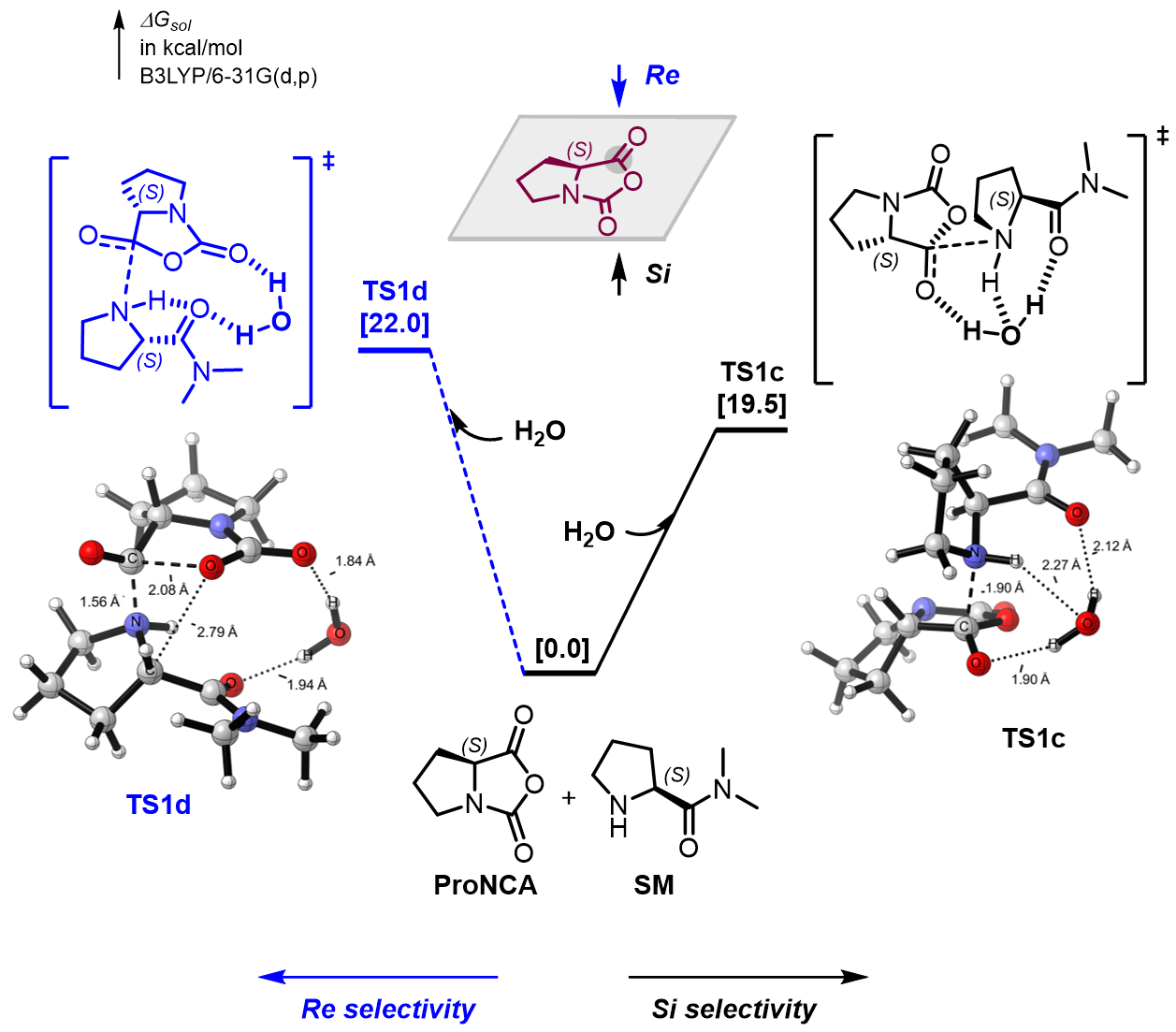


**Figure S16.** Selectivity of nucleophilic addition for the water-assisted ROP of ProNCA.

**Discussion**: In the case of H_2_O-assisted nucleophilic addition, *Si* attack (**TS1C**) was favored over *Re* attack (**TS1d**) by 2.5 kcal/mol due to the existence of steric hindrance raised by the 2.79 Å C-O distance in **TS1d**.


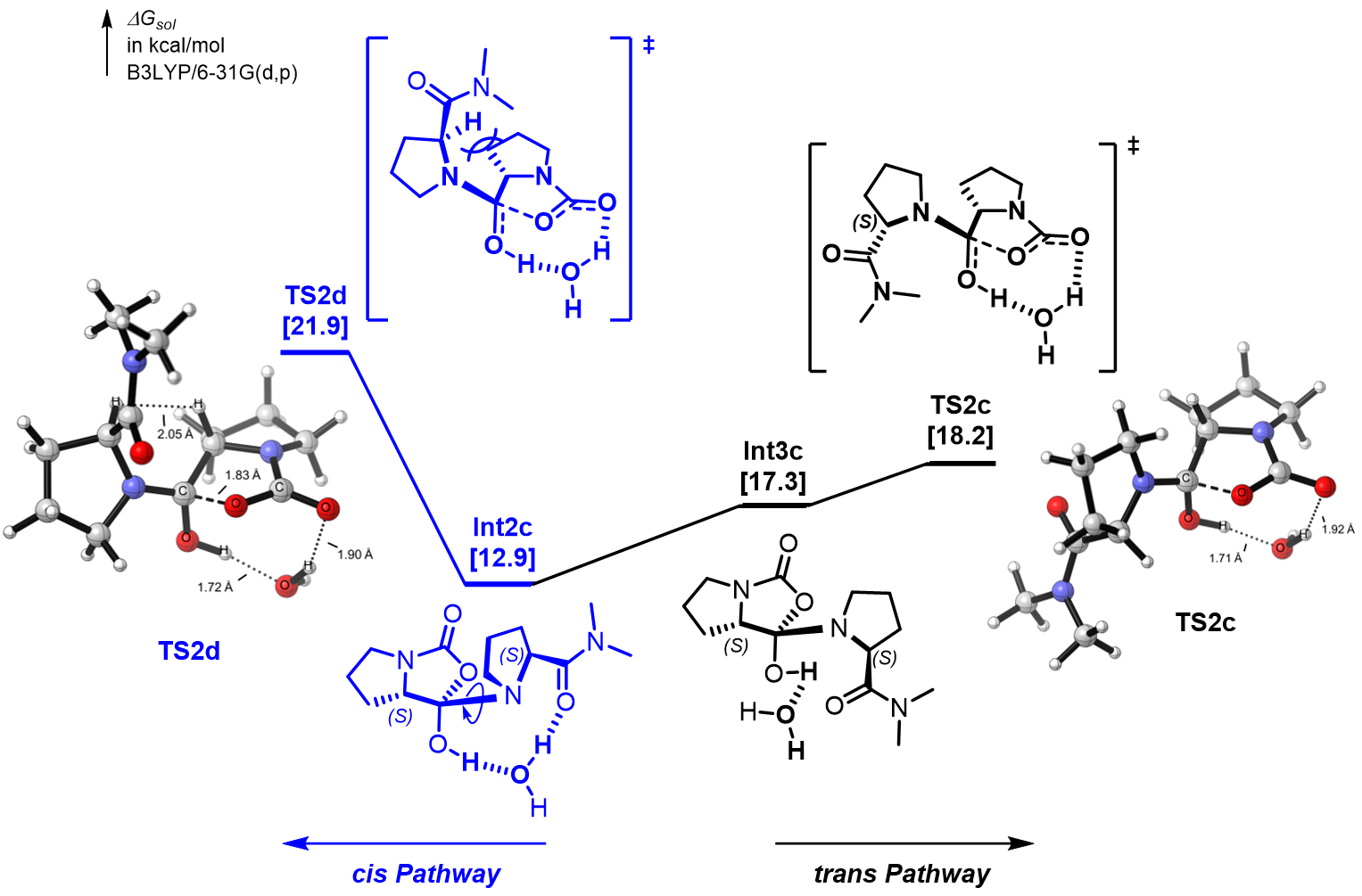


**Figure S17.** Selectivity of the *trans* amide conformation for the water-assisted ROP of ProNCA.

**Discussion:** In the H_2_O-assisted ring opening step, **TS2c** affording a *trans* amide intermediate was favored over **TS2d** that generates a *cis* amide by 3.7 kcal/mol. This is mainly due to the steric hindrance raised by a H-H interaction with 2.05 Å in length in **TS2d**.


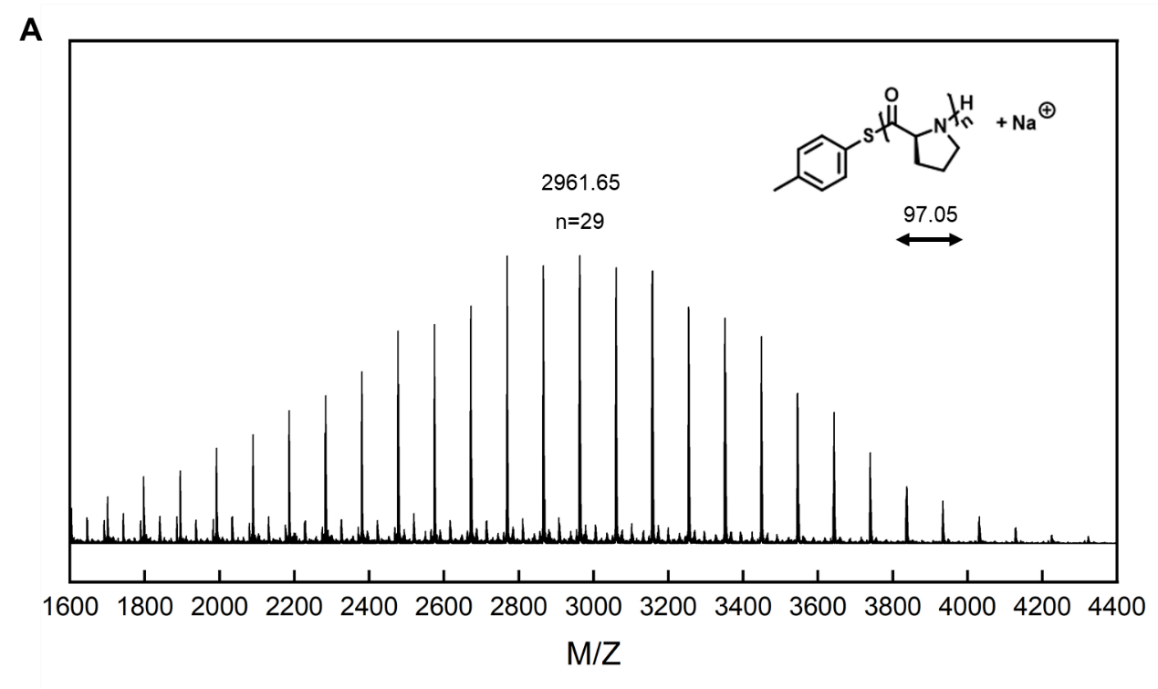


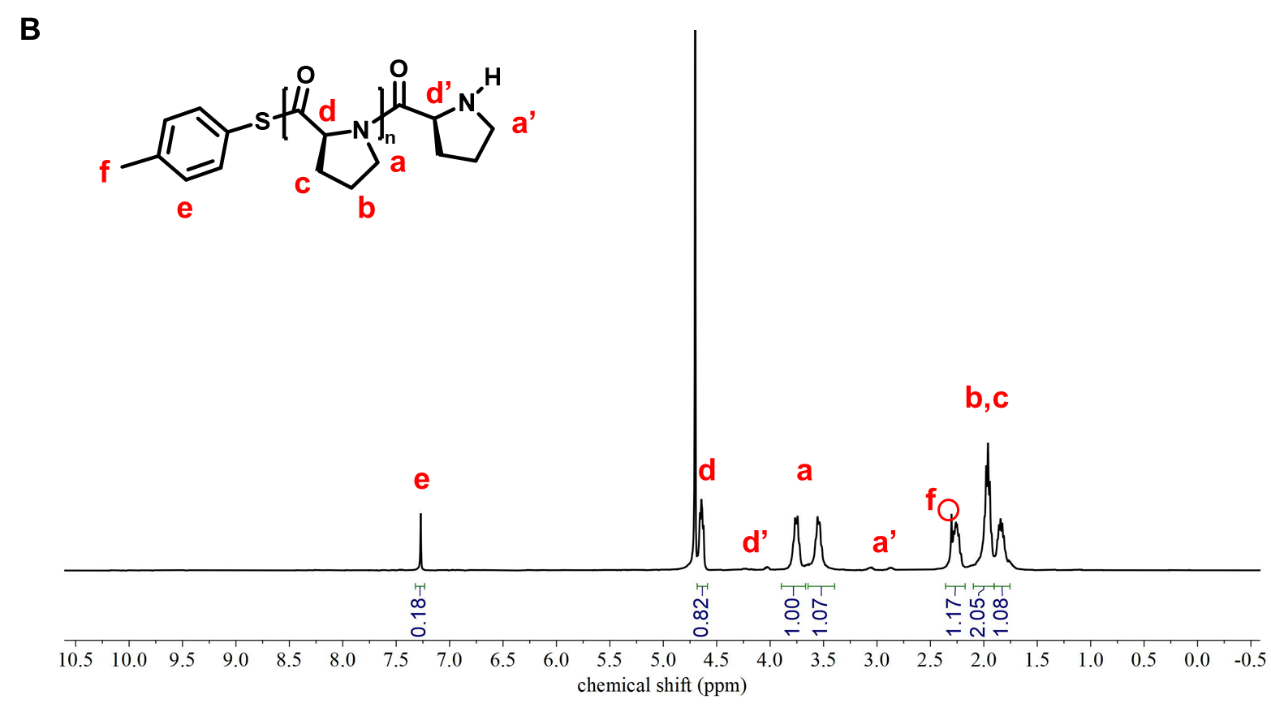


**Figure S18.** (A) MALDI-TOF mass spectrum (M/I = 10/1), and (B) ^1^H NMR spectrum of *p*-MePhSH-mediated ROP of ProNCA (M/I = 25/1)**.**


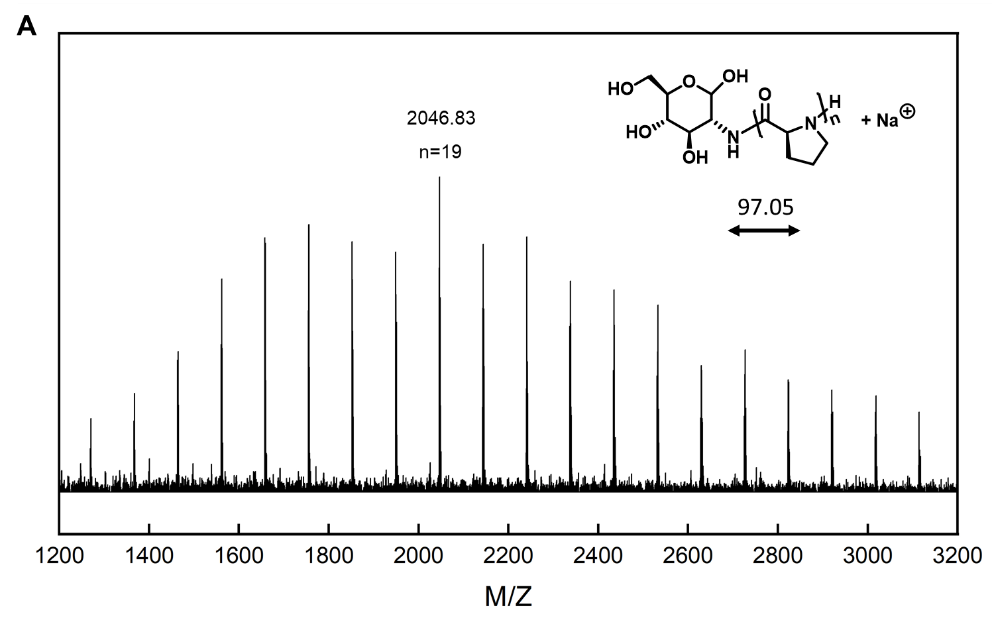


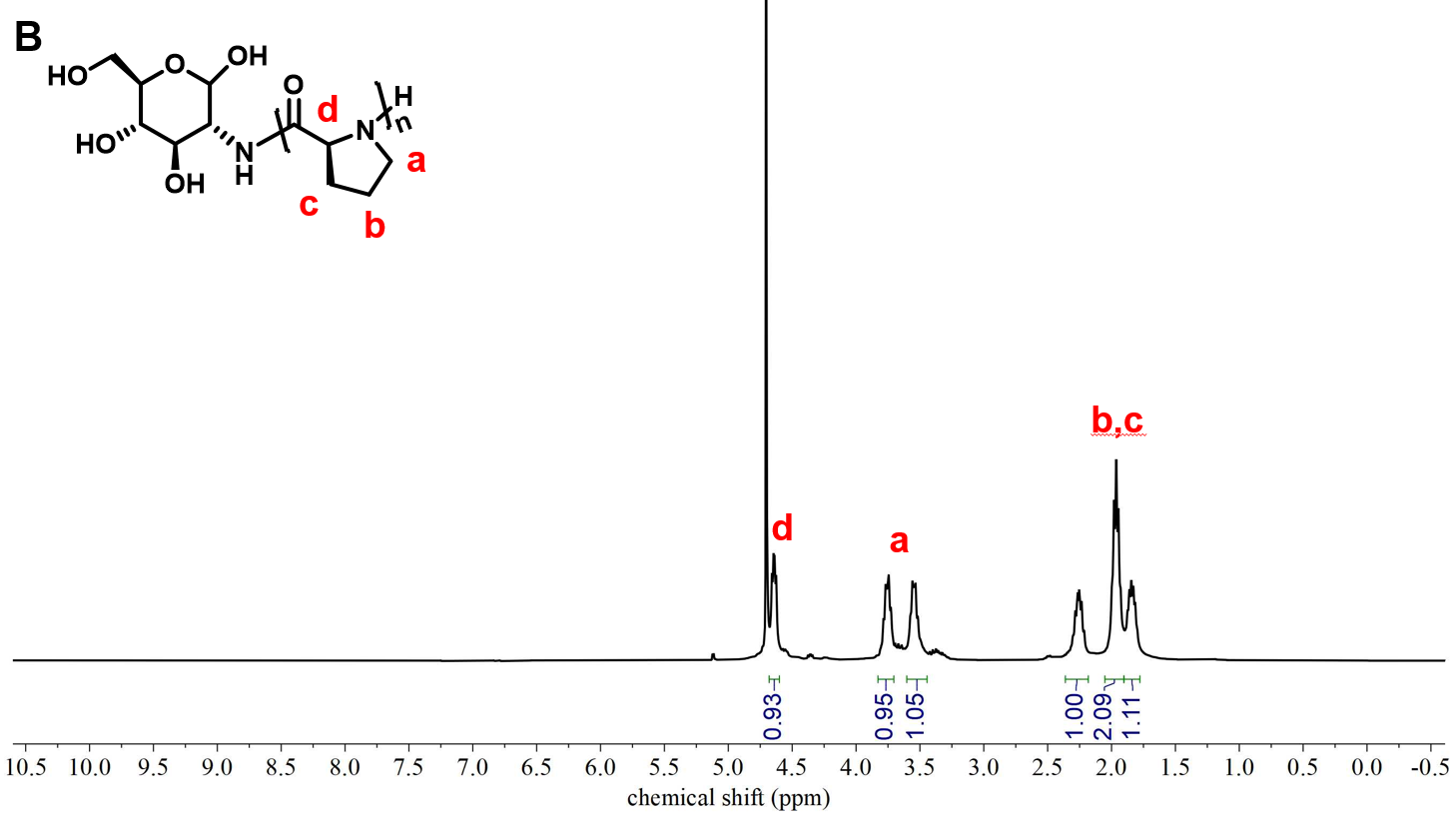


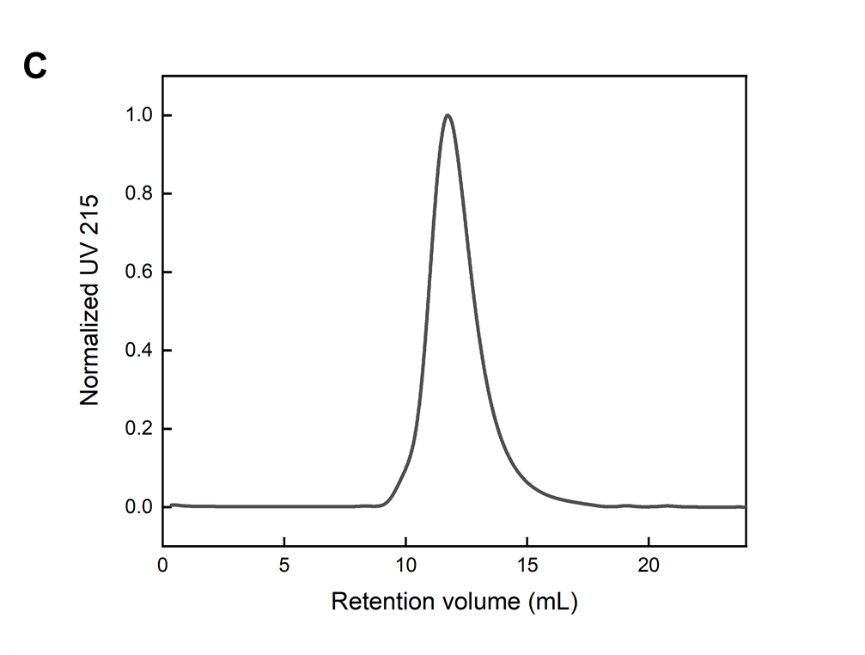


**Figure S19.** (A) MALDI-TOF mass spectrum (M/I = 10/1), (B) ^1^H NMR spectrum (M/I = 25/1), and (C) SEC trace of glucosamine-mediated ROP of ProNCA (M/I = 50/1).


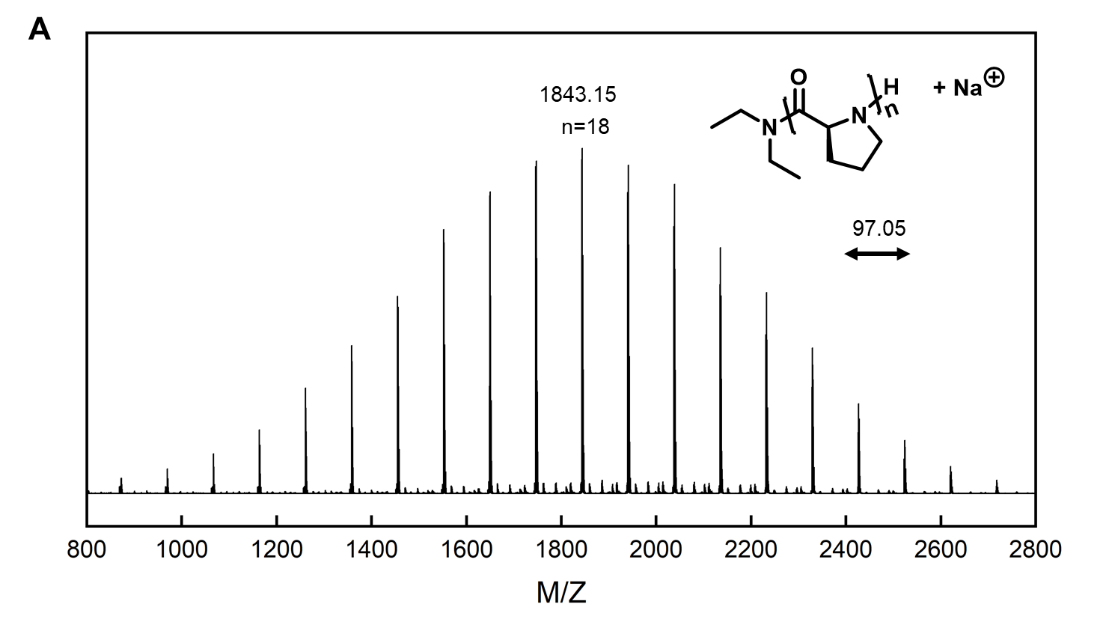


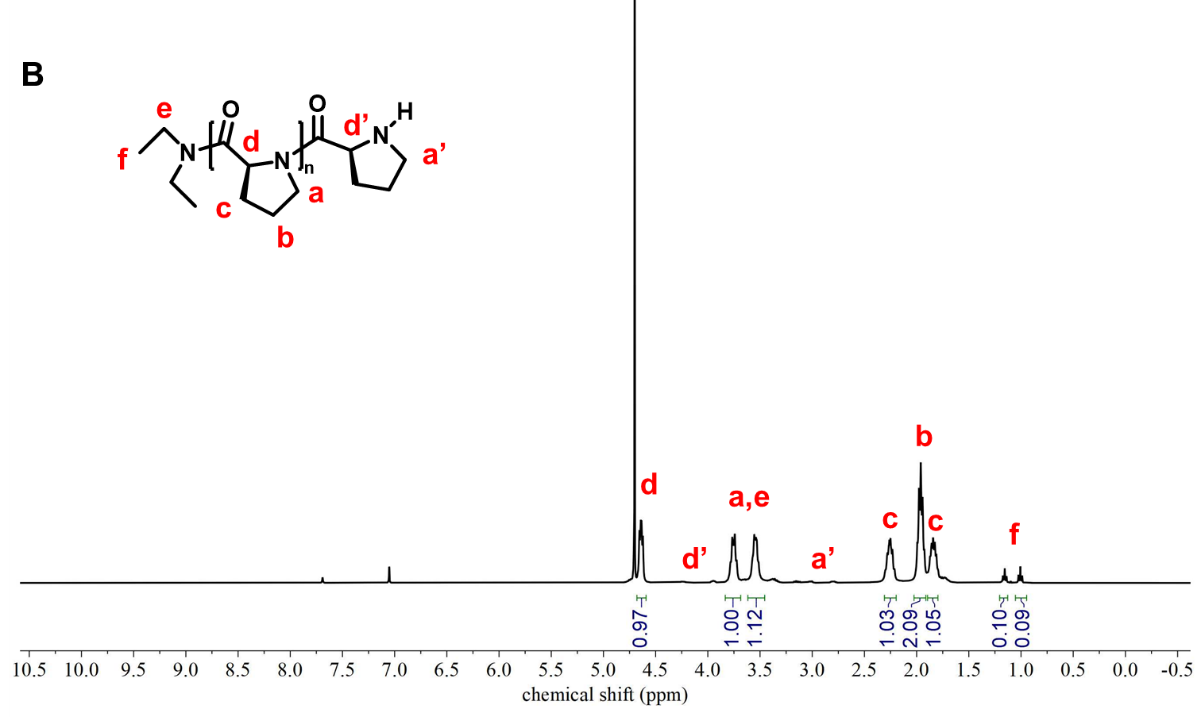


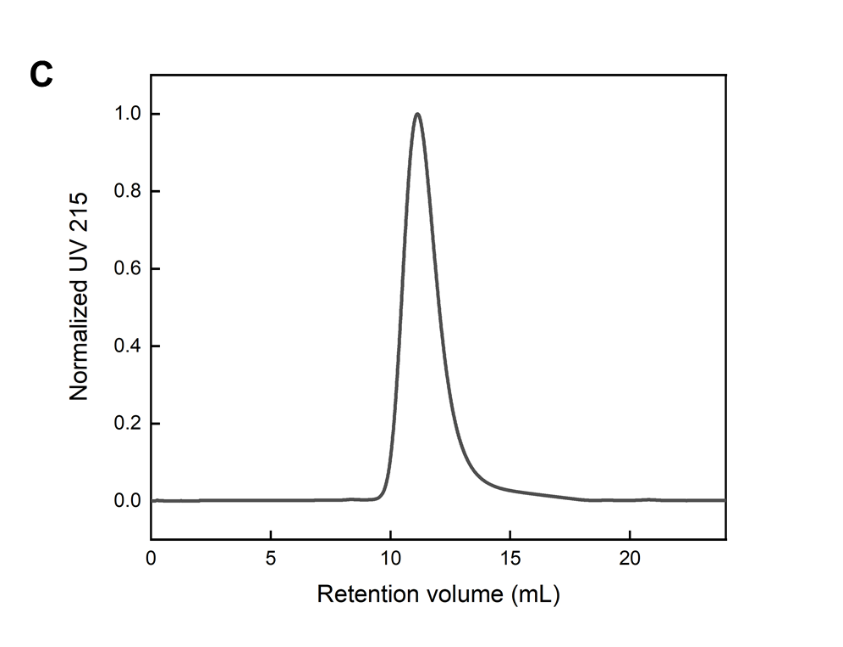


**Figure S20.** (A) MALDI-TOF mass spectrum (M/I = 10/1), (B) ^1^H NMR spectrum (M/I = 25/1), and (C) SEC trace of diethyl amine-mediated ROP of ProNCA (M/I = 50/1).


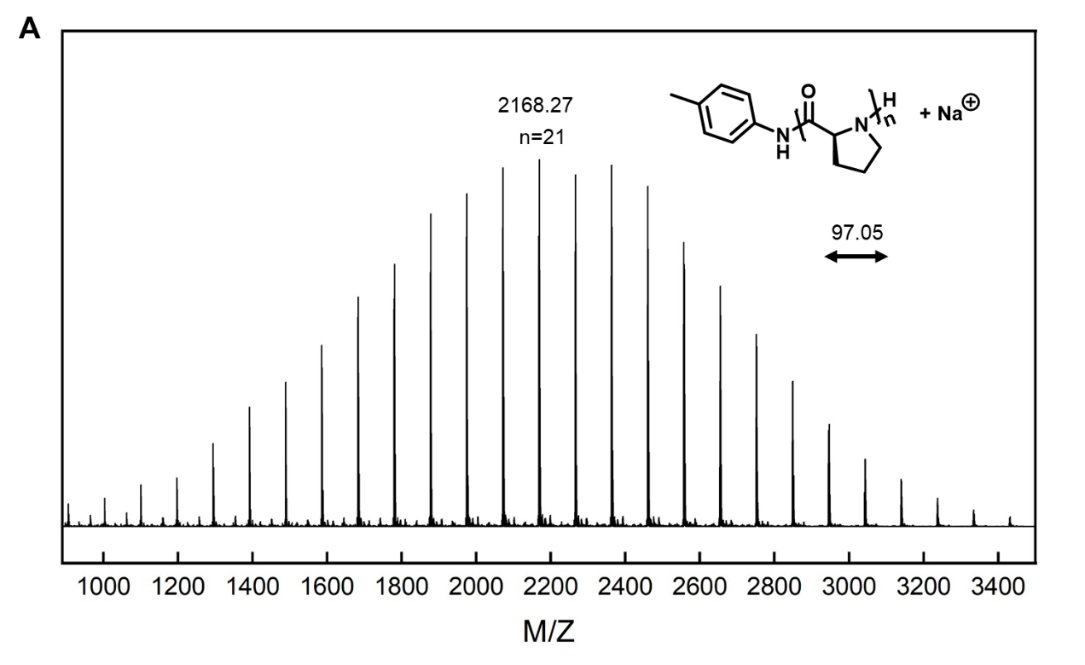


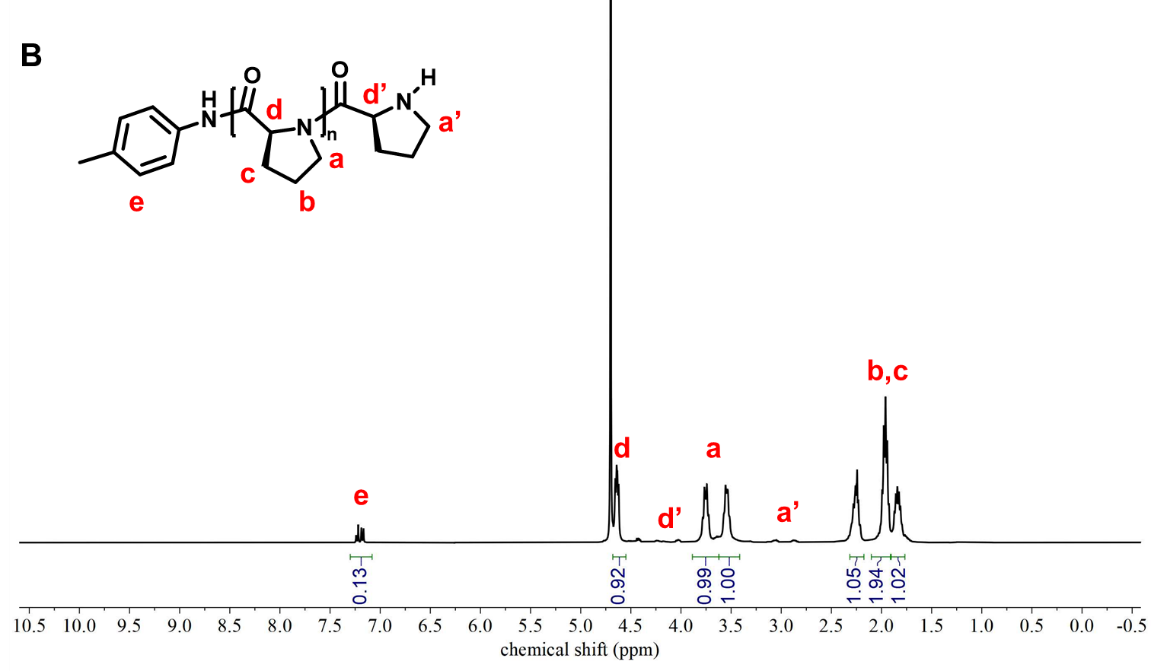


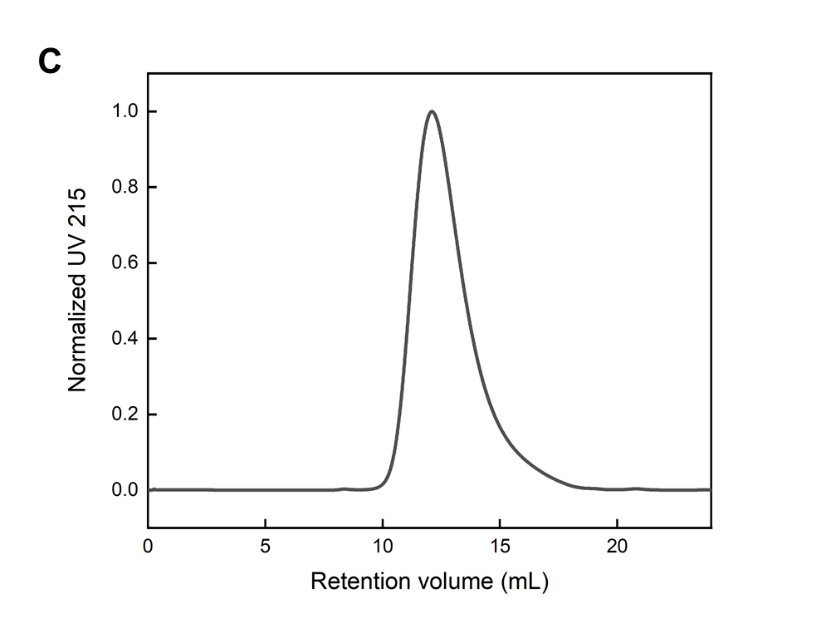


**Figure S21.** (A) MALDI-TOF mass spectrum (M/I = 10/1), (B) ^1^H NMR spectrum (M/I = 25/1), and (C) SEC trace of *p*-toluidine-mediated ROP of ProNCA (M/I = 50/1).


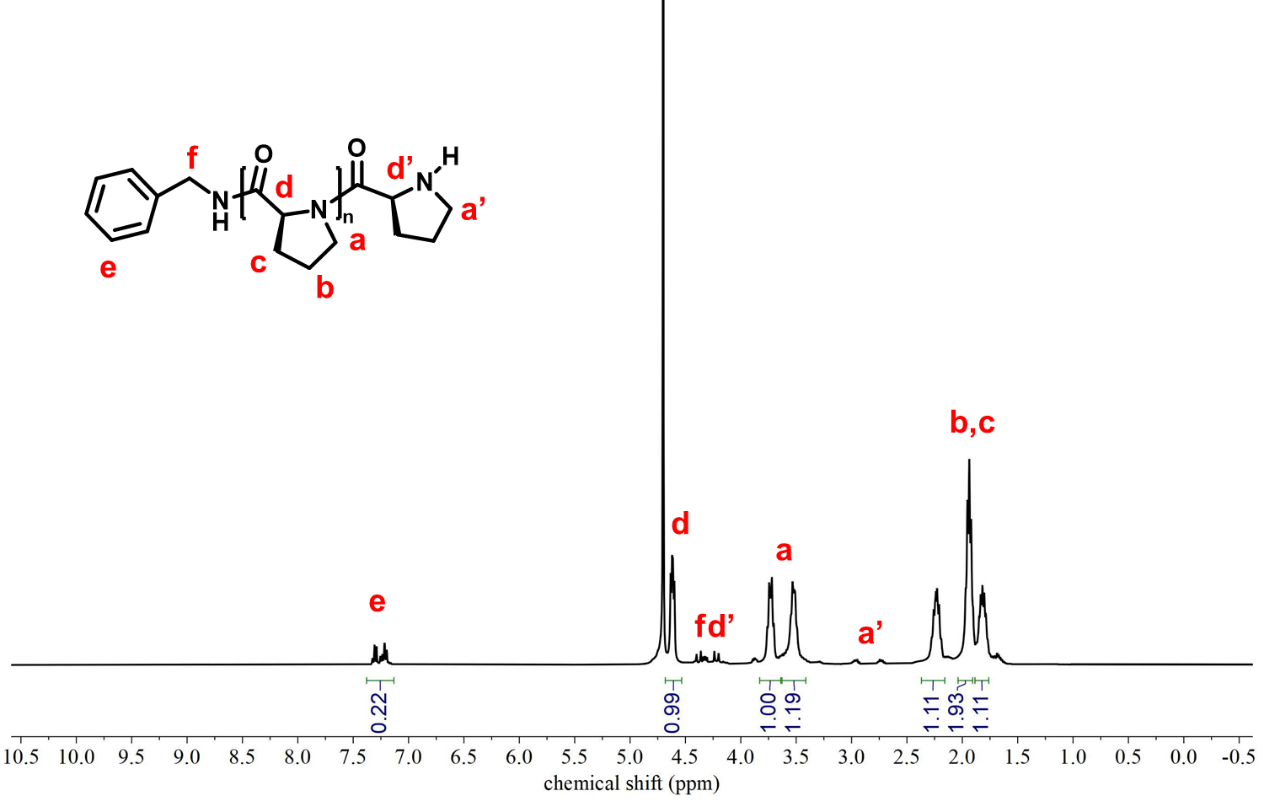


**Figure S22.** ^1^H NMR spectrum of BnNH_2_-mediated ROP of ProNCA (M/I = 25/1).


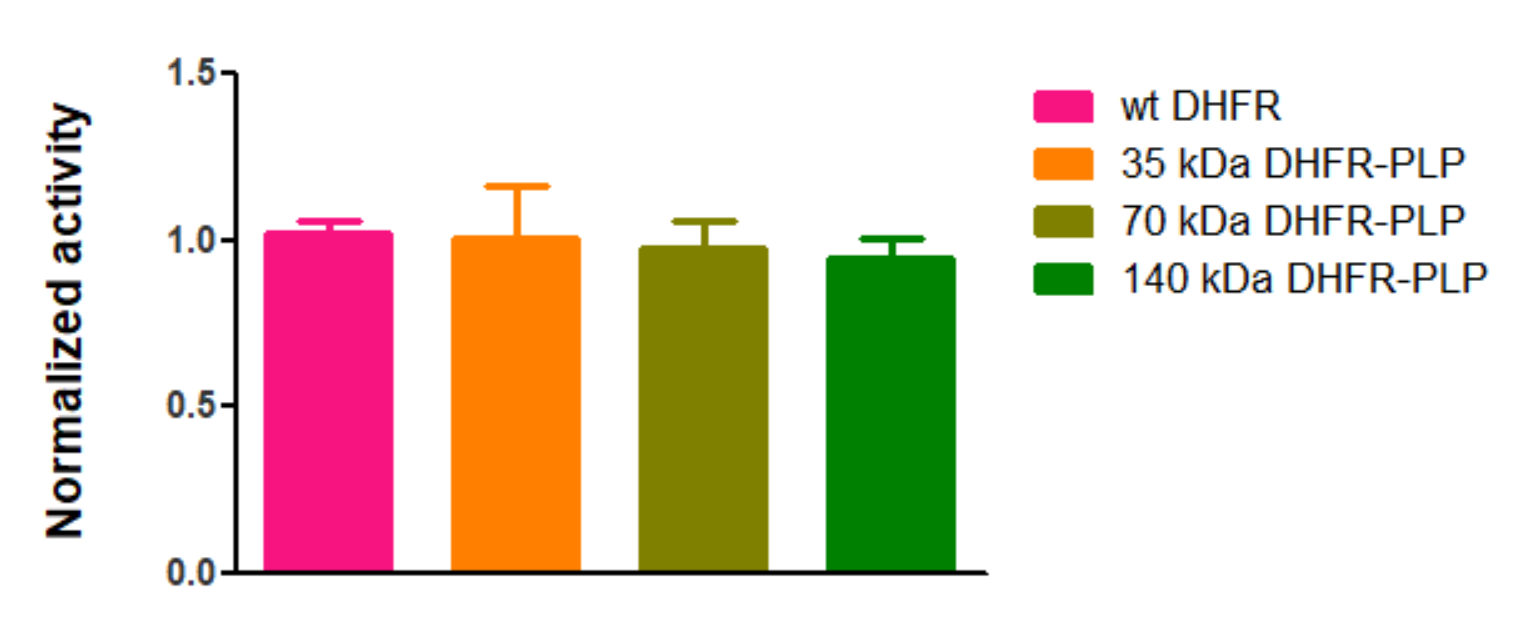


**Figure S23.** Enzymatic activity of wt DHFR and various DHFR-PLP conjugates.


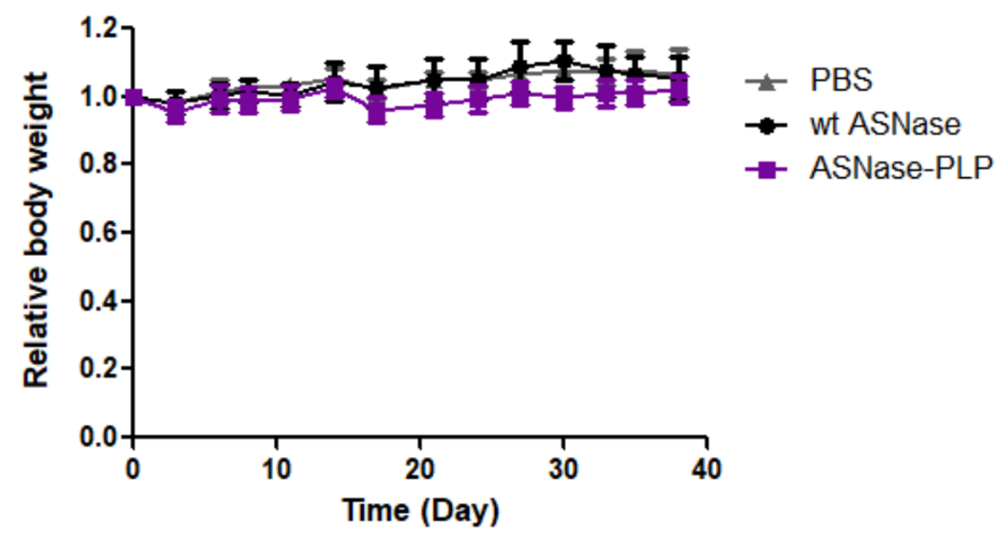


**Figure S24.** The changes of relative body weights of mice bearing NKYS tumors during PBS, wt ASNase, or ASNase-PLP treatment.

**
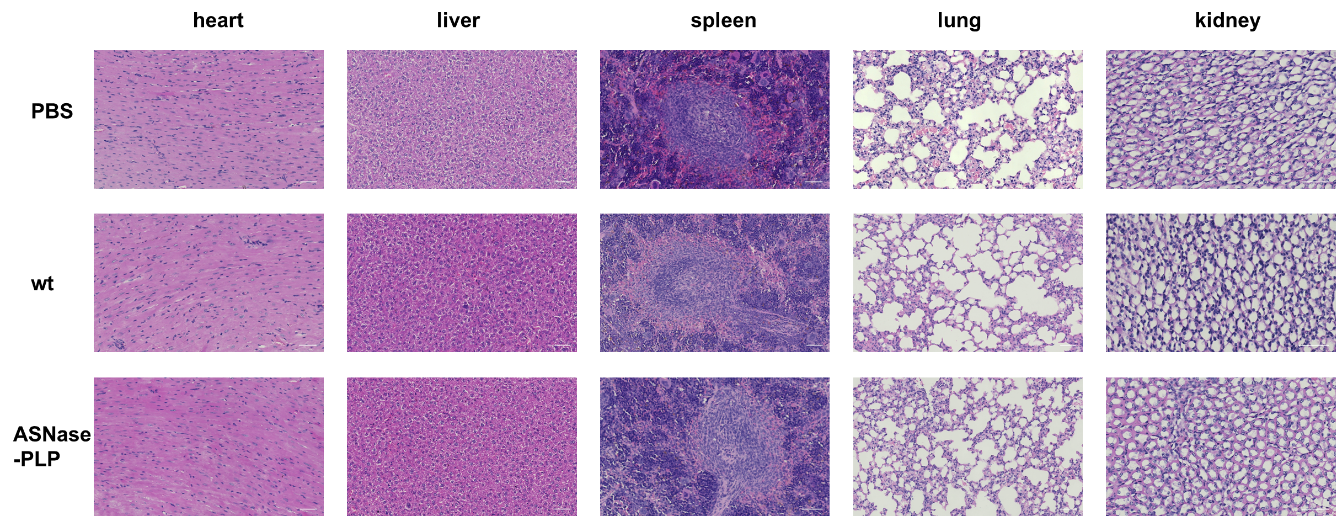
**

**Figure S25.** Histology of the slices of major organs extracted from NKYS tumor-bearing mice receiving PBS, wt ASNase, or ASNase-PLP treatment.

**
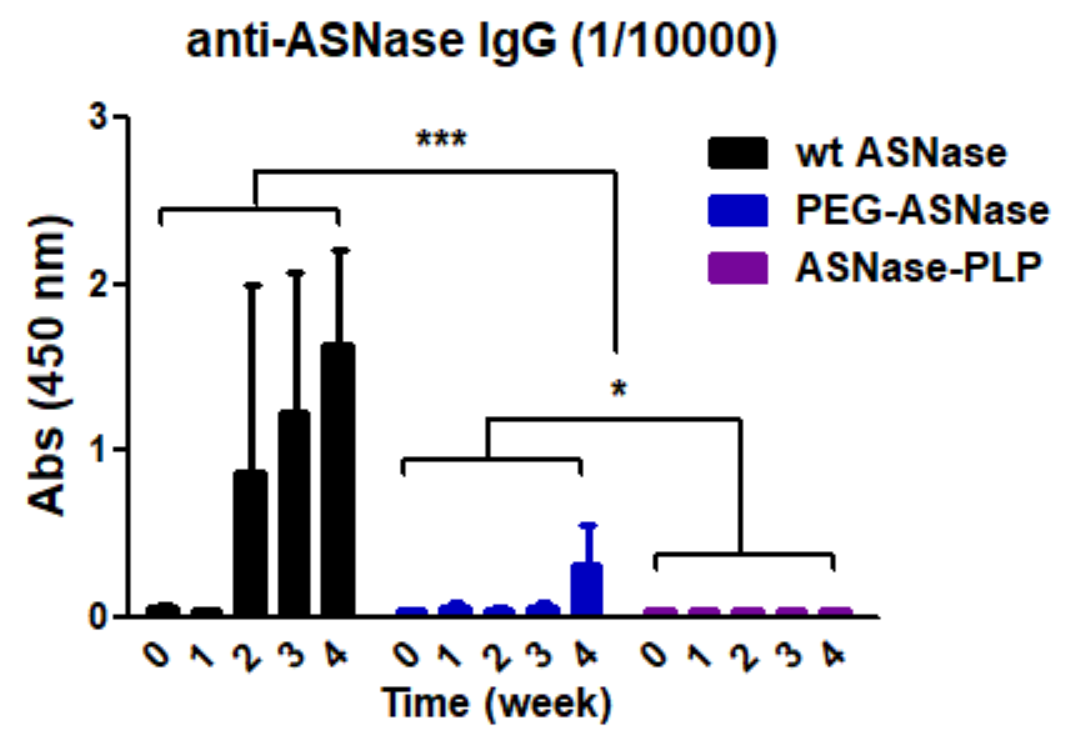
**

**Figure S26.** ELISA analysis of the changes of anti-ASNase IgG levels in the antisera drawn from wt ASNase, ASNase-PEG, or ASNase-PLP infused SD rats.

**Data of DFT calculations**

| **Stationary Points** | **H** | **G** |
| --- | --- | --- |
| **CO_2_** | **-188.565886** | **-188.590216** |
| **H_2_O** | **-76.402193** | **-76.423638** |
| **Int1a** | **-972.87975** | **-972.950069** |
| **Int1c** | **-1049.301613** | **-1049.376905** |
| **Int2a** | **-972.875681** | **-972.944851** |
| **Int2c** | **-1049.311554** | **-1049.386109** |
| **Int3a** | **-972.908783** | **-972.980801** |
| **Int3b** | **-972.898924** | **-972.973251** |
| **Int3c** | **-1049.303342** | **-1049.379056** |
| **Int4a** | **-972.909527** | **-972.983323** |
| **Int4c** | **-1049.311772** | **-1049.387123** |
| **Int5c** | **-1049.325391** | **-1049.405415** |
| **PPI** | **-784.341507** | **-784.407349** |
| **PPII** | **-784.342735** | **-784.408335** |
| **ProNCA** | **-513.186875** | **-513.228557** |
| **SM** | **-459.71146** | **-459.760434** |
| **TS1a** | **-972.880758** | **-972.949236** |
| **TS1b** | **-972.870737** | **-972.938483** |
| **TS1c** | **-1049.300628** | **-1049.375449** |
| **TS1d** | **-1049.299136** | **-1049.371592** |
| **TS2a** | **-972.876498** | **-972.943641** |
| **TS2b** | **-972.869001** | **-972.938627** |
| **TS2c** | **-1049.302852** | **-1049.377555** |
| **TS2d** | **-1049.297523** | **-1049.37172** |
| **TS3a** | **-972.909246** | **-972.982977** |
| **TS3c** | **-1049.309196** | **-1049.386058** |
| **TS4c** | **-1049.322425** | **-1049.403772** |

**All data were in Hartree unit.**

**Coordination of all stationary points**

**CO_2_**

Standard orientation:

---------------------------------------------------------------------

Center Atomic Atomic Coordinates (Angstroms)

Number Number Type X Y Z

---------------------------------------------------------------------

1 6 0 0.000000 0.000000 0.000000

2 8 0 0.000000 0.000000 1.169023

3 8 0 0.000000 0.000000 -1.169023

---------------------------------------------------------------------

###########################################################

**PPI**

Standard orientation:

---------------------------------------------------------------------

Center Atomic Atomic Coordinates (Angstroms)

Number Number Type X Y Z

---------------------------------------------------------------------

1 6 0 -2.254043 0.657146 1.299038

2 6 0 -3.774582 0.766799 1.100658

3 6 0 -3.872009 1.413083 -0.288140

4 7 0 -2.798832 0.796883 -1.091522

5 6 0 -1.694731 0.482353 -0.161202

6 6 0 -1.214101 -0.953807 -0.413936

7 8 0 -1.955890 -1.778486 -0.966661

8 1 0 -1.857187 1.581375 1.731486

9 1 0 -1.967716 -0.165682 1.960354

10 1 0 -4.263855 1.346414 1.890064

11 1 0 -4.231041 -0.229881 1.077496

12 1 0 -3.697388 2.495293 -0.210617

13 1 0 -4.838908 1.267913 -0.779338

14 1 0 -0.864870 1.177886 -0.317923

15 1 0 -3.133038 -0.111483 -1.419210

16 6 0 0.515625 -2.675250 -0.174570

17 6 0 1.022778 -0.416576 0.619111

18 6 0 2.022468 -1.422550 1.238514

19 6 0 1.986497 -2.601461 0.254132

20 1 0 0.384437 -2.977938 -1.216824

21 1 0 0.564829 0.212363 1.382667

22 1 0 3.021037 -0.998982 1.366887

23 1 0 1.652824 -1.729188 2.222552

24 1 0 2.619846 -2.386315 -0.611760

25 1 0 2.332040 -3.535947 0.702841

26 7 0 0.017078 -1.302632 0.026252

27 1 0 -0.059888 -3.369681 0.449098

28 6 0 1.697457 0.451802 -0.472512

29 7 0 2.429923 1.527262 -0.062917

30 8 0 1.573439 0.149055 -1.662595

31 6 0 2.685091 1.917516 1.319102

32 1 0 2.057662 1.373920 2.021281

33 1 0 3.735246 1.739927 1.583574

34 1 0 2.481424 2.987517 1.438224

35 6 0 3.146175 2.320045 -1.056464

36 1 0 4.230047 2.184851 -0.950796

37 1 0 2.839853 2.005358 -2.052091

38 1 0 2.917318 3.382550 -0.919004

---------------------------------------------------------------------

###########################################################

**H_2_O**

Standard orientation:

---------------------------------------------------------------------

Center Atomic Atomic Coordinates (Angstroms)

Number Number Type X Y Z

---------------------------------------------------------------------

1 8 0 0.000000 0.000000 0.120426

2 1 0 -0.000000 0.757243 -0.481705

3 1 0 -0.000000 -0.757243 -0.481705

---------------------------------------------------------------------

###########################################################

**PPII**

Standard orientation:

---------------------------------------------------------------------

Center Atomic Atomic Coordinates (Angstroms)

Number Number Type X Y Z

---------------------------------------------------------------------

1 6 0 -2.739777 0.013931 1.167571

2 6 0 -3.680308 -1.195906 1.037236

3 6 0 -4.398494 -0.911159 -0.289529

4 7 0 -3.374813 -0.334415 -1.181804

5 6 0 -2.442741 0.423820 -0.322918

6 6 0 -1.000240 0.085728 -0.712194

7 8 0 -0.732019 -0.962974 -1.316740

8 1 0 -3.243046 0.839482 1.680787

9 1 0 -1.826436 -0.215559 1.723267

10 1 0 -4.359168 -1.297562 1.890121

11 1 0 -3.101791 -2.123426 0.951764

12 1 0 -5.202020 -0.178151 -0.132092

13 1 0 -4.844373 -1.796788 -0.752240

14 1 0 -2.605596 1.500040 -0.444589

15 6 0 1.369256 0.656143 -0.612445

16 6 0 -0.222019 2.236916 0.371982

17 6 0 1.209828 2.648795 0.742064

18 6 0 2.069033 2.011313 -0.360516

19 1 0 1.469932 0.315295 -1.642819

20 1 0 -0.693919 2.979073 -0.283773

21 1 0 -0.855327 2.106627 1.252665

22 1 0 1.322344 3.734403 0.796467

23 1 0 1.476597 2.227015 1.715430

24 1 0 2.029197 2.610802 -1.276079

25 1 0 3.117493 1.895689 -0.075469

26 7 0 -0.037497 0.964439 -0.351986

27 1 0 -2.817207 -1.103079 -1.559321

28 6 0 1.864898 -0.434230 0.366273

29 7 0 2.981717 -1.136485 0.019372

30 8 0 1.273010 -0.623591 1.433523

31 6 0 3.790841 -0.934619 -1.176874

32 1 0 3.418920 -0.119632 -1.792608

33 1 0 4.823424 -0.699383 -0.891646

34 1 0 3.806462 -1.848706 -1.783034

35 6 0 3.494579 -2.156479 0.926628

36 1 0 2.789114 -2.294033 1.743586

37 1 0 3.624024 -3.102450 0.388454

38 1 0 4.468465 -1.857420 1.334119

---------------------------------------------------------------------

###########################################################

**ProNCA**

Standard orientation:

---------------------------------------------------------------------

Center Atomic Atomic Coordinates (Angstroms)

Number Number Type X Y Z

---------------------------------------------------------------------

1 6 0 -1.080197 -1.484355 -0.479888

2 6 0 -2.353169 -0.674239 -0.157938

3 6 0 -1.856164 0.760430 0.143317

4 7 0 -0.437517 0.558910 0.516163

5 6 0 -0.063180 -0.857151 0.492854

6 6 0 0.562249 1.329936 0.013827

7 8 0 1.714174 0.526563 -0.135509

8 6 0 1.389800 -0.797001 0.077732

9 8 0 2.191146 -1.682617 -0.049125

10 8 0 0.568160 2.498433 -0.288124

11 1 0 -1.199541 -2.557624 -0.322144

12 1 0 -0.759779 -1.312004 -1.512860

13 1 0 -2.842481 -1.090627 0.728120

14 1 0 -3.073935 -0.687198 -0.978548

15 1 0 -2.410201 1.229412 0.959564

16 1 0 -1.903698 1.415181 -0.731194

17 1 0 -0.131621 -1.314270 1.486567

---------------------------------------------------------------------

###########################################################

**SM**

Standard orientation:

---------------------------------------------------------------------

Center Atomic Atomic Coordinates (Angstroms)

Number Number Type X Y Z

---------------------------------------------------------------------

1 6 0 -0.565499 0.191344 -0.600126

2 6 0 -2.868119 -0.276324 -0.272261

3 6 0 -2.419092 0.510404 0.966643

4 6 0 -1.135932 1.193960 0.467820

5 1 0 -0.308961 0.734901 -1.514735

6 1 0 -3.379905 0.394950 -0.976187

7 1 0 -3.548905 -1.103747 -0.050478

8 1 0 -3.166340 1.224202 1.328085

9 1 0 -2.189690 -0.184360 1.783442

10 1 0 -1.371634 2.146912 -0.017367

11 1 0 -0.424076 1.402524 1.271509

12 7 0 -1.631221 -0.790590 -0.890061

13 1 0 -1.352247 -1.638411 -0.393123

14 6 0 0.680774 -0.552086 -0.088280

15 8 0 0.578299 -1.721486 0.308795

16 7 0 1.870708 0.109248 -0.081302

17 6 0 2.071789 1.505129 -0.450290

18 1 0 2.358020 2.095272 0.429325

19 1 0 2.880366 1.578749 -1.186604

20 1 0 1.176239 1.945710 -0.881223

21 6 0 3.058417 -0.554427 0.444193

22 1 0 3.876337 -0.482285 -0.281391

23 1 0 3.385029 -0.080058 1.378192

24 1 0 2.828936 -1.601090 0.633538

---------------------------------------------------------------------

###########################################################

**Int2c**

Standard orientation:

---------------------------------------------------------------------

Center Atomic Atomic Coordinates (Angstroms)

Number Number Type X Y Z

---------------------------------------------------------------------

1 6 0 -3.483528 0.838330 -0.335686

2 6 0 -4.366092 -0.278818 -0.927482

3 6 0 -3.633276 -1.589660 -0.560860

4 7 0 -2.235052 -1.158742 -0.373280

5 6 0 -2.068690 0.289058 -0.560248

6 6 0 -1.468720 -1.618772 0.659598

7 8 0 -0.572395 -0.657169 1.031574

8 6 0 -0.925066 0.646189 0.446313

9 8 0 -1.389373 1.487639 1.425128

10 8 0 -1.519325 -2.711226 1.194295

11 1 0 -3.642756 1.806779 -0.816423

12 1 0 -3.664719 0.951198 0.736720

13 1 0 -4.420984 -0.176761 -2.016648

14 1 0 -5.387905 -0.263167 -0.539703

15 1 0 -3.713806 -2.344379 -1.348539

16 1 0 -4.003296 -2.029164 0.371080

17 1 0 -1.732081 0.506142 -1.580277

18 8 0 0.922246 1.905664 2.748289

19 1 0 0.964683 1.335070 3.530028

20 1 0 -0.597423 1.709500 1.991169

21 6 0 1.192531 0.268608 -0.846949

22 6 0 0.215454 2.482818 -0.767724

23 6 0 1.562297 2.588023 -1.482051

24 6 0 1.789676 1.153155 -1.979139

25 1 0 0.647733 -0.579735 -1.271219

26 1 0 -0.609783 2.536134 -1.498326

27 1 0 0.057027 3.272182 -0.028055

28 1 0 1.551571 3.322691 -2.291493

29 1 0 2.342818 2.873284 -0.768735

30 1 0 1.227633 0.978254 -2.902321

31 1 0 2.840149 0.928650 -2.181272

32 7 0 0.295355 1.166838 -0.116384

33 1 0 1.449261 1.419233 2.067002

34 6 0 2.295196 -0.259720 0.100655

35 8 0 2.559082 0.324785 1.164593

36 7 0 2.986971 -1.364970 -0.280017

37 6 0 2.844806 -2.071161 -1.549496

38 1 0 3.808266 -2.085730 -2.072542

39 1 0 2.537789 -3.108577 -1.371865

40 1 0 2.115094 -1.600266 -2.202667

41 6 0 4.040706 -1.891511 0.582062

42 1 0 3.911870 -2.973033 0.697454

43 1 0 5.029290 -1.707030 0.143417

44 1 0 3.985002 -1.409943 1.556002

---------------------------------------------------------------------

###########################################################

**Int1a**

Standard orientation:

---------------------------------------------------------------------

Center Atomic Atomic Coordinates (Angstroms)

Number Number Type X Y Z

---------------------------------------------------------------------

1 6 0 3.294666 0.918095 -0.369605

2 6 0 4.153273 -0.308308 -0.753979

3 6 0 3.164710 -1.500939 -0.757603

4 7 0 2.008186 -0.989663 -0.020203

5 6 0 2.296070 0.280629 0.613496

6 6 0 1.038196 -1.778075 0.635579

7 8 0 0.264526 -1.052879 1.372921

8 6 0 0.990584 0.982830 0.939614

9 8 0 0.787091 1.714195 1.878859

10 8 0 0.995516 -3.002948 0.444942

11 1 0 3.872937 1.728082 0.081118

12 1 0 2.775235 1.307050 -1.251317

13 1 0 4.925406 -0.466539 0.006147

14 1 0 4.657640 -0.178675 -1.715040

15 1 0 3.589175 -2.387994 -0.272480

16 1 0 2.872955 -1.791481 -1.773921

17 1 0 2.779144 0.165297 1.597300

18 6 0 -1.440186 0.867867 0.158066

19 6 0 0.099855 2.446074 -0.916421

20 6 0 -1.103678 3.208771 -0.378525

21 6 0 -2.190373 2.130324 -0.323071

22 1 0 -1.448809 0.780930 1.240732

23 1 0 1.064153 2.899817 -0.692688

24 1 0 0.022141 2.267069 -1.990720

25 1 0 -0.889415 3.597040 0.621257

26 1 0 -1.367166 4.046249 -1.028981

27 1 0 -3.010693 2.376526 0.353868

28 1 0 -2.607750 1.964166 -1.321611

29 7 0 0.009593 1.078751 -0.243419

30 1 0 0.227228 0.326211 -0.920984

31 6 0 -1.905545 -0.412739 -0.549596

32 8 0 -1.264694 -0.796926 -1.540232

33 7 0 -3.031409 -1.014840 -0.113358

34 6 0 -3.532167 -2.194268 -0.813684

35 1 0 -3.495243 -3.068007 -0.153044

36 1 0 -4.571861 -2.031682 -1.117498

37 1 0 -2.918451 -2.378707 -1.692842

38 6 0 -3.800579 -0.631220 1.065651

39 1 0 -3.817680 -1.460286 1.782007

40 1 0 -3.379527 0.241612 1.558378

41 1 0 -4.832480 -0.402201 0.777728

---------------------------------------------------------------------

###########################################################

**TS1a**

Standard orientation:

---------------------------------------------------------------------

Center Atomic Atomic Coordinates (Angstroms)

Number Number Type X Y Z

---------------------------------------------------------------------

1 6 0 3.259445 0.891211 -0.423969

2 6 0 4.119892 -0.333620 -0.815672

3 6 0 3.151842 -1.542909 -0.749604

4 7 0 2.038662 -1.028489 0.041746

5 6 0 2.314517 0.269219 0.617747

6 6 0 1.014821 -1.749833 0.659118

7 8 0 0.266357 -0.961590 1.378707

8 6 0 0.982441 0.916125 0.977921

9 8 0 0.787858 1.664271 1.911854

10 8 0 0.879136 -2.967054 0.483121

11 1 0 3.847890 1.720070 -0.023575

12 1 0 2.698842 1.248966 -1.293416

13 1 0 4.925023 -0.463752 -0.085204

14 1 0 4.581208 -0.221420 -1.800169

15 1 0 3.620168 -2.415333 -0.277591

16 1 0 2.806619 -1.851639 -1.743689

17 1 0 2.835951 0.198441 1.586391

18 6 0 -1.436085 0.882398 0.132357

19 6 0 0.152537 2.467483 -0.855682

20 6 0 -1.079709 3.220285 -0.368314

21 6 0 -2.165134 2.140295 -0.386408

22 1 0 -1.471457 0.819646 1.216499

23 1 0 1.099204 2.912859 -0.555445

24 1 0 0.142388 2.329174 -1.938690

25 1 0 -0.917936 3.589552 0.648510

26 1 0 -1.310541 4.069688 -1.015755

27 1 0 -3.026371 2.378581 0.241030

28 1 0 -2.519696 1.976646 -1.409530

29 7 0 0.021472 1.079966 -0.236407

30 1 0 0.228242 0.350122 -0.939767

31 6 0 -1.894440 -0.407268 -0.561842

32 8 0 -1.243175 -0.804194 -1.540994

33 7 0 -3.023236 -1.006344 -0.128530

34 6 0 -3.510936 -2.199954 -0.813874

35 1 0 -3.470820 -3.063712 -0.140294

36 1 0 -4.550206 -2.049925 -1.125358

37 1 0 -2.891054 -2.392572 -1.686920

38 6 0 -3.802745 -0.611679 1.040077

39 1 0 -3.804444 -1.423305 1.776549

40 1 0 -3.403053 0.282271 1.512235

41 1 0 -4.838332 -0.412276 0.743884

---------------------------------------------------------------------

###########################################################

**Int1c**

Standard orientation:

---------------------------------------------------------------------

Center Atomic Atomic Coordinates (Angstroms)

Number Number Type X Y Z

---------------------------------------------------------------------

1 6 0 -3.465676 0.525115 -0.635543

2 6 0 -4.116288 -0.789741 -1.106434

3 6 0 -3.262678 -1.906867 -0.459285

4 7 0 -1.975212 -1.244863 -0.163766

5 6 0 -1.977742 0.158520 -0.599639

6 6 0 -1.386520 -1.383324 1.074376

7 8 0 -0.710263 -0.260245 1.402594

8 6 0 -1.112269 0.890355 0.468037

9 8 0 -1.566877 1.905693 1.057380

10 8 0 -1.439420 -2.370130 1.790196

11 1 0 -3.678109 1.367208 -1.298920

12 1 0 -3.793465 0.792863 0.373593

13 1 0 -4.061222 -0.865122 -2.197714

14 1 0 -5.168787 -0.867984 -0.821710

15 1 0 -3.124283 -2.759362 -1.130797

16 1 0 -3.694545 -2.278435 0.474884

17 1 0 -1.540731 0.253245 -1.600091

18 8 0 0.586817 2.932619 2.317686

19 1 0 0.879854 2.232954 2.917664

20 1 0 -0.300917 2.623999 2.009684

21 6 0 1.133958 0.093912 -0.800749

22 6 0 0.358443 2.397627 -1.071788

23 6 0 1.618680 2.207343 -1.908846

24 6 0 1.652682 0.689436 -2.137712

25 1 0 0.458697 -0.744070 -0.965601

26 1 0 -0.542128 2.348458 -1.688358

27 1 0 0.336316 3.304785 -0.470833

28 1 0 1.578745 2.774775 -2.841652

29 1 0 2.499890 2.533870 -1.346564

30 1 0 0.969646 0.408776 -2.944935

31 1 0 2.647071 0.317889 -2.393398

32 7 0 0.363921 1.210977 -0.145345

33 1 0 0.916262 1.481757 0.691955

34 6 0 2.265715 -0.304425 0.166790

35 8 0 2.554245 0.460114 1.098234

36 7 0 2.911978 -1.471191 -0.058331

37 6 0 2.658530 -2.389888 -1.164038

38 1 0 3.513810 -2.402969 -1.849876

39 1 0 2.518900 -3.401693 -0.769121

40 1 0 1.768785 -2.119155 -1.727770

41 6 0 4.029623 -1.848886 0.802678

42 1 0 3.863747 -2.855475 1.200679

43 1 0 4.965438 -1.850998 0.231563

44 1 0 4.107449 -1.139253 1.623614

---------------------------------------------------------------------

###########################################################

**TS1c**

Standard orientation:

---------------------------------------------------------------------

Center Atomic Atomic Coordinates (Angstroms)

Number Number Type X Y Z

---------------------------------------------------------------------

1 6 0 -3.663221 0.486115 -0.305935

2 6 0 -4.209929 -0.783635 -0.988016

3 6 0 -3.166639 -1.887086 -0.677291

4 7 0 -1.945576 -1.133757 -0.315421

5 6 0 -2.146772 0.312873 -0.480255

6 6 0 -1.294565 -1.396225 0.866893

7 8 0 -0.771165 -0.242162 1.378227

8 6 0 -1.258773 0.919359 0.618944

9 8 0 -1.537612 1.934311 1.263768

10 8 0 -1.172409 -2.473480 1.417139

11 1 0 -4.037052 1.408930 -0.755234

12 1 0 -3.911949 0.495389 0.760665

13 1 0 -4.274095 -0.627512 -2.069827

14 1 0 -5.206123 -1.057341 -0.631712

15 1 0 -2.985447 -2.534600 -1.539424

16 1 0 -3.456042 -2.517569 0.167949

17 1 0 -1.836141 0.640782 -1.475888

18 8 0 0.861917 2.395664 2.712533

19 1 0 1.311414 1.553077 2.544030

20 1 0 -0.056054 2.235009 2.416557

21 6 0 1.182631 0.215138 -0.874742

22 6 0 0.319713 2.444707 -1.202007

23 6 0 1.646587 2.363634 -1.957600

24 6 0 1.847599 0.849070 -2.139073

25 1 0 0.503107 -0.589588 -1.159450

26 1 0 -0.526015 2.295051 -1.880899

27 1 0 0.166237 3.371010 -0.647505

28 1 0 1.621339 2.902390 -2.908421

29 1 0 2.450221 2.787765 -1.345724

30 1 0 1.326535 0.495869 -3.033553

31 1 0 2.900055 0.571973 -2.234789

32 7 0 0.384452 1.305623 -0.247106

33 1 0 0.911426 1.616592 0.577828

34 6 0 2.199457 -0.294573 0.163584

35 8 0 2.432808 0.383610 1.177529

36 7 0 2.822798 -1.469701 -0.081672

37 6 0 2.657815 -2.288523 -1.278508

38 1 0 3.585594 -2.295883 -1.862652

39 1 0 2.428400 -3.318426 -0.985103

40 1 0 1.853393 -1.927327 -1.914618

41 6 0 3.827238 -1.962576 0.856903

42 1 0 3.582238 -2.988089 1.152742

43 1 0 4.818038 -1.963919 0.387603

44 1 0 3.844045 -1.321943 1.735866

---------------------------------------------------------------------

###########################################################

**TS1d**

Standard orientation:

---------------------------------------------------------------------

Center Atomic Atomic Coordinates (Angstroms)

Number Number Type X Y Z

---------------------------------------------------------------------

1 6 0 3.159783 0.932656 -0.585559

2 6 0 3.957163 -0.374693 -0.789213

3 6 0 2.978730 -1.501290 -0.387074

4 7 0 2.028273 -0.807372 0.476846

5 6 0 2.314205 0.599333 0.660275

6 6 0 0.934626 -1.328202 1.143349

7 8 0 0.220971 -0.405045 1.703659

8 6 0 1.003100 1.346985 0.905997

9 8 0 0.844000 2.261603 1.680966

10 8 0 0.707761 -2.558303 1.150734

11 1 0 3.795859 1.808727 -0.440573

12 1 0 2.516279 1.113498 -1.452706

13 1 0 4.826189 -0.383336 -0.122953

14 1 0 4.321917 -0.485424 -1.813508

15 1 0 3.488815 -2.314577 0.142110

16 1 0 2.456882 -1.941080 -1.245285

17 1 0 2.897778 0.790322 1.573446

18 6 0 -1.428666 1.131020 0.062749

19 6 0 0.138955 2.601878 -1.125807

20 6 0 -1.125035 3.376829 -0.773750

21 6 0 -2.178437 2.270703 -0.658159

22 1 0 -1.488168 1.229603 1.143171

23 1 0 1.068339 3.110877 -0.875210

24 1 0 0.154853 2.308567 -2.177228

25 1 0 -0.997606 3.892055 0.182713

26 1 0 -1.365853 4.116699 -1.540686

27 1 0 -3.064055 2.571913 -0.095184

28 1 0 -2.496256 1.944337 -1.653751

29 7 0 0.032067 1.313084 -0.314846

30 1 0 0.248916 0.501601 -0.918020

31 6 0 -1.818905 -0.270767 -0.426356

32 8 0 -1.142011 -0.759580 -1.354759

33 7 0 -2.872378 -0.896238 0.115293

34 6 0 -3.221541 -2.235075 -0.367095

35 1 0 -2.321216 -2.768030 -0.672896

36 1 0 -3.704782 -2.780449 0.446238

37 1 0 -3.915365 -2.178806 -1.213879

38 6 0 -3.749486 -0.340972 1.143197

39 1 0 -3.702672 -0.959288 2.045522

40 1 0 -3.472095 0.678126 1.401514

41 1 0 -4.781772 -0.333147 0.778328

42 8 0 0.051985 -3.374228 -1.443213

43 1 0 0.346916 -3.232253 -0.519067

44 1 0 -0.377240 -2.522257 -1.627565

---------------------------------------------------------------------

###########################################################

**Int5c**

Standard orientation:

---------------------------------------------------------------------

Center Atomic Atomic Coordinates (Angstroms)

Number Number Type X Y Z

---------------------------------------------------------------------

1 6 0 -1.579712 0.912458 1.961168

2 6 0 -2.798076 0.137358 2.510647

3 6 0 -3.718875 -0.006195 1.302068

4 7 0 -2.765216 -0.240816 0.169939

5 6 0 -1.611699 0.681989 0.416203

6 6 0 -3.470027 -0.034972 -1.303464

7 8 0 -3.757583 1.134220 -1.506237

8 6 0 -0.323386 0.071923 -0.136941

9 8 0 -0.210367 -1.152253 -0.317084

10 8 0 -3.575161 -1.106880 -1.902158

11 1 0 -1.665528 1.981512 2.167165

12 1 0 -0.641761 0.561320 2.396645

13 1 0 -3.289659 0.659265 3.334957

14 1 0 -2.495585 -0.852306 2.866775

15 1 0 -4.256292 0.919041 1.081362

16 1 0 -4.422529 -0.838661 1.351371

17 1 0 -1.857084 1.608013 -0.105658

18 6 0 1.993030 0.410778 -0.804367

19 6 0 0.671453 2.393634 -0.217704

20 6 0 2.146823 2.783958 -0.378154

21 6 0 2.719368 1.686412 -1.287634

22 1 0 1.843440 -0.317141 -1.601604

23 1 0 0.049106 2.847522 -0.997676

24 1 0 0.272194 2.681050 0.757269

25 1 0 2.258766 3.786449 -0.797624

26 1 0 2.643943 2.761574 0.595859

27 1 0 2.455336 1.873927 -2.333537

28 1 0 3.806140 1.598758 -1.221173

29 7 0 0.685803 0.924217 -0.382049

30 1 0 -2.772003 -2.636563 -1.121761

31 8 0 -2.271895 -3.104389 -0.423606

32 1 0 -2.447459 -1.223760 0.207364

33 1 0 -1.386689 -2.706951 -0.497058

34 6 0 2.709130 -0.243250 0.401727

35 7 0 3.734260 -1.101389 0.138040

36 8 0 2.362030 0.040052 1.552509

37 6 0 4.263653 -1.432406 -1.179875

38 1 0 3.712519 -0.942451 -1.978325

39 1 0 5.313691 -1.123333 -1.250875

40 1 0 4.216736 -2.515904 -1.341086

41 6 0 4.459403 -1.708846 1.248331

42 1 0 3.947328 -1.475402 2.179647

43 1 0 4.501497 -2.795408 1.113445

44 1 0 5.487256 -1.327663 1.293573

---------------------------------------------------------------------

###########################################################

**TS3c**

Standard orientation:

---------------------------------------------------------------------

Center Atomic Atomic Coordinates (Angstroms)

Number Number Type X Y Z

---------------------------------------------------------------------

1 6 0 -1.669361 0.719153 1.984525

2 6 0 -3.147070 0.440137 2.371872

3 6 0 -3.873986 0.239117 1.035774

4 7 0 -2.803015 -0.265428 0.128021

5 6 0 -1.634207 0.602881 0.438253

6 6 0 -3.211693 -0.200798 -1.332612

7 8 0 -3.448782 0.892496 -1.823689

8 6 0 -0.322718 0.011089 -0.084980

9 8 0 -0.162759 -1.213217 -0.232850

10 8 0 -3.264115 -1.354113 -1.891583

11 1 0 -1.330490 1.708457 2.298872

12 1 0 -1.002487 -0.020633 2.434738

13 1 0 -3.591442 1.261704 2.938669

14 1 0 -3.214815 -0.462788 2.984405

15 1 0 -4.259181 1.185159 0.639683

16 1 0 -4.689755 -0.486978 1.075898

17 1 0 -1.817466 1.580500 -0.018045

18 6 0 1.982932 0.388405 -0.772937

19 6 0 0.629979 2.355034 -0.210364

20 6 0 2.096001 2.770500 -0.392059

21 6 0 2.678889 1.668267 -1.288783

22 1 0 1.838844 -0.357482 -1.554383

23 1 0 -0.006433 2.784516 -0.992857

24 1 0 0.233717 2.652503 0.762954

25 1 0 2.184898 3.767803 -0.829286

26 1 0 2.603596 2.773806 0.576768

27 1 0 2.398132 1.831903 -2.334410

28 1 0 3.768067 1.602853 -1.235325

29 7 0 0.669819 0.883106 -0.346602

30 1 0 -2.852627 -2.237137 -1.071234

31 8 0 -2.459131 -2.706490 -0.081380

32 1 0 -2.578219 -1.630451 0.293960

33 1 0 -1.485773 -2.684301 -0.176881

34 6 0 2.729674 -0.227628 0.433670

35 7 0 3.763672 -1.076867 0.170672

36 8 0 2.399021 0.075891 1.583605

37 6 0 4.287102 -1.414193 -1.147851

38 1 0 3.694066 -0.979106 -1.948088

39 1 0 5.317529 -1.051337 -1.249757

40 1 0 4.296249 -2.502674 -1.277980

41 6 0 4.523168 -1.639185 1.281623

42 1 0 4.017112 -1.403236 2.215637

43 1 0 4.597642 -2.726306 1.167086

44 1 0 5.539384 -1.225664 1.305320

---------------------------------------------------------------------

###########################################################

**Int3b**

Standard orientation:

---------------------------------------------------------------------

Center Atomic Atomic Coordinates (Angstroms)

Number Number Type X Y Z

---------------------------------------------------------------------

1 7 0 -0.445425 0.846616 -0.000938

2 1 0 1.821206 -0.047295 -1.162104

3 7 0 2.437917 -0.165796 -0.359734

4 6 0 3.565585 0.823332 -0.414395

5 6 0 1.727700 -0.015066 0.952136

6 6 0 3.055884 -1.870107 -0.664674

7 8 0 2.749114 -2.582029 0.261879

8 6 0 0.196798 0.077418 0.914240

9 8 0 -0.436341 -0.556703 1.766828

10 8 0 3.612403 -1.843691 -1.738038

11 1 0 2.441854 0.995599 2.757393

12 1 0 1.944682 2.103562 1.470043

13 1 0 4.451942 0.336885 1.500753

14 1 0 4.379526 2.079169 1.177761

15 1 0 4.400021 0.377951 -0.956821

16 1 0 3.236302 1.717888 -0.950816

17 1 0 1.916212 -0.948077 1.485607

18 6 0 -1.918200 0.865821 0.023831

19 6 0 0.099048 1.811968 -0.972064

20 6 0 -1.046376 2.816747 -1.129710

21 6 0 -2.295113 1.930519 -1.033304

22 1 0 -2.238004 1.162387 1.024221

23 1 0 1.011722 2.280319 -0.604364

24 1 0 0.311839 1.319754 -1.930653

25 1 0 -1.022030 3.537163 -0.304912

26 1 0 -0.984512 3.368160 -2.071366

27 1 0 -3.196694 2.478433 -0.750446

28 1 0 -2.479635 1.443546 -1.996661

29 6 0 2.441977 1.149928 1.676113

30 6 0 3.846220 1.133496 1.054754

31 6 0 -2.485342 -0.517190 -0.352430

32 8 0 -1.863597 -1.239962 -1.135295

33 7 0 -3.707316 -0.853459 0.150246

34 6 0 -4.259103 -2.171402 -0.144441

35 1 0 -5.261170 -2.074780 -0.576981

36 1 0 -3.604358 -2.677977 -0.850860

37 1 0 -4.335541 -2.769667 0.772157

38 6 0 -4.466839 -0.057414 1.111000

39 1 0 -4.310646 1.010795 0.964450

40 1 0 -5.531462 -0.255989 0.960388

41 1 0 -4.213547 -0.318579 2.146877

---------------------------------------------------------------------

###########################################################

**TS2b**

Standard orientation:

---------------------------------------------------------------------

Center Atomic Atomic Coordinates (Angstroms)

Number Number Type X Y Z

---------------------------------------------------------------------

1 7 0 0.129404 1.070823 -0.185445

2 1 0 0.689348 -0.015448 -0.579033

3 7 0 1.650986 -0.858424 -0.183068

4 6 0 2.732704 -1.193722 -1.148553

5 6 0 2.154868 0.133344 0.805026

6 6 0 0.864088 -1.984472 0.483417

7 8 0 0.139316 -1.533991 1.398849

8 6 0 1.025763 1.140139 0.991835

9 8 0 0.867146 1.890422 1.919018

10 8 0 1.032807 -3.116963 0.022992

11 1 0 4.130011 1.076535 0.941373

12 1 0 3.237336 1.485760 -0.534601

13 1 0 4.472011 -1.233840 0.149912

14 1 0 4.732144 -0.292910 -1.329903

15 1 0 2.781798 -2.279054 -1.241141

16 1 0 2.485620 -0.766113 -2.125900

17 1 0 2.352093 -0.326701 1.775334

18 6 0 -1.333474 0.965557 0.152973

19 6 0 0.296127 2.262610 -1.097013

20 6 0 -0.818261 3.211356 -0.668258

21 6 0 -1.992282 2.258022 -0.408857

22 1 0 -1.414144 0.919081 1.236854

23 1 0 1.302892 2.673526 -1.006918

24 1 0 0.149427 1.900467 -2.117872

25 1 0 -0.532570 3.735838 0.248762

26 1 0 -1.038727 3.955542 -1.438005

27 1 0 -2.726196 2.660718 0.292481

28 1 0 -2.507486 2.035934 -1.348569

29 6 0 3.455485 0.683704 0.177574

30 6 0 3.996988 -0.550338 -0.562755

31 6 0 -1.895275 -0.292785 -0.527129

32 8 0 -1.348486 -0.715541 -1.552104

33 7 0 -3.029807 -0.835098 -0.014372

34 6 0 -3.570761 -2.047040 -0.621789

35 1 0 -4.647522 -1.932594 -0.783035

36 1 0 -3.073332 -2.221102 -1.573814

37 1 0 -3.407144 -2.911769 0.033628

38 6 0 -3.636999 -0.455242 1.258422

39 1 0 -3.457925 0.591999 1.496110

40 1 0 -4.718853 -0.596036 1.184955

41 1 0 -3.262948 -1.079167 2.080176

---------------------------------------------------------------------

###########################################################

**Int2a**

Standard orientation:

---------------------------------------------------------------------

Center Atomic Atomic Coordinates (Angstroms)

Number Number Type X Y Z

---------------------------------------------------------------------

1 7 0 -0.334051 1.610769 -0.142842

2 1 0 -0.541352 0.221478 -1.558059

3 7 0 1.803944 -0.628828 -0.348244

4 6 0 3.183449 -0.656256 -0.851756

5 6 0 1.730395 0.432816 0.681554

6 6 0 1.165631 -1.897846 -0.040852

7 8 0 0.190005 -1.822982 0.767372

8 6 0 1.040975 1.660767 0.066296

9 8 0 1.662295 2.641914 -0.333183

10 8 0 1.605354 -2.909963 -0.626141

11 1 0 3.309203 0.705646 2.189574

12 1 0 3.465464 1.743335 0.765156

13 1 0 4.146810 -1.222348 0.997042

14 1 0 5.044104 0.033222 0.124915

15 1 0 3.373823 -1.637815 -1.284418

16 1 0 3.319599 0.108384 -1.629538

17 1 0 1.135232 0.046928 1.509366

18 6 0 -1.302893 0.774905 0.594748

19 6 0 -0.977790 2.842727 -0.678780

20 6 0 -2.473255 2.597275 -0.485174

21 6 0 -2.521953 1.719205 0.769779

22 1 0 -0.902104 0.433670 1.545881

23 1 0 -0.628435 3.704668 -0.101453

24 1 0 -0.690877 2.997283 -1.721206

25 1 0 -3.027138 3.531138 -0.366303

26 1 0 -2.894693 2.062604 -1.343535

27 1 0 -2.351399 2.319398 1.668538

28 1 0 -3.465708 1.184980 0.888555

29 6 0 3.193702 0.740379 1.102509

30 6 0 4.040333 -0.323893 0.377344

31 6 0 -1.662374 -0.453180 -0.247874

32 8 0 -1.219881 -0.490002 -1.482036

33 7 0 -2.526432 -1.367982 0.105937

34 6 0 -2.795322 -2.497422 -0.796100

35 1 0 -3.696005 -3.002981 -0.448848

36 1 0 -2.949452 -2.141093 -1.813919

37 1 0 -1.946383 -3.187044 -0.769245

38 6 0 -2.956124 -1.556362 1.494397

39 1 0 -2.702205 -0.700773 2.113636

40 1 0 -4.037619 -1.704993 1.513911

41 1 0 -2.455921 -2.443813 1.891365

---------------------------------------------------------------------

###########################################################

**Int3a**

Standard orientation:

---------------------------------------------------------------------

Center Atomic Atomic Coordinates (Angstroms)

Number Number Type X Y Z

---------------------------------------------------------------------

1 7 0 -0.765783 1.701062 0.112761

2 1 0 1.003438 -0.274649 -1.130211

3 7 0 1.749882 -0.284640 -0.421580

4 6 0 3.074945 0.121495 -0.964314

5 6 0 1.428031 0.671938 0.693907

6 6 0 1.726534 -1.971726 0.074585

7 8 0 1.094811 -2.097761 1.107040

8 6 0 0.584321 1.828812 0.123018

9 8 0 1.131167 2.833295 -0.346950

10 8 0 2.328209 -2.629520 -0.750975

11 1 0 2.941753 0.884427 2.261627

12 1 0 2.869538 2.230918 1.111025

13 1 0 4.223230 -0.465084 0.765444

14 1 0 4.718700 1.095779 0.096176

15 1 0 3.479881 -0.702142 -1.551510

16 1 0 2.940939 1.000684 -1.600788

17 1 0 0.882038 0.100526 1.442698

18 6 0 -1.583711 0.584500 0.604158

19 6 0 -1.576907 2.757827 -0.525458

20 6 0 -2.993291 2.176949 -0.545293

21 6 0 -3.001362 1.208024 0.644810

22 1 0 -1.253760 0.284977 1.600374

23 1 0 -1.516166 3.674228 0.073125

24 1 0 -1.184590 2.984205 -1.520008

25 1 0 -3.758098 2.952827 -0.463311

26 1 0 -3.164246 1.629586 -1.477895

27 1 0 -3.119291 1.754542 1.586101

28 1 0 -3.792476 0.458769 0.583111

29 6 0 2.808889 1.146095 1.209656

30 6 0 3.856452 0.457708 0.303602

31 6 0 -1.507854 -0.626769 -0.359165

32 8 0 -0.913325 -0.519599 -1.445524

33 7 0 -2.132946 -1.770353 0.004442

34 6 0 -2.064855 -2.926784 -0.885144

35 1 0 -3.022696 -3.454303 -0.862905

36 1 0 -1.852398 -2.593892 -1.899194

37 1 0 -1.276395 -3.617461 -0.561353

38 6 0 -2.695903 -2.051748 1.321674

39 1 0 -2.686407 -1.176932 1.966364

40 1 0 -3.731540 -2.392711 1.218112

41 1 0 -2.118158 -2.846028 1.808710

---------------------------------------------------------------------

###########################################################

**TS2a**

Standard orientation:

---------------------------------------------------------------------

Center Atomic Atomic Coordinates (Angstroms)

Number Number Type X Y Z

---------------------------------------------------------------------

1 7 0 -0.310783 1.637053 -0.095923

2 1 0 -0.276967 0.015609 -1.463848

3 7 0 1.717105 -0.616924 -0.341743

4 6 0 3.063275 -0.621732 -0.935311

5 6 0 1.723130 0.430430 0.710990

6 6 0 1.153970 -1.915023 0.043360

7 8 0 0.214124 -1.850289 0.889306

8 6 0 1.054238 1.684893 0.132248

9 8 0 1.686913 2.674927 -0.229995

10 8 0 1.613766 -2.921748 -0.532680

11 1 0 3.372418 0.556745 2.160163

12 1 0 3.487553 1.698661 0.813601

13 1 0 4.150320 -1.276244 0.811670

14 1 0 4.986167 0.022701 -0.056353

15 1 0 3.223195 -1.581797 -1.424248

16 1 0 3.144546 0.179778 -1.681727

17 1 0 1.138881 0.051542 1.549886

18 6 0 -1.295757 0.783311 0.590901

19 6 0 -0.945216 2.844413 -0.684624

20 6 0 -2.444157 2.580087 -0.552891

21 6 0 -2.534399 1.712794 0.706823

22 1 0 -0.934174 0.452675 1.562564

23 1 0 -0.634550 3.725105 -0.113000

24 1 0 -0.614326 2.980567 -1.717000

25 1 0 -3.016597 3.506395 -0.467090

26 1 0 -2.820199 2.030705 -1.422933

27 1 0 -2.409160 2.322686 1.606585

28 1 0 -3.475944 1.169036 0.790574

29 6 0 3.210028 0.677553 1.085448

30 6 0 4.001890 -0.347918 0.247669

31 6 0 -1.611958 -0.458692 -0.250451

32 8 0 -1.079881 -0.556310 -1.445455

33 7 0 -2.517965 -1.345344 0.066817

34 6 0 -2.763160 -2.486787 -0.827956

35 1 0 -3.685821 -2.972429 -0.511934

36 1 0 -2.867091 -2.147239 -1.857908

37 1 0 -1.927670 -3.188704 -0.751313

38 6 0 -3.086630 -1.466880 1.412696

39 1 0 -2.743850 -0.671782 2.068272

40 1 0 -4.176917 -1.442261 1.348564

41 1 0 -2.769195 -2.426583 1.828599

---------------------------------------------------------------------

###########################################################

**TS2d**

Standard orientation:

---------------------------------------------------------------------

Center Atomic Atomic Coordinates (Angstroms)

Number Number Type X Y Z

---------------------------------------------------------------------

1 6 0 1.779448 -0.556156 2.160129

2 6 0 2.739658 -1.751945 2.004181

3 6 0 3.144653 -1.713327 0.519990

4 7 0 1.957202 -1.142962 -0.123645

5 6 0 1.010329 -0.561256 0.827121

6 6 0 1.948386 -0.527347 -1.354008

7 8 0 0.955719 0.316303 -1.468715

8 6 0 0.619947 0.817912 0.258243

9 8 0 1.506077 1.828286 0.383644

10 8 0 2.836861 -0.726747 -2.206958

11 1 0 1.118229 -0.644404 3.025437

12 1 0 2.343546 0.377244 2.251483

13 1 0 2.212819 -2.687623 2.221103

14 1 0 3.604401 -1.691366 2.669967

15 1 0 3.379248 -2.704627 0.121142

16 1 0 4.017911 -1.068559 0.354874

17 1 0 0.116526 -1.187254 0.895115

18 1 0 2.377838 1.641541 -0.059771

19 6 0 -1.824489 0.437976 0.729520

20 6 0 -1.005126 2.668034 0.133121

21 6 0 -2.532692 2.704388 0.277700

22 6 0 -2.846211 1.505157 1.184509

23 1 0 -1.583933 -0.242963 1.542350

24 1 0 -0.511838 3.318282 0.862095

25 1 0 -0.667822 2.960647 -0.864777

26 1 0 -2.878413 3.651432 0.699652

27 1 0 -3.004499 2.566862 -0.697651

28 1 0 -2.655165 1.751340 2.234207

29 1 0 -3.877429 1.153508 1.098130

30 7 0 -0.647198 1.258344 0.411024

31 6 0 -2.353711 -0.313137 -0.513400

32 8 0 -2.268123 0.212324 -1.626252

33 7 0 -2.937417 -1.532758 -0.324630

34 6 0 -3.193264 -2.181073 0.958549

35 1 0 -4.216099 -2.572774 0.960018

36 1 0 -2.507486 -3.021859 1.122191

37 1 0 -3.109788 -1.488163 1.792865

38 6 0 -3.414538 -2.276222 -1.485859

39 1 0 -3.048500 -3.307790 -1.438660

40 1 0 -4.511257 -2.300718 -1.510423

41 1 0 -3.046463 -1.798825 -2.391906

42 8 0 3.855084 1.567712 -0.938818

43 1 0 3.711667 0.829733 -1.570947

44 1 0 4.609118 1.286923 -0.397725

---------------------------------------------------------------------

###########################################################

**Int3c**

Standard orientation:

---------------------------------------------------------------------

Center Atomic Atomic Coordinates (Angstroms)

Number Number Type X Y Z

---------------------------------------------------------------------

1 6 0 -2.232299 0.281078 2.091751

2 6 0 -3.770187 0.393819 2.047202

3 6 0 -4.139757 0.038438 0.592629

4 7 0 -2.936016 0.433935 -0.154288

5 6 0 -1.817418 0.820527 0.716953

6 6 0 -2.506433 -0.167022 -1.293918

7 8 0 -1.165315 -0.039529 -1.408671

8 6 0 -0.579909 0.189663 0.009845

9 8 0 -0.220600 -1.061369 0.464632

10 8 0 -3.196469 -0.784717 -2.103910

11 1 0 -1.786096 0.844591 2.914398

12 1 0 -1.924584 -0.764571 2.182970

13 1 0 -4.078481 1.421562 2.266645

14 1 0 -4.264783 -0.264739 2.765562

15 1 0 -5.023207 0.577646 0.239669

16 1 0 -4.319641 -1.035063 0.462102

17 1 0 -1.718537 1.908372 0.744658

18 1 0 -0.843190 -1.743222 0.109599

19 6 0 1.789309 0.395640 -0.584459

20 6 0 0.440105 2.415932 -0.440329

21 6 0 1.866707 2.796537 -0.840862

22 6 0 2.396447 1.506208 -1.477372

23 1 0 1.530744 -0.483173 -1.172081

24 1 0 -0.244435 2.523460 -1.294224

25 1 0 0.065036 3.037529 0.378070

26 1 0 1.889650 3.654101 -1.518477

27 1 0 2.450107 3.042366 0.051592

28 1 0 2.009858 1.398779 -2.496543

29 1 0 3.487910 1.460101 -1.523831

30 7 0 0.558849 1.001874 -0.029622

31 6 0 2.763463 0.019233 0.550223

32 8 0 2.814015 0.693212 1.583034

33 7 0 3.597369 -1.042045 0.331164

34 6 0 3.559918 -1.900857 -0.851081

35 1 0 4.534245 -2.384534 -0.955535

36 1 0 2.797238 -2.686116 -0.766472

37 1 0 3.381823 -1.329689 -1.762570

38 6 0 4.496102 -1.474726 1.395384

39 1 0 4.225078 -2.478973 1.745210

40 1 0 5.530078 -1.504632 1.033500

41 1 0 4.421607 -0.773679 2.224566

42 8 0 -1.893170 -2.966028 -0.633480

43 1 0 -2.386127 -2.504018 -1.337387

44 1 0 -2.579679 -3.291814 -0.030838

---------------------------------------------------------------------

###########################################################

**Int4c**

Standard orientation:

---------------------------------------------------------------------

Center Atomic Atomic Coordinates (Angstroms)

Number Number Type X Y Z

---------------------------------------------------------------------

1 6 0 -1.758495 1.049705 1.900881

2 6 0 -3.250112 0.834870 2.207773

3 6 0 -3.676354 -0.202297 1.161790

4 7 0 -2.858768 0.156873 0.005549

5 6 0 -1.700317 0.949603 0.348124

6 6 0 -3.067805 -0.269556 -1.325219

7 8 0 -2.373647 0.291166 -2.202975

8 6 0 -0.417573 0.266336 -0.098505

9 8 0 -0.300326 -1.025235 -0.141814

10 8 0 -3.897843 -1.232533 -1.461427

11 1 0 -1.365336 2.002755 2.260615

12 1 0 -1.163442 0.247326 2.349804

13 1 0 -3.803554 1.767920 2.055078

14 1 0 -3.421240 0.499892 3.234436

15 1 0 -4.738932 -0.151292 0.910289

16 1 0 -3.469739 -1.223520 1.517393

17 1 0 -1.730275 1.939634 -0.117070

18 1 0 -1.156729 -1.616479 -0.096688

19 6 0 1.931042 0.322477 -0.767294

20 6 0 0.756140 2.445748 -0.348214

21 6 0 2.253227 2.706301 -0.543083

22 6 0 2.731227 1.500702 -1.364587

23 1 0 1.716391 -0.457864 -1.496389

24 1 0 0.162031 2.855810 -1.170751

25 1 0 0.374892 2.832336 0.597430

26 1 0 2.431667 3.659321 -1.045356

27 1 0 2.756939 2.727457 0.427227

28 1 0 2.461645 1.618217 -2.418794

29 1 0 3.809149 1.339949 -1.301614

30 7 0 0.656658 0.963285 -0.382049

31 6 0 2.602248 -0.263622 0.497709

32 8 0 2.249540 0.129893 1.613229

33 7 0 3.590869 -1.178286 0.312687

34 6 0 4.078723 -1.671946 -0.970448

35 1 0 5.155936 -1.486455 -1.052418

36 1 0 3.910945 -2.752814 -1.046842

37 1 0 3.588339 -1.186523 -1.810388

38 6 0 4.274096 -1.735963 1.475162

39 1 0 4.190635 -2.828780 1.468907

40 1 0 5.337799 -1.471022 1.454487

41 1 0 3.818821 -1.340705 2.380874

42 8 0 -2.142785 -2.758530 -0.255646

43 1 0 -2.935803 -2.321470 -0.708302

44 1 0 -2.467250 -3.079036 0.599275

---------------------------------------------------------------------

###########################################################

**TS2c**

Standard orientation:

---------------------------------------------------------------------

Center Atomic Atomic Coordinates (Angstroms)

Number Number Type X Y Z

---------------------------------------------------------------------

1 6 0 -2.079142 -0.386463 -2.090676

2 6 0 -3.619126 -0.423174 -2.138408

3 6 0 -4.046210 0.047590 -0.736595

4 7 0 -2.940285 -0.411039 0.110864

5 6 0 -1.774934 -0.853503 -0.656267

6 6 0 -2.622754 0.123767 1.337379

7 8 0 -1.366224 -0.083307 1.648098

8 6 0 -0.543713 -0.222672 0.024085

9 8 0 -0.266002 1.069548 -0.237110

10 8 0 -3.438114 0.779728 2.013957

11 1 0 -1.607861 -1.020544 -2.845126

12 1 0 -1.717864 0.637468 -2.227181

13 1 0 -3.965501 -1.448531 -2.307730

14 1 0 -4.034360 0.206593 -2.929373

15 1 0 -4.999494 -0.385254 -0.419215

16 1 0 -4.139825 1.140029 -0.685356

17 1 0 -1.683184 -1.941919 -0.616596

18 1 0 -1.015980 1.679856 0.006614

19 6 0 1.824253 -0.351088 0.633426

20 6 0 0.563542 -2.429976 0.350322

21 6 0 2.015945 -2.757663 0.710263

22 6 0 2.497562 -1.492340 1.431508

23 1 0 1.575856 0.497893 1.267958

24 1 0 -0.117910 -2.684194 1.171645

25 1 0 0.235268 -2.953854 -0.550769

26 1 0 2.088690 -3.656466 1.327343

27 1 0 2.598783 -2.917167 -0.201620

28 1 0 2.127128 -1.473980 2.461766

29 1 0 3.585813 -1.397988 1.458110

30 7 0 0.577139 -0.967270 0.144943

31 6 0 2.697565 0.095683 -0.559312

32 8 0 2.570616 -0.447787 -1.659734

33 7 0 3.629171 1.064954 -0.323044

34 6 0 3.811468 1.773393 0.940554

35 1 0 4.877570 1.979304 1.076343

36 1 0 3.275964 2.731898 0.945543

37 1 0 3.483052 1.180217 1.792066

38 6 0 4.442981 1.557698 -1.428607

39 1 0 4.247479 2.623447 -1.600485

40 1 0 5.508391 1.436158 -1.201541

41 1 0 4.196329 0.995960 -2.327488

42 8 0 -2.120883 2.848977 0.588370

43 1 0 -2.685458 2.366094 1.229502

44 1 0 -2.734809 3.173674 -0.088429

---------------------------------------------------------------------

###########################################################

**Int4a**

Standard orientation:

---------------------------------------------------------------------

Center Atomic Atomic Coordinates (Angstroms)

Number Number Type X Y Z

---------------------------------------------------------------------

1 6 0 -1.425958 -0.504872 1.881171

2 6 0 -2.883347 -0.109181 2.164161

3 6 0 -3.603929 -0.562476 0.900114

4 7 0 -2.644467 -0.193008 -0.193805

5 6 0 -1.257521 -0.244164 0.358939

6 6 0 -3.077467 1.293324 -0.955399

7 8 0 -2.148688 2.081282 -0.950383

8 6 0 -0.497749 -1.349991 -0.391554

9 8 0 -1.121941 -2.130367 -1.127042

10 8 0 -4.231034 1.208691 -1.337998

11 1 0 -0.709587 0.069605 2.471299

12 1 0 -1.275568 -1.566551 2.102742

13 1 0 -2.973417 0.976406 2.281824

14 1 0 -3.285175 -0.585826 3.061587

15 1 0 -4.560389 -0.078649 0.704987

16 1 0 -3.739654 -1.647972 0.888572

17 1 0 -0.812914 0.734109 0.163278

18 1 0 -2.688363 -0.897980 -0.941670

19 6 0 1.584208 -2.540342 -0.867412

20 6 0 1.703173 -0.524002 0.515923

21 6 0 2.988488 -1.361721 0.713470

22 6 0 3.052302 -2.210776 -0.565724

23 1 0 1.355845 -2.547475 -1.936076

24 1 0 1.258680 -0.240653 1.468987

25 1 0 3.874991 -0.741561 0.861624

26 1 0 2.864196 -1.997626 1.596015

27 1 0 3.481598 -1.625307 -1.383888

28 1 0 3.656894 -3.112154 -0.440795

29 7 0 0.828567 -1.466936 -0.190837

30 1 0 1.283863 -3.508878 -0.452401

31 6 0 1.969970 0.724046 -0.363246

32 7 0 2.525294 1.814447 0.236487

33 8 0 1.699102 0.692142 -1.567073

34 6 0 2.906884 1.926283 1.639669

35 1 0 2.597504 1.060652 2.219799

36 1 0 3.995037 2.031035 1.731351

37 1 0 2.441761 2.816870 2.077657

38 6 0 2.853587 2.983588 -0.572202

39 1 0 3.936546 3.157598 -0.569870

40 1 0 2.515451 2.817453 -1.592961

41 1 0 2.361591 3.873091 -0.162477

---------------------------------------------------------------------

###########################################################

**TS3a**

Standard orientation:

---------------------------------------------------------------------

Center Atomic Atomic Coordinates (Angstroms)

Number Number Type X Y Z

---------------------------------------------------------------------

1 6 0 -1.406366 -0.775721 1.812613

2 6 0 -2.898826 -0.520886 2.079181

3 6 0 -3.543099 -0.923772 0.754690

4 7 0 -2.601962 -0.386299 -0.251429

5 6 0 -1.232278 -0.423079 0.304265

6 6 0 -3.228728 1.635761 -0.920058

7 8 0 -2.188594 2.200874 -0.981959

8 6 0 -0.387272 -1.456700 -0.460790

9 8 0 -0.933534 -2.273138 -1.215557

10 8 0 -4.401549 1.484455 -1.001694

11 1 0 -0.751972 -0.180421 2.453155

12 1 0 -1.171599 -1.832047 1.982401

13 1 0 -3.077672 0.542950 2.274026

14 1 0 -3.282283 -1.092909 2.928357

15 1 0 -4.536659 -0.495715 0.596376

16 1 0 -3.620333 -2.017247 0.679874

17 1 0 -0.786394 0.570370 0.187133

18 1 0 -2.619226 -0.957126 -1.097363

19 6 0 1.793687 -2.459584 -0.951356

20 6 0 1.736944 -0.490932 0.499141

21 6 0 3.082118 -1.231744 0.687585

22 6 0 3.228116 -2.026277 -0.619553

23 1 0 1.581341 -2.455874 -2.023673

24 1 0 1.257001 -0.281449 1.454183

25 1 0 3.915401 -0.550291 0.872744

26 1 0 2.996913 -1.907880 1.544646

27 1 0 3.616776 -1.378341 -1.410472

28 1 0 3.902380 -2.880210 -0.519108

29 7 0 0.949406 -1.468859 -0.257413

30 1 0 1.566991 -3.461717 -0.569462

31 6 0 1.924650 0.808896 -0.322934

32 7 0 2.341035 1.925759 0.340299

33 8 0 1.715337 0.799631 -1.539417

34 6 0 2.701340 2.003297 1.751607

35 1 0 2.405973 1.112284 2.300097

36 1 0 3.784555 2.137049 1.866308

37 1 0 2.202884 2.865585 2.208072

38 6 0 2.604473 3.144656 -0.417207

39 1 0 3.680439 3.359058 -0.444259

40 1 0 2.237272 3.019651 -1.433868

41 1 0 2.097032 3.991994 0.056544

---------------------------------------------------------------------

###########################################################

**TS4c**

Standard orientation:

---------------------------------------------------------------------

Center Atomic Atomic Coordinates (Angstroms)

Number Number Type X Y Z

---------------------------------------------------------------------

1 6 0 1.588075 -0.997336 2.055319

2 6 0 3.051056 -0.772705 2.523900

3 6 0 3.799511 -0.285768 1.259272

4 7 0 2.766456 0.231560 0.341981

5 6 0 1.619850 -0.670329 0.530846

6 6 0 3.440117 0.355992 -1.778673

7 8 0 3.585338 -0.790735 -2.028340

8 6 0 0.323282 -0.042843 0.031199

9 8 0 0.147536 1.190035 0.050258

10 8 0 3.433215 1.529440 -1.955525

11 1 0 1.236171 -2.015840 2.236383

12 1 0 0.905116 -0.312776 2.566842

13 1 0 3.500586 -1.684889 2.926220

14 1 0 3.087685 -0.012857 3.309761

15 1 0 4.303771 -1.123023 0.763705

16 1 0 4.542581 0.490145 1.457454

17 1 0 1.836636 -1.584271 -0.031154

18 6 0 -1.938397 -0.333825 -0.826348

19 6 0 -0.591767 -2.350572 -0.458008

20 6 0 -2.040687 -2.746871 -0.772733

21 6 0 -2.590760 -1.537246 -1.543443

22 1 0 -1.762692 0.509647 -1.493462

23 1 0 0.090841 -2.668115 -1.254960

24 1 0 -0.242337 -2.771747 0.487202

25 1 0 -2.093008 -3.676210 -1.344926

26 1 0 -2.598672 -2.884042 0.157826

27 1 0 -2.254958 -1.558178 -2.585427

28 1 0 -3.681910 -1.484409 -1.537781

29 7 0 -0.643708 -0.874565 -0.398917

30 1 0 2.544766 3.002234 -0.631629

31 8 0 2.056925 3.230316 0.173872

32 1 0 2.510815 1.185555 0.606414

33 1 0 1.278203 2.641387 0.120734

34 6 0 -2.755114 0.110737 0.409785

35 7 0 -3.758349 1.012070 0.212435

36 8 0 -2.510013 -0.377757 1.516792

37 6 0 -4.119926 1.626789 -1.060180

38 1 0 -3.670926 1.110289 -1.905179

39 1 0 -5.207637 1.585071 -1.183349

40 1 0 -3.814386 2.680571 -1.086976

41 6 0 -4.541754 1.471417 1.353714

42 1 0 -4.155837 1.008046 2.259527

43 1 0 -4.473234 2.562180 1.443036

44 1 0 -5.597301 1.202739 1.226885

---------------------------------------------------------------------

###########################################################

**TS1b**

Standard orientation:

---------------------------------------------------------------------

Center Atomic Atomic Coordinates (Angstroms)

Number Number Type X Y Z

---------------------------------------------------------------------

1 6 0 3.244761 1.311272 0.042386

2 6 0 4.268074 0.172886 -0.117490

3 6 0 3.455725 -0.917266 -0.826581

4 7 0 2.125715 -0.824652 -0.213487

5 6 0 1.877135 0.569854 0.172606

6 6 0 1.651446 -1.892871 0.645623

7 8 0 0.710605 -1.575726 1.436722

8 6 0 0.859894 1.150851 -0.804028

9 8 0 1.023055 1.336948 -1.979319

10 8 0 2.194739 -3.006036 0.496166

11 1 0 3.430178 1.945031 0.912937

12 1 0 3.248094 1.948836 -0.846121

13 1 0 4.592195 -0.190485 0.864397

14 1 0 5.154951 0.487249 -0.676005

15 1 0 3.829819 -1.930195 -0.680761

16 1 0 3.402096 -0.715283 -1.905774

17 1 0 1.478702 0.593945 1.186912

18 6 0 -1.282283 0.387135 0.427145

19 6 0 -0.522607 2.722076 0.655758

20 6 0 -1.833508 2.557742 1.414632

21 6 0 -1.898188 1.047681 1.687073

22 1 0 -0.569796 -0.405450 0.698675

23 1 0 0.340569 2.648375 1.319858

24 1 0 -0.442221 3.616599 0.038218

25 1 0 -1.836795 3.152745 2.330488

26 1 0 -2.673110 2.879413 0.790383

27 1 0 -1.289177 0.786168 2.557032

28 1 0 -2.916467 0.697264 1.867710

29 7 0 -0.513101 1.509852 -0.261769

30 1 0 -1.100660 1.710609 -1.101278

31 6 0 -2.318971 -0.072416 -0.609386

32 8 0 -2.531245 0.652545 -1.599292

33 7 0 -2.961164 -1.231744 -0.378759

34 6 0 -2.699089 -2.116110 0.758768

35 1 0 -2.831340 -3.148364 0.423772

36 1 0 -1.675285 -2.013982 1.122252

37 1 0 -3.404124 -1.927274 1.576691

38 6 0 -4.051377 -1.641767 -1.261512

39 1 0 -3.806569 -2.596158 -1.739553

40 1 0 -4.971480 -1.767839 -0.680778

41 1 0 -4.203050 -0.881647 -2.025129

---------------------------------------------------------------------

###########################################################

**References**

1. Tian, Z.-Y. L., Hua, A Robust, Open-Flask, Moisture-Tolerant, and Scalable Route to Unprotected α/β-Amino Acid N-Carboxyanhydrides. *ChemRxiv. Preprint.* **2020**. https://doi.org/10.26434/chemrxiv.13489857.v1

2. Wang, X.-W.; Zhang, W.-B., Protein Catenation Enhances Both the Stability and Activity of Folded Structural Domains. *Angew. Chem. Int. Ed.* **2017,** *56* (45), 13985-13989.

3. Gaussian 09, Revision E.01, M. J. Frisch, G. W. Trucks, H. B. Schlegel, G. E. Scuseria, M. A. Robb, J. R. Cheeseman, G. Scalmani, V. Barone, B. Mennucci, G. A. Petersson, H. Nakatsuji, M. Caricato, X. Li, H. P. Hratchian, A. F. Izmaylov, J. Bloino, G. Zheng, J. L. Sonnenberg, M. Hada, M. Ehara, K. Toyota, R. Fukuda, J. Hasegawa, M. Ishida, T. Nakajima, Y. Honda, O. Kitao, H. Nakai, T. Vreven, J. A. Montgomery, Jr., J. E. Peralta, F. Ogliaro, M. Bearpark, J. J. Heyd, E. Brothers, K. N. Kudin, V. N. Staroverov, T. Keith, R. Kobayashi, J. Normand, K. Raghavachari, A. Rendell, J. C. Burant, S. S. Iyengar, J. Tomasi, M. Cossi, N. Rega, J. M. Millam, M. Klene, J. E. Knox, J. B. Cross, V. Bakken, C. Adamo, J. Jaramillo, R. Gomperts, R. E. Stratmann, O. Yazyev, A. J. Austin, R. Cammi, C. Pomelli, J. W. Ochterski, R. L. Martin, K. Morokuma, V. G. Zakrzewski, G. A. Voth, P. Salvador, J. J. Dannenberg, S. Dapprich, A. D. Daniels, O. Farkas, J. B. Foresman, J. V. Ortiz, J. Cioslowski, and D. J. Fox, Gaussian, Inc., Wallingford CT, 2013.

4. Becke, A. D., Density‐functional thermochemistry. III. The role of exact exchange. *J. Chem. Phys.* **1993,** *98* (7), 5648-5652.

5. Lee, C.; Yang, W.; Parr, R. G., Development of the Colle-Salvetti correlation-energy formula into a functional of the electron density. *Phys. Rev. B* **1988,** *37* (2), 785-789.

6. Marenich, A. V.; Cramer, C. J.; Truhlar, D. G., Universal Solvation Model Based on Solute Electron Density and on a Continuum Model of the Solvent Defined by the Bulk Dielectric Constant and Atomic Surface Tensions. *J. Phys. Chem. B* **2009,** *113* (18), 6378-6396.

7. Rassolov, V. A.; Ratner, M. A.; Pople, J. A.; Redfern, P. C.; Curtiss, L. A., 6-31G* basis set for third-row atoms. *J. Comp. Chem.* **2001,** *22* (9), 976-984.

8. Rassolov, V. A.; Pople, J. A.; Ratner, M. A.; Windus, T. L., 6-31G* basis set for atoms K through Zn. *J. Chem. Phys.* **1998,** *109* (4), 1223-1229.

9. Yao, H.; Sheng, K.; Sun, J.; Yan, S.; Hou, Y.; Lu, H.; Olsen, B. D., Secondary structure drives self-assembly in weakly segregated globular protein–rod block copolymers. *Polym. Chem.* **2020,** *11* (17), 3032-3045.

10. Hou, Y. Q.; Yuan, J. S.; Zhou, Y.; Yu, J.; Lu, H., A Concise Approach to Site-Specific Topological Protein-Poly(amino acid) Conjugates Enabled by in Situ-Generated Functionalities. *J. Am. Chem. Soc.* **2016,** *138* (34), 10995-11000.

11. Hu, Y.; Wang, D.; Wang, H.; Zhao, R.; Wang, Y.; Shi, Y.; Zhu, J.; Xie, Y.; Song, Y.-Q.; Lu, H., An urchin-like helical polypeptide-asparaginase conjugate with mitigated immunogenicity. *Biomaterials* **2021,** *268*, 120606.

12. Hou, Y. Q.; Yuan, J. S.; Zhou, Y.; Yu, J.; Lu, H., A Concise Approach to Site-Specific Topological Protein-Poly(amino acid) Conjugates Enabled by in Situ-Generated Functionalities. *J. Am. Chem. Soc.* **2016,** *138* (34), 10995-11000.

13. Hu, Y.; Hou, Y.; Wang, H.; Lu, H., Polysarcosine as an Alternative to PEG for Therapeutic Protein Conjugation. *Bioconjugate Chem.* **2018,** *29* (7), 2232-2238.
